# Intraperitoneal drain placement and outcomes after elective colorectal surgery: international matched, prospective, cohort study

**DOI:** 10.1093/bjs/znac069

**Published:** 2022-03-30

**Authors:** EuroSurg Collaborative, A Sgrò, R Blanco-Colino, WUR Ahmed, N Brindl, RR Gujjuri, P Lapolla, EC Mills, S Pérez-Ajates, AS Soares, C Varghese, W Xu, KA McLean, SJ Chapman, E Espín-Basany, JC Glasbey, A Mihaljevic, D Nepogodiev, F Pata, G Pellino, P Pockney, NN Dudi-Venkata, N Egoroff, I Ludbrook, K Raubenheimer, T Richards, P Pockney, S Delibegovic, M Salibasic, T Amjad, N Brindl, C Dörr-Harim, N Gedeon, J Gsenger, A Mihaljevic, M Tachezy, S Bini, G Gallo, A Gori, P Lapolla, F Pata, G Pellino, A Picciariello, M Podda, C Riboni, MJ Machatschek, A Nguyen, M Jakubauskas, M Kryzauskas, T Poskus, SZ Kuiper, J Wang, CI Wells, IP Bissett, KM Augestad, I Steinholt, AS Soares, BN Vieira, J Juloski, O Anabitarte Bautista, Y El Kasmy El Kasmy, S Pérez-Ajates, P Martín-Borregón, M Ossola Revilla, E Espín-Basany, S Van Straten, MK Aktas, BE Baki, WUR Ahmed, M Akhbari, D Baker, S Bhatia, S Brown, W Cambridge, SK Kamarajah, RA Khaw, O Kouli, KA McLean, EC Mills, V Murray, I Trout, I Yasin, Y Wong J, H Reyhani, KHF Wong, R Pancharatnam, WL Chia, A Walmsley, A Hassane, D Saeed, B Wang, B Walters, Z Nowinka, A Alsaif, M Mirza, K Foster, J Luu, P Kakodkar, JT Hughes, T Yogarajah, A Antypas, A Rahman, M Bradbury, M McLarnon, S Nagi, AM Riad, M Erotocritou, H Kyriacou, V Kaminskaite, S Alfadhel, Q Fatimah Hussain, A Handa, C Massy-Westropp, S Čustović, R Dimov, H Mughal, M Slavchev, T Ivanov, N Gouvas, A Hegazi, P Kocián, MD Kjaer, A Mark-Christensen, D Papakonstantinou, N Machairas, T Triantafyllou, Z Garoufalia, D Korkolis, A Castaldi, A Picciariello, S Giaccari, G Spolverato, G Pagano, M Milone, G Turri, F Colombo, E Cucinotta, G Poillucci, P Lapolla, S Bini, T Perra, R Tutino, F Belia, D Coletta, A Belli, D Rega, P Cianci, G Pirozzolo, M Di Lena, F Perrone, A Giani, F Lovisetto, M Grassia, NS Pipitone Federico, F Ferrara, A Biancafarina, N Tamini, G Sinibaldi, F Tuminello, R Galleano, D Sasia, L Bragaglia, A de Manzoni Garberini, A Pesce, F Cassaro, P Venturelli, A Gori, GL Canu, G Esposito, M Campanelli, R Cardia, M Ricciardiello, A Sagnotta, G Canonico, G De Marco, A Cappiello, E Pinotti, F Carlei, G Lisi, G Bagaglini, G Gallo, A Nguyen, MJ Machatschek, M Farrugia, EM Meima - van Praag, C Monteiro, M Pereira, P Botelho, A Quigley, A O'Neill, L Gaule, L Crone, A Arnold, F Grama, A Beuca, I Tulina, A Litvin, J Juloski, A Panyko, ME Ossola, J Trujillo Díaz, JM Marín Santos, E Alonso Batanero, S Gortázar de las Casas, C Soldevila Verdeguer, E Colás-Ruiz, I Talal El-Abur, M García Domínguez, M Delorme, M Sauvain, BB Ozmen, MK Aktas, BB Ozkan, F Calikoglu, S Kural, F Zafer, Y Kaya, A Yalcinkaya, K Kargici, MD Tepe, OC Tatar, E Kabadayi, A Yıldırım, D Hurmuzlu, K Korkmaz, P Sharma, R Troller, N Hagan, J Mooney, A Light, M Tansey, D Bhojwani, RM McGing, A Mallon, M Fadel, C Spilsbury, R James, S O’Brien, A Isaac, S Balasubramanya, H Sadik, T Gala, JY Chen, B Turner, E Goh, K Hassan, M Karam, P Mason, N Tzoumas, T Noton, JK Seehra, N Ahmed, R Motiwale, V Tanna, A Argyriou, SK Bylapudi, N Grace, S Latif, A Hounat, JS Kiam, M Zaidi, K Elsamani, C Hughes, A Suresh, LOH Sinan, D El-Dalil, EJM Khoo, EE Salim, D Stark, N Minhas, G Fowler, E Rees, I Giudiceandrea, A Bardon, P Jayawardena, N Dieseru, A Murphy, C Yates, K Ziolkowska, A Rafie, F Khoda, M Okocha, T Ashdown, P Vitish-Sharma, J Gilliland, S Toh, K Jones, A Devine, A Berry, S McDonnell, J Olivier, G Richardson, HJ Lim, P Vitish-Sharma, N Slim, K Elsayeh, T Sammour, R Dimov, A Sarpanov, N Belev, D Dimitrov, N Gouvas, T Dušek, P Kocián, MD Kjaer, A Mark-Christensen, V Ntomi, GC Sotiropoulos, D Theodorou, N Nikiteas, D Balalis, C Antropoli, DF Altomare, G Luglio, GD De Palma, C Pedrazzani, E Cucinotta, L Simonelli, S Brozzetti, A Porcu, M Massani, GL Grazi, F Izzo, P Delrio, E Restini, G Pirozzolo, G Chetta, G Lantone, G Ferrari, F Lovisetto, A Lucchi, M De Prizio, N Tamini, G Sinibaldi, R Galleano, G Caristo, F Borghi, N Petrucciani, A de Manzoni Garberini, C Huscher, G Cocorullo, V Tonini, F Medas, M Podda, G Sica, N Cillara, M Ricciardiello, A Anastasi, G De Marco, F Bianco, A Giuliani, M Carlini, F Selvaggi, G Sammarco, A Ozoliņš, A Malašonoks, P Andrejevic, P Tanis, A van de Ven, M Gerhards, B Ribeiro da Silva, A Silva, MJ Lima, D Kavanagh, N McCawley, D Kavanagh, F Grama, V Bintintan, A Karamarkovic, A Panyko, G Sanz Ortega, B De Andrés-Asenjo, C Nevado García, LJ García Flórez, JJ Segura-Sampedro, E Colás-Ruiz, JL Blas Laina, L Ponchietti, P Buchwald, E Gialamas, V Ozben, A Rencuzogullari, İE Gecim, Y Altinel, O Isik, T Yoldas, A Isik, S Leventoğlu, MS Ertürk, A Guner, SA Güler, W Attaallah, M Ugur, GS Özbalcı, H Marzook, N Eardley, S Smolarek, R Morgan, C Roxburgh, AK Lala, Y Salama, B Singh, A Khanna, M Evans, I Shaikh, K Maradi Thippeswamy, B Appleton, S Moug, I Smith, N Smart, P Shah, G Williams, G Khera, A Goede, M Varcada, C Parmar, S Duff, R Hargest, P Marriott, D Speake, A Ben Sassi, A Goede, B Furfaro, D Daudu, N Golijanin, WY Yek, G Capasso, LT Mansour, N Niu, W Seow, A Hamidovic, E Kulovic, E Letic, A Aljić, E Letic, M Helez, A Banji-Kelan, N Dimitrova, P Kavradjieva, V Ivanov, A Jukaku, D Hadzhiev, H Mughal, M Slavchev, A Gabarski, M Karamanliev, P Vladova, S Iliev, T Yotsov, Η Ευσταθίου, K Vetsa, N Gouvas, O Stavrinidou, P Papatheodorou, T Liassides, T Georgiou, A Hegazi, M Al Nassrallah, R Altaf, T Amjad, M Negametzyanov, T Dušek, D Zagibová, F Foltys, H Štefanová, A Paspala, D Papakonstantinou, G Bompetsi, T Sidiropoulos, GC Sotiropoulos, N Machairas, P Stamopoulos, A Triantafyllou, C Theodoropoulos, A Kimpizi, D Theodorou, T Triantafyllou, T Palyvou, A Charalabopoulos, A Syllaios, D Schizas, E Liatsou, E Baili, I Vagios, N Tomara, S Davakis, D Balalis, A Palumbo, A Castaldi, F Foroni, A Picciariello, DF Altomare, R Dibra, V Papagni, A Urbani, E Rossin, G Nezi, P Romano, A Amendola, E Esposito, M Manigrasso, P Anoldo, S Vertaldi, G Gecchele, G Turri, ZS Sabrina, C Guerci, F Cammarata, GMB Lamperti, G Zaffaroni, L Benuzzi, L Ferrario, M Cigognini, C Mazzeo, G Badessi, G Pintabona, A Fassari, A Mingoli, B Cirillo, C D’Alterio, G Brachini, M Tancredi, M Zambon, M Aulicino, P Sapienza, P Lapolla, P Liberatore, S Bini, AM Scanu, CF Feo, T Perra, A Iacomino, M Massani, P Pelizzo, R Tutino, S Rossi, SA Vigna, U Grossi, V Grillo, A Agnes, CA Schena, F Belia, G Marincola, A Oddi, B Perotti, D Coletta, V Mario, P Perri, S Zazza, A Aversano, D Scala, K Di Lauro, M Leongito, M Piccirillo, R Patrone, E Restini, P Cianci, S Capuzzolo, C Vignotto, G Pirozzolo, QR Bao, C Giuseppe, E Angarano, M Di Lena, F Marino, F Perrone, F Pezzolla, G Gigante, C Magistro, J Crippa, M Maspero, P Carnevali, F Lovisetto, R Trapani, S Zonta, L Agostinelli, L Vittori, L Romeo, E Doria, F Farnesi, R Danna, F Ferrara, A Biancafarina, E Andolfi, GA Pellicano’, M Angelini, M Scricciolo, C Zanframundo, C Ciulli, L Ripamonti, L Cigagna, M Oldani, N Tamini, A Larcinese, D Rossi, E Picone, G Crescentini, F Tuminello, G Caristo, A Marano, D Sasia, M Migliore, MC Giuffrida, S Palagi, V Testa, A Borrello, A Lucarini, E Garofalo, G Canali, L Bragaglia, P Orlandi, A de Manzoni Garberini, F Nervegna, F Marchegiani, I Damoli, A Licata, C Trovato, F Cassaro, F Alicata, F Sardo, M Milazzo, B Randisi, DM Dominici, G Cocorullo, P Venturelli, A Gori, L Sartarelli, M Zanni, A Pisanu, C Soddu, D Delogu, E Erdas, F Campus, F Cappellacci, F Casti, G Esposito, J Marcialis, J Atzeni, MG Podda, B Sensi, G Sica, M Franceschilli, M Campanelli, V Bellato, A Cannavera, G Putzu, N Cillara, FF di Mola, M Ricciardiello, A Sagnotta, B Picardi, L Solinas, M Loponte, S Rossi del Monte, S Rossi, C Di Martino, C Linari, G Spagni, L Capezzuoli, L Tirloni, T Nelli, A Caridi, C Elter, M Camassa, S D'Amico, T Bargellini, A Cappiello, F Bianco, P Incollingo, E Pinotti, M Montuori, F Maffione, L Romano, S Valiyeva, D Spoletini, G Lisi, M Carlini, F Menegon Tasselli, G Pellino, G Bagaglini, G Sciaudone, L Selvaggi, MP Menna, G De Paola, G Sammarco, S Fulginiti, A Truskovs, C Weiß, G Saknītis, JTR Rauscher, J Larnovskis, M Jeyarajan-Davidsson, A Malašonoks, D Nitisa, MJ Machatschek, N Gille, SC Reiser, M Farrugia, MHK Roshan, P Andrejevic, C Leseman, P Tanis, A van de Ven, J Chen, AS van Dalen, C Top, M Gerhards, R Detering, C Matos, C Monteiro, C Silva, D Pinto, J Mendes, J Couto, M Leite, C Velez, M Damasio Cotovio, AM Cinza, M Pereira, R Pedroso de Lima, P Botelho, A Quigley, E Boyle, HW Yang, I Banerjee, S Rahmat, Z Afzal, A O'Neill, C Reid, F Dumitrascu, JA Croyle, K Gressmann, N Cullen, A Graham, A Nasehi, C Montano King, L Gaule, B Martin, C Stokell, L Crone, N Sanderson, R Farnan, S jassim, A Arnold, B Chan, K Chua Vi Long, N Kaka, S Pandey, WX Neo, A Chitul, C Bezede, F Grama, A Beuca, D Cincilei, A David, M Blaga, SN Blaga, V Fagarasan, I Tulina, M Khetagurova, S Rodimov, A Kapustina, A Mekhralyzade, M Zabiyaka, J Juloski, U Janković, V Cuk, A Panyko, M Hájska, M Dubovský, M Hrošová, N Ferancikova, E Camarero Rodríguez, F Laguna Alcántara, J Adarraga, C Jezieniecki, M Ruiz Soriano, T Gómez Sanz, A Suarez, C Sánchez García, JM Marín Santos, E Alonso Batanero, I Cifrian Canales, J Llosa Pérez, M Merayo, A Urbieta, A Gegúndez Simón, JF Tone, J Gazo Martínez, M Vicario Bravo, N Chavarrias, A Gil Catalán, A Oseira, B Villalonga, C Soldevila Verdeguer, S Jeri, E Colás-Ruiz, J Perez Calvo, A Nogués, B Cros, C Yánez, I Talal El-Abur, JL Blas Laina, A Utrilla Fornals, M Roldón Golet, M García Domínguez, P Colsa, T Gimenez Maurel, M Delorme, P Buchwald, T Axmarker, E Gialamas, M Chevallay, TV Pham, BB Ozmen, EK Sel, V Ozben, C Atar, MK Aktas, M Aba, BB Ozkan, M Sarkin, YM Akkaya, AG Durmaz, F Calikoglu, HF Gullu, A Boğa, A Aktaş, B Bakar, MT Demirel, S Kural, X Hysejni, F Zafer, M Taser, OR Guzel, O Bozbiyik, A Isik, D Özen, M Ölmez, Y Kaya, B Uyar, E Gülçek, GS Kayacan, N Atıcı, OF Gul, S Altiner, B Ibis, S Altunsu, T Banaz, C Diler, I Demirbas, MA Usta, O Erkul, R Orman, S Salih, NZ Utkan, OC Tatar, SA Güler, C Acil, E Ozgur, M Maddahali, AB Turhan, AB Eskici, B Ular, M Doğru, OU Öztürk, ER Arslan, A Panahi Sharif, D Hurmuzlu, E Dikmen, J Ates, R Bircan, T Cavus, AE Sever, B Balak, E Duman, K Korkmaz, L Altay, O Emanet, F Cullen, JY Tan, P Sharma, A Nathan, A Rottenberg, CY Williams, CG Mitrofan, D Xu, JH Bawa, P Morris, R Troller, D Gordon, G Richmond, JC Hui, N Hagan, O Ighomereho, R Rocks, S McCabe, A Fitzpatrick, J Mooney, J Nicoletti, JC Hui, L Auterson, N Darrah, VWY Soh, A Light, CS Ong, M Utukuri, C Gallagher, LM Stuart, M Hipolito, N Douglas, R Ghazal, G Parris, J Catchpole, M Tansey, M Bryden, S Jamal, Z Karim, C Lyon-Dean, D Bhojwani, G Rowley, KS Lee, O Whitehurst, A Mirza, F Sheikh, H Yousaf, J Bilbao, R Sinclair, S Takar, H Kressel, RM McGing, V Chan, A Mallon, K Schack, R Osborne, S Baldemor, S Smyth, S Gilmour, A Ting, I Bozonelou, P Saunders, QA Qhaireel Anwar, R Tirimanna, S Jauhari, A Gardener, B Walker, C Spilsbury, C Wenban, H Reddy, R Conway-Jones, S Loganathan, A Clynch, C James, E Matey, F Cameron, R James, W Roberts, A Gicquel, C Milliken, J Forbes, P Rubinchik, S O’Brien, A Isaac, A Azmi, C Hawkes, L Cornett, P Adarkwah, R McConville, S O'Hara, C Tijare, J Parkes, L Yao, R Ahmad, S Balasubramanya, U Shafiq, A Mhaisalkar, A Gurung, H Sadik, K de Stadler, S Elias, T Thomas, A Madras, A Jani, HK Daler, KS Tong, SS Sundaralingam, Z Nowinka, A Szal, A Khan, C O'Sullivan, E Baker, J Joseph-Gubral, T Gala, JY Chen, B Turner, E Hadley, R Trivedi, E Igwelaezoh, E Goh, H Barton, W Allison, W Hurst, F Alam, I Parkes, K Hassan, M Jamshaid, N Azizan, T Burgher, A Afzal, I Eltilib, M Zahid, O Sadiq, A Lloyd, P Mason, R Ho, A Brazukas, CH Li, M Kamdar, MN Mohamed Nazeer, N Tzoumas, A Mighiu, D Kim, L Wilkins, L Kuo, R Conway-Jones, T Rafe, T Noton, D Maduka, H Cheema, K Farag, M Mirza, M Abdellatif, R Nzewi, A Kruczynska, H Grasselli, M Yousuff, N Ahmed, R Bassi, AK Mann, J Chopra, M Shaikh, P Sharma, S D Sa, V Tsimplis, A Ghanchi, E Skene, K Asim, M Zaheer, S Chan, H Dalton, K Gibbons, O Adderley, I Chukwujindu, I Jayasuriya, K Sivanu, M Borumand, SK Bylapudi, G Chick, I Bridges, J Tomlin, J McKenna, N Nandra, N Grace, C Grieco, FF Quek, R Mercer, S Latif, T Brankin-Frisby, A Sattar, A Aslam, E Edelsten, S Shafi, T Kouli, V Ford, F Gurung, JS Kiam, M Fernandes, N Deader, R Ponniah, S Jamieson, A Davies, J Taubwurcel, MT Aung, R Desai, S Begum, T Jamadar, A Kangatharan, B Rzeszowski, C Ho, SHK Yap, M Prendergast, R Sethi, A Duku, C Lowe, J Bray, K Elsamani, M Ghobrial, V Nichita, A Wagstaff, C Hughes, E Rengasamy, F Abu Hassan, H Mahmood, N Savill, S Shah, T Almeida, LOH Sinan, A Edwards, A Antypas, B Catchpole, D El-Dalil, Z Halford, A Carmichael, EJM Khoo, H Alsusa, EE Salim, M Boyd, C Reid, D Stark, J Williams, J Feyi-Waboso, M Patel, Z Zeidan, E Bailey, J Bapty, M Brazkiewicz, N Minhas, A Tremlett, G Fowler, H Pringle, S Mankal, V Kaminskaite, W Chung, E Rees, E Parry-Jones, K Anderson, A Mcforrester, A Stanley, A Hoather, H Wise, I Laid, I Giudiceandrea, J Scriven, A Braniste, A Wilson, L Le Blevec, N Pakunwanich, N Evans, HL Chong, C White, J Hunter, M Haque, P Vanalia, S Murdoch, T Choudhary, A McCann, A Harun, H Shah, N Dieseru, S Hunt, Y Shafiq, A Murphy, E Bickley-Morris, L Emms, M Dare, M Patel, Y Akula, C Yates, E Deliyannis, F Mayes, M Ellacott, Z Zagorac, A Farren, C Manning, C Hughed, EG Stewart, KH Lim, N Chohan, A Thaker, B Thompson, K Ziolkowska, D Ahari, E Burdekin, U Okwu, A Akintunde, F Lhaf, F Khoda, J Douthwaite, R Govindan, S Leelamanthep, E Gull, F Wright, L Dundas, M Okocha, N Mackdermott, T Burchi-Khairy, I Campbell, J Walsh, JY Yeo, S Meehan, D Banerjee, M Fu, M Kawka, T Ali, Z Hussain, C Thomas, H Ahmad, J Moroney, C Yick, R Risquet, D Ntuiabane, M Shimato, M Khan, S Ilangovan, NM Vaselli, R Smithers, R Uhanowita Marage, A Valnarov-Boulter, J Kayran, M Banerjee, N Parekh-Hill, A Hooper, J Bowen, R Jagdish, C Mcquoid, N Khan, R O Hare, S Jeffery, A Devine, A Zahid, C Elsworth, L Walter, S Dhillon, S Rao, A Anthony, A Ashaye, N Phillips, R Faderani, S Pengelly, S Choi, SY Kwak, YHL Lau, K Bagheri, R Pancharatnam, S McDonnell, DYC Ong, E Kerr, K Falconer, N Clancy, S Douglas, Y Zhang, F Greenfield, I Mutanga, J McAlinden, J Olivier, L Willis, A Adefolaju, H Agarwal, R Barter, G Harris, G Spencer, HJ Lim, MW Lee, T V Vadiveloo, G Herbert, J Moroney, C Yick, R Patel, R Risquet, M Shah, N Slim, S El Falaha, C Wong, C Soare, J Akram, K Elsayeh, L Bozhkova, Y Ma, UG Vo, HWN Tan, L Leto, MA Kamal, E Hadzhieva, P Krastev, P Tonchev, G Kokkinos, I Pozotou, D Sabbagh, J Votava, P Kocián, F St, N Koliakos, P Tsaparas, G Zografos, D Mantas, G Tsourouflis, E Fradelos, A Castaldi, G Trigiante, G Labellarte, G Resta, G Capelli, A D'Amore, V Verlingieri, T Campagnaro, A Maffioli, F Viscosi, C De Lucia, G Poillucci, S Meneghini, A Fancellu, M Colella, A Biondi, V De Peppo, U Pace, V Albino, D Gattulli, A Piangerelli, D Kalivaci, G Sisto, M Mazzola, A Caneparo, M Grassia, EG Lunghi, E Andolfi, LC Nespoli, M Angrisani, G Sinibaldi, A Langone, R Galleano, E Gelarda, E Virgilio, E Angelini, C Fornasier, S Asero, P Venturelli, E Filippone, F Frongia, PG Calò, V Bellato, P Panaccio, A Sagnotta, M Loponte, P Ipponi, S D'Amico, S Gili, A Giuliani, G Lisi, B Braccio, V Tiesi, K Stolcers, L Kokaine, V Novikovs, M Farrugia, L Capel, V Bastiaenen, H Heijmans, B Ribeiro da Silva, A Silva, P Botelho, S Henriques, SZ Gan, H Ramanayake, M Nolan, P Kakodkar, H Temperley, P Kakodkar, E Ciofic, A Beuca, BA Pop, M Kurtenkov, M Jovanović, M Vician, P Egea Arias, J Beltrán de Heredia, M Labalde Martinez, I De Santiago Alvarez, M Alvarez-Gallego, E Colás-Ruiz, I Talal El-Abur, JM Rodriguez Artigas, O Dwidar, HK Korkmaz, IC Eray, S Meriç, R Aydin, B Çetin, D Özen, A Yalcinkaya, BE Karaca, OF Kuyumcu, BE Baki, E Yüksel, TK Uprak, M Ugur, K Karabulut, E Kavukçu, A Mansor, R Troller, R Hackett, M Zammit-Maempel, R Sabaratnam, J Nicoletti, A Maan, I Ferarrio, L Dixon, H Halai, S Sethi, L Nelson, A Grassam-rowe, E Krishnan, D Deeny, M McKeever, A George Pandeth, P Dhavala, S Sreenivasan, G Sundaram Venkatesan, L Zhu, Z Atiyah, J Gregory, T Morey, Z Seymour, L Holdsworth, S Abdelmahmoud, J Bourhill, G Bisheet, J Shaw, K Kulkarni, P Kumarakulasingam, S Pillay, R Al-Habsi, G Kungwengwe, J Richards, K Davoudi, B Ibrahim, B Tailor, M Zayed, F Chen, S Bailey, S Sheefat, G Nawaz, R Pawar, S Marsh, ZH Sam, S Roy Bentley, C Simpson, J Hughes, Y Lim, R Ooi, WH Toh, P Mannion, A Lovett, A Kinčius, S Hussein, E Kirby, RG Beckett, J Salmon, A Rafie, T Glynn, SY Choo, S Lyons, D Browne, W Ravindran, S Ahmad, M Erotocritou, X Zhu, M Erotocritou, M Bradbury, J McNulty, L McCarthy, J Ng, Z Karmally, K McTeir, M Hanna, E Tan, S Namdeo, R Schembri, E Pusey

## Abstract

**Background:**

Many surgeons routinely place intraperitoneal drains after elective colorectal surgery. However, enhanced recovery after surgery guidelines recommend against their routine use owing to a lack of clear clinical benefit. This study aimed to describe international variation in intraperitoneal drain placement and the safety of this practice.

**Methods:**

COMPASS (COMPlicAted intra-abdominal collectionS after colorectal Surgery) was a prospective, international, cohort study which enrolled consecutive adults undergoing elective colorectal surgery (February to March 2020). The primary outcome was the rate of intraperitoneal drain placement. Secondary outcomes included: rate and time to diagnosis of postoperative intraperitoneal collections; rate of surgical site infections (SSIs); time to discharge; and 30-day major postoperative complications (Clavien–Dindo grade at least III). After propensity score matching, multivariable logistic regression and Cox proportional hazards regression were used to estimate the independent association of the secondary outcomes with drain placement.

**Results:**

Overall, 1805 patients from 22 countries were included (798 women, 44.2 per cent; median age 67.0 years). The drain insertion rate was 51.9 per cent (937 patients). After matching, drains were not associated with reduced rates (odds ratio (OR) 1.33, 95 per cent c.i. 0.79 to 2.23; *P* = 0.287) or earlier detection (hazard ratio (HR) 0.87, 0.33 to 2.31; *P* = 0.780) of collections. Although not associated with worse major postoperative complications (OR 1.09, 0.68 to 1.75; *P* = 0.709), drains were associated with delayed hospital discharge (HR 0.58, 0.52 to 0.66; *P* < 0.001) and an increased risk of SSIs (OR 2.47, 1.50 to 4.05; *P* < 0.001).

**Conclusion:**

Intraperitoneal drain placement after elective colorectal surgery is not associated with earlier detection of postoperative collections, but prolongs hospital stay and increases SSI risk.

## Teaser

Despite current guidelines, intraperitoneal drain placement after elective colorectal surgery remains widespread. Drains were not associated with earlier detection of intraperitoneal collections, but were associated with prolonged hospital stay and increased risk of surgical-site infections.

## Introduction

Peritoneal drains are placed after elective colorectal surgery in the historical belief that they can provide diagnostic and therapeutic benefit through prevention and early detection of anastomotic leak or other intraperitoneal collections^1,2^. However, recent evidence suggests that drains can stimulate serous fluid production, and may lead to an increased risk of surgical-site infection (SSI) and adhesions, which in turn can result in poorer postoperative pain control and mobility^3,4^. Furthermore, drains may have an impact on patient well-being owing to increased discomfort and postoperative anxiety^5^.

Recent evidence has shown no effect on measured clinical outcomes associated with drain placement after elective colorectal surgery^6–9^. Based on these findings, current enhanced recovery after surgery (ERAS) guidelines strongly recommend against the routine use of peritoneal drains after elective colorectal surgery^10^. Despite these recommendations, the use of prophylactic drains remains widespread, with data from the 2018 EuroSurg Collaborative IMAGINE (Ileus Management International) study showing that 35 per cent of participating centres routinely used intraperitoneal drains for the majority of elective colorectal procedures^11^.

The COMPASS (COMPlicAted intra-abdominal collectionS after colorectal Surgery) study aimed to describe international variation in practice regarding intraperitoneal drain placement in elective colorectal surgery, and the associated effects on postoperative outcomes.

## Methods

### Study design

COMPASS was a prospective, international, multicentre, cohort study describing international variation in intraperitoneal drain placement after colorectal surgery and the safety of this practice. The protocol was developed by an international study management group, with input from patient representatives (*Appendix S1*)^12^. This analysis was performed according to STROBE reporting guidelines for observational studies^13^.

COMPASS was delivered by a student- and trainee-led collaborative group using a collaborative model^14^. All hospitals routinely performing colorectal surgery in Europe, Australasia, and South Africa were eligible to enrol. Routine, anonymized data were collected, with no change to clinical care pathways, and confirmation of appropriate local and/or national regulatory approval was required before data collection according to country-specific regulations. Data collection took place over predefined 14-day data collection periods. Of the original five data collection periods, only the first two were completed (3 February 2020 to 8 March 2020), and the later ones cancelled because of the COVID-19 pandemic^15^. To determine the accuracy and completeness of data, an independent validation exercise was preplanned. Data accuracy was determined by assessing the accuracy of 10 planned data points (age, sex, ASA classification, previous abdominal surgery, cardiovascular disease, diabetes mellitus, operative approach, drain insertion, postoperative major Clavien–Dindo complication, SARS-CoV-2 infection); case ascertainment was determined by assessing the accuracy of participant eligibility.

### Eligibility criteria

Consecutive adults (aged at least 18 years) undergoing elective colorectal surgery for any indication (malignant or benign) were eligible. However, this excluded: operations without colorectal resection, or appendicectomies without more extensive colorectal resection; operations that were not primarily colorectal procedures (primarily urological, gynaecological or vascular procedures, or major multivisceral surgery such as pelvic exenteration); and operations without an abdominal incision (such as transanal procedures). The full list of included procedures can be found in the study protocol^12^.

In response to the COVID-19 pandemic, retrospective validation of the SARS-CoV-2 infection status of patients was conducted by a collaborator independent of the original data collection team at each site. All patients noted to have been diagnosed with a preoperative SARS-CoV-2 infection (within 7 days) were also excluded based on a positive laboratory test or chest CT, or clinical diagnosis (no laboratory test or CT chest performed)^16^. Any patients diagnosed with postoperative SARS-CoV-2 infection were still included.

### Outcome measures

The primary outcome was the rate of intraperitoneal drain placement. Secondary outcomes included: rate and time to diagnosis (measured in whole days) of intraperitoneal postoperative collections, defined as collections that altered the normal postoperative course (for example requiring either medical, radiological, endoscopic or surgical intervention)^17^; rate of 30-day drain-specific complications including SSI (Centers for Disease Control and Prevention definition^18^), cutaneous irritation at the drain site (defined by reversible damage to the skin associated with rash, dry skin, itchiness, erythema, and/or hives), small bowel evisceration and herniation of omentum (defined by prolapse of small bowel and/or omentum through the drain site after removal of the drain), and bowel injury (defined by intraoperative identification or CT-proven drain-related iatrogenic bowel perforation); overall 30-day adverse event rates defined by the highest Clavien–Dindo grade^19^; and duration of postoperative hospital stay.

### Explanatory variables

The main explanatory variable of interest was intraperitoneal drain insertion. Inserted drains were classified as either: indicated, because of a record of contaminated or dirty surgery^20^, excessive intraoperative blood loss or fluid collections (owing to lack of standardized accurate measurements, ‘excessive’ was at the discretion of the data collector based on operative notes and the surgeon’s verbal report), poor vascularization of the anastomosis, or a positive air leak test; or prophylactic, with the reason for insertion recorded as ‘surgeon preference’, ‘prophylaxis for anastomosis’, or no reason identified.

Additional variables were collected to risk-adjust outcomes for the following potential confounding factors: age; sex (M or F); smoking status (current including those who stopped smoking within 6 weeks, previous, or never); BMI (underweight (less than 18.5 kg/m^2^)–normal (18.5–24.9 kg/m^2^), overweight (25.0–30.0 kg/m^2^) or obese (more than 30.0 kg/m^2^)); ASA classification (grade I–V); cardiovascular and metabolic diseases (ischaemic heart disease, cerebrovascular disease, peripheral artery disease, and diabetes mellitus); previous abdominal surgery; immunosuppression status (defined by use of any known immunosuppressive drug, current chemotherapy or if the last chemotherapy cycle was within 12 weeks of operation); anticoagulation therapy (defined as the use of any known antiplatelet or antithrombotic agent); operative approach (open or minimally invasive) and indication (malignancy or benign); transfusion of red cells; operative contamination (clean-contaminated, contaminated or dirty^20^); and intraoperative complications (vascular or organ injury).

### Statistical analysis

Patient demographics, perioperative variables, and outcomes were compared for the three intraperitoneal drain groups (none, drain indicated, prophylactic drain). Categorical variables were cross-tabulated and compared using χ^2^ or Fisher’s exact tests. Continuous variables were summarized as median values and compared using the Kruskal–Wallis test. For time-to-event data, patients were censored at 30 days after surgery or when the event of interest or death occurred.

Mixed-effects multivariable regression was performed to derive risk-adjusted drain insertion rates, and to determine whether drain placement (prophylactic or with indication) was associated independently with the occurrence or timing of postoperative complications. Logistic regression was used for binary outcomes (occurrence of major postoperative complications, postoperative intraperitoneal collections, and SSIs) and Cox proportional hazards regression was used for time-to-event data (time to discharge, and time to diagnosis of intraperitoneal collections). For all models, clinically plausible preoperative and perioperative factors associated with drain insertion and clinical outcomes were incorporated into the modelling approach as fixed effects, and hospital was used as a random effect. Patients who had incomplete data for explanatory variables were excluded from the analysis. First-order interactions were checked and included in the model if found to be influential, with final model selection performed through minimization of the Akaike information criterion.

To investigate the association between drain placement (for any indication) and clinical outcomes, propensity score matching was used to minimize selection bias in terms of who did or did not receive intraperitoneal drains. The propensity score was defined as the probability that a patient would receive a drain based on the same model as used to determine risk-adjusted drain insertion rates. Unlike nearest-neighbour propensity score matching approaches, which can lead to inappropriate discarding of patient data, full matching was used to allow multiple patients from each group to be matched together (if appropriate) and weighted to achieve balance^21^. The balance in the preoperative and perioperative factors between groups was assessed before and after using the absolute standardized mean difference, and a value below 0.2 was considered to indicate that a variable was well balanced between groups. Subsequent doubly robust estimation^22^ was performed through risk adjustment using multivariable regression models, based on the same variables as used to generate the propensity score.

All effect estimates are presented as odds ratios (ORs) for binary outcome data and hazard ratios (HRs) for time-to-event data, with 95 per cent confidence intervals. The threshold for statistical significance was set *a priori* as *P* < 0.050. All analyses were undertaken using R version 3.4.4 (R Foundation for Statistical Computing, Vienna, Austria) with the tidyverse, finalfit, and finalpsm packages.

## Results

### Cohort characteristics

**Fig. 1:**
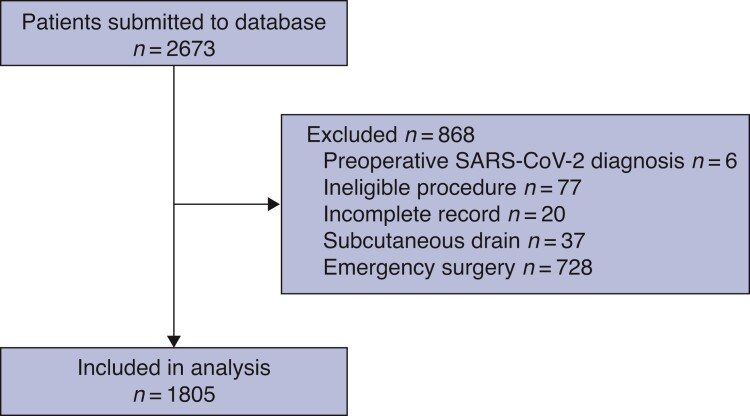
Study flow diagram

Of 2673 eligible patients from 22 countries, 1805 undergoing elective colorectal surgery were included in the analysis (798 women, 44.2 per cent; median age 67.0 years) (*Fig. 1* and *Table 1*). The most common underlying indication for surgery was malignancy (69.1 per cent), and colonic resections comprised 49.4 per cent of the cohort; rectal resections accounted for 29.8 per cent and stoma formation/closure for 20.7 per cent (*Table 2*). A full breakdown of operative procedures and indications is provided in *Tables S1* and *S2*. Overall, 937 patients (51.9 per cent) received a drain, of whom 635 (67.8 per cent) had a prophylactic drain and 302 (32.2 per cent) a drain with a defined indication. The reasons indicated for drain placement were (inserted drains could have more than 1 indication): excessive intraoperative fluid collection (146 of 353, 41.4 per cent); contaminated or dirty surgery (99 of 353, 28.0 per cent); excessive intraoperative blood loss (67 of 353, 19.0 per cent); poor vascularization of the anastomosis (35 of 353, 9.9 per cent); and a positive air leak test (6 of 353, 1.7 per cent). Data validation was performed using information on 1470 patients (81.4 per cent of the cohort), with 95.1 per cent data accuracy and 98.3 per cent case ascertainment. Propensity score matching produced balanced, well matched treatment groups (*Table S3*).

**Table 1:** Preoperative variables stratified by intraperitoneal drain placement

	No drain (*n* = 868)	Prophylactic drain (*n* = 635)	Drain with indication (*n* = 302)	Total (*n* = 1805)	*P*‡
Age (years)*	67.0 (56.0–74.0)	65.0 (54.0–74.5)	69.0 (55.0–75.0)	67.0 (55.0–74.0)	0.225§
**Sex**					0.054
F	406 (46.8)	274 (43.1)	118 (39.1)	798 (44.2)	
M	462 (53.2)	361 (56.9)	184 (60.9)	1007 (55.8)	
**Smoking status**					0.470
Never	404 (46.5)	324 (51.0)	139 (46.0)	867 (48.0)	
Previous	233 (26.8)	176 (27.7)	74 (24.5)	483 (26.8)	
Current†	113 (13.0)	81 (12.8)	49 (16.2)	243 (13.5)	
Missing	118 (13.6)	54 (8.5)	40 (13.2)	212 (11.7)	
**BMI (kg/m^2^)**					0.146
Underweight–normal	332 (38.2)	229 (36.1)	129 (42.7)	690 (38.2)	
Overweight	305 (35.1)	255 (40.2)	111 (36.8)	671 (37.2)	
Obese	187 (21.5)	138 (21.7)	52 (17.2)	377 (20.9)	
Missing	44 (5.1)	13 (2.0)	10 (3.3)	67 (3.7)	
**ASA fitness grade**					0.001
I–II	609 (70.2)	437 (68.8)	179 (59.3)	1225 (67.9)	
III–V	254 (29.3)	198 (31.2)	123 (40.7)	575 (31.9)	
Missing	5 (0.6)	0 (0)	0 (0)	5 (0.3)	
**Previous abdominal surgery**					0.133
No	423 (48.7)	328 (51.7)	135 (44.7)	886 (49.1)	
Yes	444 (51.2)	307 (48.3)	167 (55.3)	918 (50.9)	
Missing	1 (0.1)	0 (0)	0 (0)	1 (0.1)	
**Previous stoma**					0.248
No	671 (77.3)	514 (80.9)	239 (79.1)	1424 (78.9)	
Yes	196 (22.6)	121 (19.1	63 (20.9)	380 (21.1)	
Missing	1 (0.1)	0 (0)	0 (0)	1 (0.1)	
**Anticoagulation**					<0.001
No	638 (73.5)	525 (82.7)	236 (78.1)	1399 (77.5)	
Yes	230 (26.5)	109 (17.2)	66 (21.9)	405 (22.4)	
Missing	0 (0)	1 (0.2)	0 (0)	1 (0.1)	
**Diabetes mellitus**					0.697
No	739 (85.1)	531 (83.6)	251 (83.1)	1521 (84.3)	
Non-IDDM	105 (12.1)	81 (12.8)	40 (13.2)	226 (12.5)	
IDDM	20 (2.3)	20 (3.1)	11 (3.6)	51 (2.8)	
Missing	4 (0.5)	3 (0.5)	0 (0)	7 (0.4)	
**Cardiovascular disease**					0.016
No	715 (82.4)	551 (86.8)	242 (80.1)	1508 (83.5)	
Yes	153 (17.6)	84 (13.2)	60 (19.9)	297 (16.5)	
**Immunosuppression status**					0.002
No	771 (88.8)	534 (84.1)	247 (81.8)	1552 (86.0)	
Yes	96 (11.1)	100 (15.7)	55 (18.2)	251 (13.9)	
Missing	1 (0.1)	1 (0.2)	0 (0)	2 (0.1)	

Values in parentheses are percentages unless otherwise indicated; *values are median (i.q.r.). †Includes those who stopped smoking within 6 weeks. IDDM, insulin-dependent diabetes mellitus. ‡χ^2^ or Fisher’s exact test, except §Kruskal–Wallis test.

**Table 2:** Intraoperative variables stratified by intraperitoneal drain placement

	No drain (*n* = 868)	Prophylactic drain (*n* = 635)	Drain with indication (*n* = 302)	Total (*n* = 1805)	*P*†
**Underlying pathology**					0.002
Benign	302 (34.8)	168 (26.5)	86 (28.5)	556 (30.8)	
Malignancy	565 (65.1)	467 (73.5)	216 (71.5)	1248 (69.1)	
Missing	1 (0.1)	0 (0)	0 (0)	1 (0.1)	
**Perforated bowel**					0.007
No	855 (98.5)	621 (97.8)	288 (95.4)	1764 (97.7)	
Yes	13 (1.5)	14 (2.2)	14 (4.6)	41 (2.3)	
**Type of surgery**					<0.001
Colonic resection	482 (55.5)	275 (43.3)	135 (44.7)	892 (49.4)	
Rectal resection	138 (15.9)	275 (43.3)	124 (41.1)	537 (29.8)	
Stoma formation/closure	248 (28.6)	83 (13.1)	43 (14.2)	374 (20.7)	
Missing	0 (0)	2 (0.3)	0 (0)	2 (0.1)	
**Operative approach**					<0.001
Minimally invasive	522 (60.1)	308 (48.5)	113 (37.4)	943 (52.2)	
Open	346 (39.9)	326 (51.3)	189 (62.6)	861 (47.7)	
Missing	0 (0)	1 (0.2)	0 (0)	1 (0.1)	
**Operative contamination**					<0.001
Clean-contaminated	824 (94.9)	608 (95.7)	248 (82.1)	1680 (93.1)	
Contaminated/dirty	42 (4.8)	26 (4.1)	54 (17.9)	122 (6.8)	
Missing	2 (0.2)	1 (0.2)	0 (0)	3 (0.2)	
Duration of operation (min)*	160.0 (105.0–215.0)	210.0 (150.0–285.0)	200.0 (145.0–276.5)	180.0 (120–248.8)	<0.001‡
**Intraoperative anastomosis**					0.911
No	250 (28.8)	180 (28.3)	90 (29.8)	520 (28.8)	
Yes	616 (71.0)	453 (71.3)	212 (70.2)	1281 (71.0)	
Missing	2 (0.2)	2 (0.3)	0 (0)	4 (0.2)	
**Intraoperative vascular or organ injury**					<0.001
No	836 (96.3)	615 (96.9)	265 (87.7)	1716 (95.1)	
Yes	31 (3.6)	20 (3.1)	37 (12.3)	88 (4.9)	
Missing	1 (0.1)	0 (0)	0 (0)	1 (0.1)	
**Intraoperative blood transfusion**					<0.001
No	858 (98.8)	615 (96.9)	273 (90.4)	1746 (96.7)	
Yes	10 (1.2)	19 (3.0)	29 (9.6)	58 (3.2)	
Missing	0 (0)	1 (0.2)	0 (0)	1 (0.1)	

Values in parentheses are percentages unless otherwise indicated; *values are median (i.q.r.). †χ^2^ or Fisher’s exact test, except ‡Kruskal–Wallis test.

### Intraperitoneal drain placement

Patients who did not receive a drain and those who received either a prophylactic or indicated drain were comparable in terms of age, sex, smoking status, BMI, diabetes mellitus, and history of previous abdominal procedures (*Table 1*). Some differences in baseline co-morbidities were noted; patients receiving a drain with a defined indication had higher ASA grades and were more frequently immunocompromised. A primary anastomosis was created in 71.0 per cent of the cohort, with comparable rates across drain groups (*Table 2*). Patients with drains more frequently had a rectal resection, malignant pathology, an open surgical approach, contaminated or dirty operations, and more frequently had intraoperative complications.

**Fig. 2:**
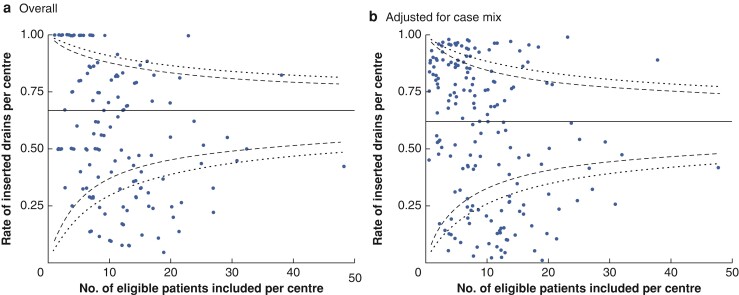
Funnel plots for rate of intraperitoneal drain placement per centre **a** Overall rate and **b** adjusted for case mix. Dots, solid lines, dashed lines, and dotted lines represent single centres, overall mean, 95% and 99% confidence intervals respectively.

Among all 937 intraperitoneal drains placed at 188 centres over the study interval, the median rate of drain placement was 67.0 (i.q.r. 37.2–100) per cent (*Fig. 2a*). This substantial variation in practice could not be explained based on case mix following adjustment using a mixed-effects logistic regression model (median 62.0 (27.2–86.4) per cent) (*Fig. 2b*).

### Postoperative outcomes

**Fig. 3:**
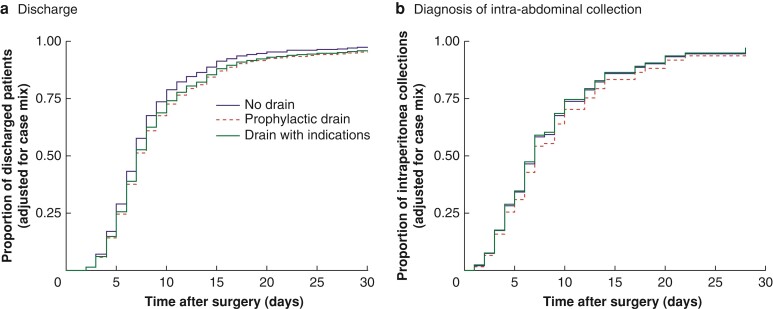
Adjusted time-to-event curves stratified by intraperitoneal drain placement **a** Time to discharge and **b** time to diagnosis of intraperitoneal collection.

On univariable analysis, the overall 30-day mortality and postoperative SARS-CoV-2 infection rates were comparable between groups. However, those who received drains had a longer postoperative hospital stay (*Table 3* and *Fig. S1a*), and this persisted on Cox proportional hazard regression, which demonstrated a lower hazard of discharge for those with prophylactic drains (HR 0.82, 95 per cent c.i. 0.71 to 0.96; *P* = 0.012) (*Fig. 3a*, *Table 4*, and *Table S4*). This association was even more pronounced following propensity score matching as patients with a drain were almost half as likely to be discharged on a given day than those without (HR 0.58, 0.52 to 0.66; *P* < 0.001) (*Table S5*).

**Table 3:** Thirty-day postoperative outcomes, by intraperitoneal drain insertion

	No drain (*n* = 868)	Intraperitoneal drain insertion				
		All drains (*n* = 937)	*P*†§	Prophylactic (*n* = 635)	Indication (*n* = 302)	*P*†¶
**Surgical-site infection**			0.022			0.059
No	798 (91.9)	837 (89.3)		566 (89.1)	271 (89.7)	
Yes	55 (6.3)	88 (9.4)		60 (9.4)	28 (9.3)	
Missing	15 (1.7)	12 (1.3)		9 (1.4)	3 (1.0)	
**Surgical-site infection at drain site**						
No	–	897 (95.7)		608 (95.8)	289 (95.7)	
Yes	–	28 (3.0)		18 (2.8)	10 (3.3)	
Missing	–	12 (1.3)		9 (1.4)	3 (1.0)	
**Postoperative intraperitoneal collections**			0.002			0.003
No	819 (94.4)	858 (91.6)		583 (91.8)	275 (91.1)	
Yes	30 (3.5)	64 (6.8)		39 (6.1)	25 (8.3)	
Missing	19 (2.2)	15 (1.6)		13 (2.0)	2 (0.7)	
Time to diagnosis of postoperative intraperitoneal collections (days)*	6.0 (4.0–9.0)	7.0 (4.0–10.0)	0.188	7.0 (4.2–12.8)‡	7.0 (4.0–9.0)	0.311‡
**Postoperative major complications (Clavien–Dindo III–V)**			0.014			0.015
No	803 (92.5)	845 (90.2)		576 (90.7)	269 (89.1)	
Yes	47 (5.4)	80 (8.5)		49 (7.7)	31 (10.3)	
Missing	18 (2.1)	12 (1.3)		10 (1.6)	2 (0.7)	
**Postoperative diagnosis of SARS-CoV-2 infection**			0.339			0.226
No	866 (99.8)	931 (99.4)		632 (99.5)	299 (99.0)	
Yes	2 (0.2)	6 (0.6)		3 (0.5)	3 (1.0)	
**Admission outcome**			0.143			0.131
Discharged	823 (94.8)	876 (93.5)		596 (93.9)	280 (92.7)	
Ongoing	22 (2.5)	38 (4.1)		22 (3.5)	16 (5.3)	
Died	5 (0.6)	3 (0.3)		1 (0.2)	2 (0.7)	
Missing	18 (2.1)	20 (2.1)		16 (2.5)	4 (1.3)	
Duration of hospital stay (days)*	6 (4–9)	8 (6–12)	<0.001‡	8 (6–12)	8 (6–13)	<0.001‡

Values in parentheses are percentages unless otherwise indicated; *values are median (i.q.r.). †χ^2^ or Fisher’s exact test, except ‡Kruskal–Wallis test. ^§^No drain *versus* drain; ^¶^no drain versus prophylactic drain *versus* drain with no indication.

**Table 4:** Summary of mixed-effects multivariable logistic and Cox proportional hazards regression models of drain-related outcomes within 30 days of surgery, before and after propensity score matching

	Univariable analysis		Multilevel analysis		Propensity score-matched analysis	
	Odds/hazard ratio	*P*	Odds/hazard ratio	*P*	Odds/hazard ratio	*P*
**Multivariable logistic regression**						
Postoperative major complications (Clavien–Dindo III–IV)						
Prophylactic drain	1.45 (0.96, 2.20)	0.077	1.16 (0.72, 1.87)	0.548		
Drain with indications	1.97 (1.22, 3.15)	0.005	1.08 (0.60, 1.93)	0.807		
All drains	1.61 (1.07, 2.45)	0.023			1.09 (0.68, 1.75)	0.709
Postoperative intraperitoneal collections						
Prophylactic drain	1.83 (1.12, 2.99)	0.015	1.64 (0.93, 2.91)	0.088		
Drain with indications	2.48 (1.42, 4.29)	0.001	1.80 (0.88, 3.67)	0.109		
All drains	2.17 (1.34, 3.64)	0.002			1.33 (0.79, 2.23)	0.287
Surgical-site infection						
Prophylactic drain	1.54 (1.05, 2.26)	0.027	1.28 (0.82, 1.99)	0.270		
Drain with indications	1.50 (0.92, 2.39)	0.095	1.17 (0.67, 2.05)	0.581		
All drains	1.57 (1.08, 2.30)	0.020			2.47 (1.50, 4.05)	<0.001
**Cox proportional hazards regression**						
Time to discharge						
Prophylactic drain	0.73 (0.66, 0.82)	<0.001	0.82 (0.71, 0.96)	0.012		
Drain with indications	0.65 (0.57, 0.75)	<0.001	0.86 (0.72, 1.03)	0.102		
All drains	0.71 (0.64, 0.79)	<0.001			0.58 (0.52, 0.66)	<0.001
Time to diagnosis of postoperative intraperitoneal collection						
Prophylactic drain	0.61 (0.37, 1.00)	0.049	0.87 (0.52, 1.47)	0.606		
Drain with indications	0.95 (0.55, 1.63)	0.851	1.03 (0.59, 1.81)	0.924		
All drains	0.78 (0.48, 1.28)	0.324			0.87 (0.33, 2.31)	0.780

Values in parentheses are 95 per cent confidence intervals. Odds ratios are shown for multivariable logistic regression analyses and hazard ratios for Cox proportional hazards regression analyses. The reference group is no drain.

Before risk adjustment, there was a higher rate of SSI (9.4 *versus* 6.3 per cent; *P* = 0.022), major postoperative complications (8.5 *versus* 5.4 per cent; *P* = 0.014), and intraperitoneal collections (6.8 *versus* 3.5 per cent; *P* = 0.002) among patients who received drains. However, there was no difference in time to diagnosis of collections (median 7.0 *versus* 6.0 days; *P* = 0.188) (*Fig. S1b*). After adjustment using mixed-effects models, none demonstrated significant differences between those who did or did not receive a drain for either prophylactic or indicated reasons (*Fig. 3b*, *Table 3* and *Tables S6–S9*). After confounding by indication had been accounted for in the propensity score-matched model, drain insertion was associated with 2.5-fold higher odds of SSI (OR 2.47, 1.50 to 4.05; *P* < 0.001) (*Table S10*). No differences were shown for major postoperative complications, postoperative intraperitoneal collections, or time to diagnosis of collections (*Tables S11–S13*).

## Discussion

Intraperitoneal drain placement in elective colorectal surgery is a longstanding yet controversial practice. RCTs and meta-analyses^6,7,9,23,24^ have demonstrated no benefit of routine drainage after elective colorectal surgery in terms of patient recovery or earlier detection of complications. However, this international prospective observational study found that intraperitoneal drain placement after elective colorectal surgery remains widespread, despite current guidelines recommending against their routine use^10^.

Intraperitoneal drain placement after elective colorectal surgery has historically been thought to prevent and improve detection of intraperitoneal complications^1,2^. Following multivariable adjustment in the present cohort, there was no difference in the odds of detection of postoperative major complications or, specifically, intraperitoneal collections for patients who had a drain inserted (overall, or whether considered indicated or prophylactic). Similarly, there was no difference in the time to diagnosis of intraperitoneal collections. Previous studies^6,8,24,25^ reached similar conclusions, and showed that drains did not decrease anastomotic leakage, morbidity, reoperation rates, and mortality after elective colorectal surgery. Therefore, COMPASS strengthens the evidence for lack of clinical benefit from routine drain placement after elective colorectal surgery.

The potential for harm from intraperitoneal drain insertion cannot be disregarded given that this remains an invasive procedure. There is evidence to suggest that drains may disrupt wound healing and even promote infection^3^. Although the occurrence of SSI in those who receive intraperitoneal drains is often heavily confounded by indication, following propensity score matching, drain insertion was associated with a 2.5-fold increased risk of SSI. In the literature, there is mixed evidence, with older studies^26,27^ suggesting no difference in SSI rates with use of drains, but more recent evidence^3,28,29^ pointing to an associated increase. Furthermore, particularly with the advent of ERAS guidance^10^, it has been recognized that the presence of drains is associated with increased pain and reduced mobility, potentially leading to increased respiratory complications^5,30^. In the present cohort, patients receiving drains had a longer hospital stay. This outcome is rarely reported in the literature; older evidence^26,31^ suggested that drain placement has no significant effect on duration of hospital stay, but more recent data^32^ suggest it can be associated with delayed hospital discharge. Although not directly assessed in this study, the use of drains could suggest overall low compliance with an ERAS protocol. Therefore, it is unclear whether the association between drain placement and longer hospital stay in this study was directly related to drain placement alone, or confounded by decreased compliance with other non-drain-related ERAS recommendations. High-quality randomized data in an ERAS context may provide definite answers to this question.

This study represents a large prospective, international data set on the topic of intraperitoneal drain insertion in elective colorectal surgery. It provides insight into the outcomes associated with both prophylactic and indication drain insertion, and provides robust adjustment for confounding by indication through propensity score matching. However, there are also several important limitations to this work. This was an observational study, with drain placement being at the discretion of the surgeon. Therefore, although selection bias regarding who received drains was accounted for, this was limited to the variables measured and so there is a persistent risk that unobserved factors may still be confounding the results. Data on decision to insert a drain were collected predominantly from clinical notes according to documentation by the surgical team. Different surgeons may have different thresholds as regards indications for drain insertion, other indications not specified in COMPASS, and also may not routinely document the specific indication in hospital records. To mitigate potential heterogeneity and disclosure bias in indication, the propensity score-matched cohort was analysed as naive to the recorded indication. Finally, it must be recognized that COMPASS overlapped with the onset of the COVID-19 pandemic outbreak. This not only limited the intended period of data collection, but also potentially introduced an unanticipated confounding factor for postoperative morbidity and mortality^16^. The impact on the present results was minimized by undertaking a validation of the included data, with assessment of SARS-CoV-2 infection rates. There were minimal recorded postoperative cases with no difference across the drain groups (*Table 3*).

Despite clear evidence and ERAS guidelines^6,7,9,10,24^, this large multicentre, international, prospective, cohort study has found that intraperitoneal drain insertion continues to remain common practice internationally in elective colorectal surgery. In the absence of clear evidence of clinical benefit, yet evidence of potential harm to patients, surgeons should ensure that any drain placed is specifically indicated (with the rationale documented). Deimplementation strategies at organizational and surgeon levels should be considered regarding the use of intraperitoneal drain placement in elective colorectal surgery^33^.

## Collaborators

Sgrò A, Blanco-Colino R, Ahmed WUR, Brindl N, Gujjuri RR, Lapolla P, Mills EC, Pérez-Ajates S, Soares AS, Varghese C, Xu W, McLean KA, Chapman SJ, Espín-Basany E, Glasbey JC, Mihaljevic A, Nepogodiev D, Pata F, Pellino G, Pockney P, Dudi-Venkata NN, Egoroff N, Ludbrook I, Raubenheimer K, Richards T, Pockney P, Delibegovic S, Salibasic M, Amjad T, Brindl N, Dörr-Harim C, Gedeon N, Gsenger J, Mihaljevic A, Tachezy M, Bini S, Gallo G, Gori A, Lapolla P, Pata F, Pellino G, Picciariello A, Podda M, Riboni C, Machatschek MJ, Nguyen A, Jakubauskas M, Kryzauskas M, Poskus T, Kuiper SZ, Wang J, Wells CI, Bissett IP, Augestad KM, Steinholt I, Soares AS, Vieira BN, Juloski J, Anabitarte Bautista O, El Kasmy El Kasmy Y, Pérez-Ajates S, Martín-Borregón P, Ossola Revilla M, Espín-Basany E, Van Straten S, Aktas MK, Baki BE, Ahmed WUR, Akhbari M, Baker D, Bhatia S, Brown S, Cambridge W, Kamarajah SK, Khaw RA, Kouli O, McLean KA, Mills EC, Murray V, Trout I, Yasin I, Wong J, Y, Reyhani H, Wong KHF, Pancharatnam R, Chia WL, Walmsley A, Hassane A, Saeed D, Wang B, Walters B, Nowinka Z, Alsaif A, Mirza M, Foster K, Luu J, Kakodkar P, Hughes JT, Yogarajah T, Antypas A, Rahman A, Bradbury M, McLarnon M, Nagi S, Riad AM, Erotocritou M, Kyriacou H, Kaminskaite V, Alfadhel S, Fatimah Hussain Q, Handa A, Massy-Westropp C, Čustović S, Dimov R, Mughal H, Slavchev M, Ivanov T, Gouvas N, Hegazi A, Kocián P, Kjaer MD, Mark-Christensen A, Papakonstantinou D, Machairas N, Triantafyllou T, Garoufalia Z, Korkolis D, Castaldi A, Picciariello A, Giaccari S, Spolverato G, Pagano G, Milone M, Turri G, Colombo F, Cucinotta E, Poillucci G, Lapolla P, Bini S, Perra T, Tutino R, Belia F, Coletta D, Belli A, Rega D, Cianci P, Pirozzolo G, Di Lena M, Perrone F, Giani A, Lovisetto F, Grassia M, Pipitone Federico NS, Ferrara F, Biancafarina A, Tamini N, Sinibaldi G, Tuminello F, Galleano R, Sasia D, Bragaglia L, de Manzoni Garberini A, Pesce A, Cassaro F, Venturelli P, Gori A, Canu GL, Esposito G, Campanelli M, Cardia R, Ricciardiello M, Sagnotta A, Canonico G, De Marco G, Cappiello A, Pinotti E, Carlei F, Lisi G, Bagaglini G, Gallo G, Nguyen A, Machatschek MJ, Farrugia M, Meima - van Praag EM, Monteiro C, Pereira M, Botelho P, Quigley A, O'Neill A, Gaule L, Crone L, Arnold A, Grama F, Beuca A, Tulina I, Litvin A, Juloski J, Panyko A, Ossola ME, Trujillo Díaz J, Marín Santos JM, Alonso Batanero E, Gortázar de las Casas S, Soldevila Verdeguer C, Colás-Ruiz E, Talal El-Abur I, García Domínguez M, Delorme M, Sauvain M, Ozmen BB, Aktas MK, Ozkan BB, Calikoglu F, Kural S, Zafer F, Kaya Y, Yalcinkaya A, Kargici K, Tepe MD, Tatar OC, Kabadayi E, Yıldırım A, Hurmuzlu D, Korkmaz K, Sharma P, Troller R, Hagan N, Mooney J, Light A, Tansey M, Bhojwani D, McGing RM, Mallon A, Fadel M, Spilsbury C, James R, O’Brien S, Isaac A, Balasubramanya S, Sadik H, Gala T, Chen JY, Turner B, Goh E, Hassan K, Karam M, Mason P, Tzoumas N, Noton T, Seehra JK, Ahmed N, Motiwale R, Tanna V, Argyriou A, Bylapudi SK, Grace N, Latif S, Hounat A, Kiam JS, Zaidi M, Elsamani K, Hughes C, Suresh A, Sinan LOH, El-Dalil D, Khoo EJM, Salim EE, Stark D, Minhas N, Fowler G, Rees E, Giudiceandrea I, Bardon A, Jayawardena P, Dieseru N, Murphy A, Yates C, Ziolkowska K, Rafie A, Khoda F, Okocha M, Ashdown T, Vitish-Sharma P, Gilliland J, Toh S, Jones K, Devine A, Berry A, McDonnell S, Olivier J, Richardson G, Lim HJ, Vitish-Sharma P, Slim N, Elsayeh K, Sammour T, Dimov R, Sarpanov A, Belev N, Dimitrov D, Gouvas N, Dušek T, Kocián P, Kjaer MD, Mark-Christensen A, Ntomi V, Sotiropoulos GC, Theodorou D, Nikiteas N, Balalis D, Antropoli C, Altomare DF, Luglio G, De Palma GD, Pedrazzani C, Cucinotta E, Simonelli L, Brozzetti S, Porcu A, Massani M, Grazi GL, Izzo F, Delrio P, Restini E, Pirozzolo G, Chetta G, Lantone G, Ferrari G, Lovisetto F, Lucchi A, De Prizio M, Tamini N, Sinibaldi G, Galleano R, Caristo G, Borghi F, Petrucciani N, de Manzoni Garberini A, Huscher C, Cocorullo G, Tonini V, Medas F, Podda M, Sica G, Cillara N, Ricciardiello M, Anastasi A, De Marco G, Bianco F, Giuliani A, Carlini M, Selvaggi F, Sammarco G, Ozoliņš A, Malašonoks A, Andrejevic P, Tanis P, van de Ven A, Gerhards M, Ribeiro da Silva B, Silva A, Lima MJ, Kavanagh D, McCawley N, Kavanagh D, Grama F, Bintintan V, Karamarkovic A, Panyko A, Sanz Ortega G, De Andrés-Asenjo B, Nevado García C, García Flórez LJ, Segura-Sampedro JJ, Colás-Ruiz E, Blas Laina JL, Ponchietti L, Buchwald P, Gialamas E, Ozben V, Rencuzogullari A, Gecim İE, Altinel Y, Isik O, Yoldas T, Isik A, Leventoğlu S, Ertürk MS, Guner A, Güler SA, Attaallah W, Ugur M, Özbalcı GS, Marzook H, Eardley N, Smolarek S, Morgan R, Roxburgh C, Lala AK, Salama Y, Singh B, Khanna A, Evans M, Shaikh I, Maradi Thippeswamy K, Appleton B, Moug S, Smith I, Smart N, Shah P, Williams G, Khera G, Goede A, Varcada M, Parmar C, Duff S, Hargest R, Marriott P, Speake D, Ben Sassi A, Goede A, Furfaro B, Daudu D, Golijanin N, Yek WY, Capasso G, Mansour LT, Niu N, Seow W, Hamidovic A, Kulovic E, Letic E, Aljić A, Letic E, Helez M, Banji-Kelan A, Dimitrova N, Kavradjieva P, Ivanov V, Jukaku A, Hadzhiev D, Mughal H, Slavchev M, Gabarski A, Karamanliev M, Vladova P, Iliev S, Yotsov T, Ευσταθίου Η, Vetsa K, Gouvas N, Stavrinidou O, Papatheodorou P, Liassides T, Georgiou T, Hegazi A, Al Nassrallah M, Altaf R, Amjad T, Negametzyanov M, Dušek T, Zagibová D, Foltys F, Štefanová H, Paspala A, Papakonstantinou D, Bompetsi G, Sidiropoulos T, Sotiropoulos GC, Machairas N, Stamopoulos P, Triantafyllou A, Theodoropoulos C, Kimpizi A, Theodorou D, Triantafyllou T, Palyvou T, Charalabopoulos A, Syllaios A, Schizas D, Liatsou E, Baili E, Vagios I, Tomara N, Davakis S, Balalis D, Palumbo A, Castaldi A, Foroni F, Picciariello A, Altomare DF, Dibra R, Papagni V, Urbani A, Rossin E, Nezi G, Romano P, Amendola A, Esposito E, Manigrasso M, Anoldo P, Vertaldi S, Gecchele G, Turri G, Sabrina ZS, Guerci C, Cammarata F, Lamperti GMB, Zaffaroni G, Benuzzi L, Ferrario L, Cigognini M, Mazzeo C, Badessi G, Pintabona G, Fassari A, Mingoli A, Cirillo B, D’Alterio C, Brachini G, Tancredi M, Zambon M, Aulicino M, Sapienza P, Lapolla P, Liberatore P, Bini S, Scanu AM, Feo CF, Perra T, Iacomino A, Massani M, Pelizzo P, Tutino R, Rossi S, Vigna SA, Grossi U, Grillo V, Agnes A, Schena CA, Belia F, Marincola G, Oddi A, Perotti B, Coletta D, Mario V, Perri P, Zazza S, Aversano A, Scala D, Di Lauro K, Leongito M, Piccirillo M, Patrone R, Restini E, Cianci P, Capuzzolo S, Vignotto C, Pirozzolo G, Bao QR, Giuseppe C, Angarano E, Di Lena M, Marino F, Perrone F, Pezzolla F, Gigante G, Magistro C, Crippa J, Maspero M, Carnevali P, Lovisetto F, Trapani R, Zonta S, Agostinelli L, Vittori L, Romeo L, Doria E, Farnesi F, Danna R, Ferrara F, Biancafarina A, Andolfi E, Pellicano’ GA, Angelini M, Scricciolo M, Zanframundo C, Ciulli C, Ripamonti L, Cigagna L, Oldani M, Tamini N, Larcinese A, Rossi D, Picone E, Crescentini G, Tuminello F, Caristo G, Marano A, Sasia D, Migliore M, Giuffrida MC, Palagi S, Testa V, Borrello A, Lucarini A, Garofalo E, Canali G, Bragaglia L, Orlandi P, de Manzoni Garberini A, Nervegna F, Marchegiani F, Damoli I, Licata A, Trovato C, Cassaro F, Alicata F, Sardo F, Milazzo M, Randisi B, Dominici DM, Cocorullo G, Venturelli P, Gori A, Sartarelli L, Zanni M, Pisanu A, Soddu C, Delogu D, Erdas E, Campus F, Cappellacci F, Casti F, Esposito G, Marcialis J, Atzeni J, Podda MG, Sensi B, Sica G, Franceschilli M, Campanelli M, Bellato V, Cannavera A, Putzu G, Cillara N, di Mola FF, Ricciardiello M, Sagnotta A, Picardi B, Solinas L, Loponte M, Rossi del Monte S, Rossi S, Di Martino C, Linari C, Spagni G, Capezzuoli L, Tirloni L, Nelli T, Caridi A, Elter C, Camassa M, D'Amico S, Bargellini T, Cappiello A, Bianco F, Incollingo P, Pinotti E, Montuori M, Maffione F, Romano L, Valiyeva S, Spoletini D, Lisi G, Carlini M, Menegon Tasselli F, Pellino G, Bagaglini G, Sciaudone G, Selvaggi L, Menna MP, De Paola G, Sammarco G, Fulginiti S, Truskovs A, Weiß C, Saknītis G, Rauscher JTR, Larnovskis J, Jeyarajan-Davidsson M, Malašonoks A, Nitisa D, Machatschek MJ, Gille N, Reiser SC, Farrugia M, Roshan MHK, Andrejevic P, Leseman C, Tanis P, van de Ven A, Chen J, van Dalen AS, Top C, Gerhards M, Detering R, Matos C, Monteiro C, Silva C, Pinto D, Mendes J, Couto J, Leite M, Velez C, Damasio Cotovio M, Cinza AM, Pereira M, Pedroso de Lima R, Botelho P, Quigley A, Boyle E, Yang HW, Banerjee I, Rahmat S, Afzal Z, O'Neill A, Reid C, Dumitrascu F, Croyle JA, Gressmann K, Cullen N, Graham A, Nasehi A, Montano King C, Gaule L, Martin B, Stokell C, Crone L, Sanderson N, Farnan R, jassim S, Arnold A, Chan B, Chua Vi Long K, Kaka N, Pandey S, Neo WX, Chitul A, Bezede C, Grama F, Beuca A, Cincilei D, David A, Blaga M, Blaga SN, Fagarasan V, Tulina I, Khetagurova M, Rodimov S, Kapustina A, Mekhralyzade A, Zabiyaka M, Juloski J, Janković U, Cuk V, Panyko A, Hájska M, Dubovský M, Hrošová M, Ferancikova N, Camarero Rodríguez E, Laguna Alcántara F, Adarraga J, Jezieniecki C, Ruiz Soriano M, Gómez Sanz T, Suarez A, Sánchez García C, Marín Santos JM, Alonso Batanero E, Cifrian Canales I, Llosa Pérez J, Merayo M, Urbieta A, Gegúndez Simón A, Tone JF, Gazo Martínez J, Vicario Bravo M, Chavarrias N, Gil Catalán A, Oseira A, Villalonga B, Soldevila Verdeguer C, Jeri S, Colás-Ruiz E, Perez Calvo J, Nogués A, Cros B, Yánez C, Talal El-Abur I, Blas Laina JL, Utrilla Fornals A, Roldón Golet M, García Domínguez M, Colsa P, Gimenez Maurel T, Delorme M, Buchwald P, Axmarker T, Gialamas E, Chevallay M, Pham TV, Ozmen BB, Sel EK, Ozben V, Atar C, Aktas MK, Aba M, Ozkan BB, Sarkin M, Akkaya YM, Durmaz AG, Calikoglu F, Gullu HF, Boğa A, Aktaş A, Bakar B, Demirel MT, Kural S, Hysejni X, Zafer F, Taser M, Guzel OR, Bozbiyik O, Isik A, Özen D, Ölmez M, Kaya Y, Uyar B, Gülçek E, Kayacan GS, Atıcı N, Gul OF, Altiner S, Ibis B, Altunsu S, Banaz T, Diler C, Demirbas I, Usta MA, Erkul O, Orman R, Salih S, Utkan NZ, Tatar OC, Güler SA, Acil C, Ozgur E, Maddahali M, Turhan AB, Eskici AB, Ular B, Doğru M, Öztürk OU, Arslan ER, Panahi Sharif A, Hurmuzlu D, Dikmen E, Ates J, Bircan R, Cavus T, Sever AE, Balak B, Duman E, Korkmaz K, Altay L, Emanet O, Cullen F, Tan JY, Sharma P, Nathan A, Rottenberg A, Williams CY, Mitrofan CG, Xu D, Bawa JH, Morris P, Troller R, Gordon D, Richmond G, Hui JC, Hagan N, Ighomereho O, Rocks R, McCabe S, Fitzpatrick A, Mooney J, Nicoletti J, Hui JC, Auterson L, Darrah N, Soh VWY, Light A, Ong CS, Utukuri M, Gallagher C, Stuart LM, Hipolito M, Douglas N, Ghazal R, Parris G, Catchpole J, Tansey M, Bryden M, Jamal S, Karim Z, Lyon-Dean C, Bhojwani D, Rowley G, Lee KS, Whitehurst O, Mirza A, Sheikh F, Yousaf H, Bilbao J, Sinclair R, Takar S, Kressel H, McGing RM, Chan V, Mallon A, Schack K, Osborne R, Baldemor S, Smyth S, Gilmour S, Ting A, Bozonelou I, Saunders P, Qhaireel Anwar QA, Tirimanna R, Jauhari S, Gardener A, Walker B, Spilsbury C, Wenban C, Reddy H, Conway-Jones R, Loganathan S, Clynch A, James C, Matey E, Cameron F, James R, Roberts W, Gicquel A, Milliken C, Forbes J, Rubinchik P, O’Brien S, Isaac A, Azmi A, Hawkes C, Cornett L, Adarkwah P, McConville R, O'Hara S, Tijare C, Parkes J, Yao L, Ahmad R, Balasubramanya S, Shafiq U, Mhaisalkar A, Gurung A, Sadik H, de Stadler K, Elias S, Thomas T, Madras A, Jani A, Daler HK, Tong KS, Sundaralingam SS, Nowinka Z, Szal A, Khan A, O'Sullivan C, Baker E, Joseph-Gubral J, Gala T, Chen JY, Turner B, Hadley E, Trivedi R, Igwelaezoh E, Goh E, Barton H, Allison W, Hurst W, Alam F, Parkes I, Hassan K, Jamshaid M, Azizan N, Burgher T, Afzal A, Eltilib I, Zahid M, Sadiq O, Lloyd A, Mason P, Ho R, Brazukas A, Li CH, Kamdar M, Mohamed Nazeer MN, Tzoumas N, Mighiu A, Kim D, Wilkins L, Kuo L, Conway-Jones R, Rafe T, Noton T, Maduka D, Cheema H, Farag K, Mirza M, Abdellatif M, Nzewi R, Kruczynska A, Grasselli H, Yousuff M, Ahmed N, Bassi R, Mann AK, Chopra J, Shaikh M, Sharma P, D Sa S, Tsimplis V, Ghanchi A, Skene E, Asim K, Zaheer M, Chan S, Dalton H, Gibbons K, Adderley O, Chukwujindu I, Jayasuriya I, Sivanu K, Borumand M, Bylapudi SK, Chick G, Bridges I, Tomlin J, McKenna J, Nandra N, Grace N, Grieco C, Quek FF, Mercer R, Latif S, Brankin-Frisby T, Sattar A, Aslam A, Edelsten E, Shafi S, Kouli T, Ford V, Gurung F, Kiam JS, Fernandes M, Deader N, Ponniah R, Jamieson S, Davies A, Taubwurcel J, Aung MT, Desai R, Begum S, Jamadar T, Kangatharan A, Rzeszowski B, Ho C, Yap SHK, Prendergast M, Sethi R, Duku A, Lowe C, Bray J, Elsamani K, Ghobrial M, Nichita V, Wagstaff A, Hughes C, Rengasamy E, Abu Hassan F, Mahmood H, Savill N, Shah S, Almeida T, Sinan LOH, Edwards A, Antypas A, Catchpole B, El-Dalil D, Halford Z, Carmichael A, Khoo EJM, Alsusa H, Salim EE, Boyd M, Reid C, Stark D, Williams J, Feyi-Waboso J, Patel M, Zeidan Z, Bailey E, Bapty J, Brazkiewicz M, Minhas N, Tremlett A, Fowler G, Pringle H, Mankal S, Kaminskaite V, Chung W, Rees E, Parry-Jones E, Anderson K, Mcforrester A, Stanley A, Hoather A, Wise H, Laid I, Giudiceandrea I, Scriven J, Braniste A, Wilson A, Le Blevec L, Pakunwanich N, Evans N, Chong HL, White C, Hunter J, Haque M, Vanalia P, Murdoch S, Choudhary T, McCann A, Harun A, Shah H, Dieseru N, Hunt S, Shafiq Y, Murphy A, Bickley-Morris E, Emms L, Dare M, Patel M, Akula Y, Yates C, Deliyannis E, Mayes F, Ellacott M, Zagorac Z, Farren A, Manning C, Hughed C, Stewart EG, Lim KH, Chohan N, Thaker A, Thompson B, Ziolkowska K, Ahari D, Burdekin E, Okwu U, Akintunde A, Lhaf F, Khoda F, Douthwaite J, Govindan R, Leelamanthep S, Gull E, Wright F, Dundas L, Okocha M, Mackdermott N, Burchi-Khairy T, Campbell I, Walsh J, Yeo JY, Meehan S, Banerjee D, Fu M, Kawka M, Ali T, Hussain Z, Thomas C, Ahmad H, Moroney J, Yick C, Risquet R, Ntuiabane D, Shimato M, Khan M, Ilangovan S, Vaselli NM, Smithers R, Uhanowita Marage R, Valnarov-Boulter A, Kayran J, Banerjee M, Parekh-Hill N, Hooper A, Bowen J, Jagdish R, Mcquoid C, Khan N, O Hare R, Jeffery S, Devine A, Zahid A, Elsworth C, Walter L, Dhillon S, Rao S, Anthony A, Ashaye A, Phillips N, Faderani R, Pengelly S, Choi S, Kwak SY, Lau YHL, Bagheri K, Pancharatnam R, McDonnell S, Ong DYC, Kerr E, Falconer K, Clancy N, Douglas S, Zhang Y, Greenfield F, Mutanga I, McAlinden J, Olivier J, Willis L, Adefolaju A, Agarwal H, Barter R, Harris G, Spencer G, Lim HJ, Lee MW, V Vadiveloo T, Herbert G, Moroney J, Yick C, Patel R, Risquet R, Shah M, Slim N, El Falaha S, Wong C, Soare C, Akram J, Elsayeh K, Bozhkova L, Ma Y, Vo UG, Tan HWN, Leto L, Kamal MA, Hadzhieva E, Krastev P, Tonchev P, Kokkinos G, Pozotou I, Sabbagh D, Votava J, Kocián P, St F, Koliakos N, Tsaparas P, Zografos G, Mantas D, Tsourouflis G, Fradelos E, Castaldi A, Trigiante G, Labellarte G, Resta G, Capelli G, D'Amore A, Verlingieri V, Campagnaro T, Maffioli A, Viscosi F, De Lucia C, Poillucci G, Meneghini S, Fancellu A, Colella M, Biondi A, De Peppo V, Pace U, Albino V, Gattulli D, Piangerelli A, Kalivaci D, Sisto G, Mazzola M, Caneparo A, Grassia M, Lunghi EG, Andolfi E, Nespoli LC, Angrisani M, Sinibaldi G, Langone A, Galleano R, Gelarda E, Virgilio E, Angelini E, Fornasier C, Asero S, Venturelli P, Filippone E, Frongia F, Calò PG, Bellato V, Panaccio P, Sagnotta A, Loponte M, Ipponi P, D'Amico S, Gili S, Giuliani A, Lisi G, Braccio B, Tiesi V, Stolcers K, Kokaine L, Novikovs V, Farrugia M, Capel L, Bastiaenen V, Heijmans H, Ribeiro da Silva B, Silva A, Botelho P, Henriques S, Gan SZ, Ramanayake H, Nolan M, Kakodkar P, Temperley H, Kakodkar P, Ciofic E, Beuca A, Pop BA, Kurtenkov M, Jovanović M, Vician M, Egea Arias P, Beltrán de Heredia J, Labalde Martinez M, De Santiago Alvarez I, Alvarez-Gallego M, Colás-Ruiz E, Talal El-Abur I, Rodriguez Artigas JM, Dwidar O, Korkmaz HK, Eray IC, Meriç S, Aydin R, Çetin B, Özen D, Yalcinkaya A, Karaca BE, Kuyumcu OF, Baki BE, Yüksel E, Uprak TK, Ugur M, Karabulut K, Kavukçu E, Mansor A, Troller R, Hackett R, Zammit-Maempel M, Sabaratnam R, Nicoletti J, Maan A, Ferarrio I, Dixon L, Halai H, Sethi S, Nelson L, Grassam-rowe A, Krishnan E, Deeny D, McKeever M, George Pandeth A, Dhavala P, Sreenivasan S, Sundaram Venkatesan G, Zhu L, Atiyah Z, Gregory J, Morey T, Seymour Z, Holdsworth L, Abdelmahmoud S, Bourhill J, Bisheet G, Shaw J, Kulkarni K, Kumarakulasingam P, Pillay S, Al-Habsi R, Kungwengwe G, Richards J, Davoudi K, Ibrahim B, Tailor B, Zayed M, Chen F, Bailey S, Sheefat S, Nawaz G, Pawar R, Marsh S, Sam ZH, Roy Bentley S, Simpson C, Hughes J, Lim Y, Ooi R, Toh WH, Mannion P, Lovett A, Kinčius A, Hussein S, Kirby E, Beckett RG, Salmon J, Rafie A, Glynn T, Choo SY, Lyons S, Browne D, Ravindran W, Ahmad S, Erotocritou M, Zhu X, Erotocritou M, Bradbury M, McNulty J, McCarthy L, Ng J, Karmally Z, McTeir K, Hanna M, Tan E, Namdeo S, Schembri R, Pusey E.

## Supplementary Material

znac069_Supplementary_Data
