# Supplementary material for: Intraperitoneal drain placement and outcomes after elective colorectal surgery: international matched, prospective, cohort study
Source: Br J Surg. 2022 Mar 30;109(6):520–9. doi: 10.1093/bjs/znac069 (PMC10364732; doi:10.1093/bjs/znac069)
Supplement: znac069_Supplementary_Data [file znac069_supplementary_data.docx]

**International variation in intraperitoneal drain placement and outcomes after elective colorectal surgery: a matched, prospective, cohort study**

EuroSurg Collaborative

**Appendix S1 Collaborating members of the EuroSurg Collaborative**

The authorship list below is a list of all collaborators according to their study roles; some names may be duplicated to reflect multiple roles.

WRITING GROUP

Sgrò A, Blanco-Colino R, Ahmed WUR, Brindl N, Gujjuri RR, Lapolla P, Mills EC, Pérez-Ajates S, Varghese C, Xu W, McLean KA.

STUDY MANAGEMENT GROUP

Sgrò A, Blanco-Colino R, Ahmed WUR, Brindl N, Gujjuri RR, Lapolla P, Mills EC, Pérez-Ajates S, Soares AS, Varghese C, Xu W, McLean KA.

EXPERT ADVISORY GROUP

Chapman SJ, Espín-Basany E, Glasbey JC, Mihaljevic A, Nepogodiev D, Pata F, Pellino G, Pockney P.

DATA ANALYSIS

Sgrò A, McLean KA.

NATIONAL LEADS

Dudi-Venkata NN, Egoroff N, Ludbrook I, Raubenheimer K, Richards T, Pockney P (**Australia**); Delibegovic S, Salibasic M (**Bosnia and Herzegovina**); Amjad T (**Czech Republic**); Brindl N, Dörr-Harim C, Gedeon N, Gsenger J, Mihaljevic A, Tachezy M (**Germany**); Bini S, Gallo G, Gori A, Lapolla P, Pata F, Pellino G, Picciariello A, Podda M, Riboni C (**Italy**); Machatschek MJ, Nguyen A (**Latvia**); Jakubauskas M, Kryzauskas M, Poskus T (**Lithuania**); Kuiper SZ (**The Netherlands**); Wang J, Wells CI, Bissett IP (**New Zealand**); Augestad KM, Steinholt I (**Norway**); Soares AS, Vieira BN (**Portugal**); Juloski J (**Serbia**); Anabitarte Bautista O, El Kasmy El Kasmy Y, Pérez-Ajates S, Martín-Borregón P, Ossola Revilla M, Espín-Basany E (**Spain**); Van Straten S (**South Africa**); Aktas MK, Baki BE (**Turkey**); Ahmed WUR, Akhbari M, Baker D, Bhatia S, Brown S, Cambridge W, Kamarajah SK, Khaw RA, Kouli O, McLean KA, Mills EC, Murray V, Trout I, Yasin I (**UK and Republic of Ireland**).

REGIONAL LEADS (UK & IRELAND ONLY)

Wong J (Aberdeen Medical School); Bharwada Y (Birmingham University Medical School); Reyhani H (Brighton and Sussex Medical School); Wong KHF (Bristol University Medical School); Pancharatnam R (Buckingham University Medical School); Chia WL (Cardiff University School of Medicine); Walmsley A (Dublin University Medical School); Hassane A (Dundee University Medical School); Saeed D (Hull York Medical School); Wang B (Imperial College School of Medicine); Walters B (Keele University Medical School); Nowinka Z (Lancaster University Medical School); Alsaif A (Leeds University Medical School); Mirza M (Leicester University Medical School); Foster K (Liverpool University Medical School); Luu J (Manchester University Medical School); Kakodkar P (National University Ireland School of Medicine); Hughes JT (Newcastle University Medical School); Yogarajah T (Norwich Medical School); Antypas A (Nottingham University Medical School); Rahman A (Oxford University Medical School); Bradbury M (Plymouth University Medical School); McLarnon M (Queen’s University of Belfast Medical School); Nagi S (St George's University Medical School); Riad AM (The University of Edinburgh Medical School); Erotocritou M (UCL Medical School); Kyriacou H (University of Cambridge School of Clinical Medicine); Kaminskaite V (University of Exeter Medical School); Alfadhel S (University of Glasgow Medical School); Fatimah Hussain Q (Warwick University Medical School).

LOCAL LEADS

**Australia:** Handa A (Fiona Stanley Hospital); Massy-Westropp C (Royal Adelaide Hospital).

**Bosnia & Herzegovina:** Salibasic M (Clinical Center University of Sarajevo); Čustović S (General Hospital Abdulah Nakas).

**Bulgaria:** Dimov R (Kaspela University Hospital); Mughal H (St. George Hospital); Slavchev M (University Hospital Eurohospital); Ivanov T (University Hospital of Medical University - Pleven, Department of Surgical Oncology).

**Cyprus:** Gouvas N (Nicosia General Hospital).

**Czech Republic:** Hegazi A (Fakultní Nemocnice Hradec Králové); Kocián P (Motol University Hospital).

**Denmark:** Kjaer MD (Odense Universitetshospital); Mark-Christensen A (Svendborg Sygehus)

**Greece:** Papakonstantinou D (Attikon University Hospital); Machairas N (Evgenideio Hospital); Triantafyllou T (Hippocration General Hospital Of Athens, National And Kapodistrian University Of Athens); Garoufalia Z (Laiko General Hospital); Korkolis D (Saint Savvas Anticancer Hospital).

**Italy:** Castaldi A (AORN A. Cardarelli); Picciariello A (AOUC Policlinico di Bari); Giaccari S (Arcispedale Sant'Anna, Università degli studi di Ferrara); Spolverato G (Azienda Ospedaliera di Padova); Pagano G, Milone M (Azienda Ospedaliera Universitaria Federico II, Napoli); Turri G (Azienda Ospedaliera Universitaria Integrata Verona); Colombo F (Azienda Ospedaliera Universitaria L. Sacco, Milano); Cucinotta E (Azienda Ospedaliera Universitaria Policlinico 'G. Martino', Messina); Poillucci G, Lapolla P, Bini S (Azienda Ospedaliera Universitaria Policlinico Umberto I, Roma); Perra T (Azienda Ospedaliero Universitaria di Sassari); Tutino R ("Ca Foncello" hospital); Belia F (Fondazione Policlinico Universitario A. Gemelli, Roma); Coletta D (IRCCS Regina Elena National Cancer Institute); Belli A, Rega D (Istituto Nazionale per lo Studio e la Cura dei Tumori Fondazione Giovanni Pascale IRCCS, Napoli); Cianci P (Lorenzo Bonomo Hospital); Pirozzolo G (Mestre hospital); Di Lena M ("Monsignor Dimiccoli" Hospital); Perrone F (National Institute of Gastroenterology Research Hospital); Giani A (Niguarda Hospital, Milan); Lovisetto F (Ospedale Castelli, Verbania); Grassia M (Ospedale Ceccarini, Riccione); Pipitone Federico NS (Ospedale Civile Edoardo Agnelli, Pinerolo); Ferrara F (Ospedale San Carlo Borromeo, Milano); Biancafarina A (Ospedale San Donato, Arezzo); Tamini N (Ospedale San Gerardo, Monza); Sinibaldi G (Ospedale San Giovanni Calibita Fatebenefralli, Roma); Tuminello F (Ospedale San Paolo, Savona); Galleano R (Ospedale Santa Corona, Pietra Ligure); Sasia D (Ospedale Santa Croce e Carle, Cuneo); Bragaglia L (Ospedale Sant'Andrea, Roma); de Manzoni Garberini A (Ospedale 'Spirito Santo' di Pescara); Pesce A (Policlinic Hospital Abano Terme - Department of Surgical Oncology, Robotics and New Technologies); Cassaro F (Policlinico 'Gaspare Rodolico', Catania); Venturelli P (Policlinico 'P. Giaccone', Palermo); Gori A (Policlinico Sant'Orsola-Malpighi, Bologna); Canu GL, Esposito G (Policlinico Universitario di Monserrato 'Duilio Casula'); Campanelli M (Polyclinic Tor Vergata); Cardia R (PO SS. Trinità, Cagliari); Ricciardiello M (Private hospital Pierangeli); Sagnotta A (San Filippo Neri Hospital, Rome); Canonico G (San Giovanni di Dio Hospital); De Marco G (San Giuseppe, Empoli); Cappiello A (San Leonardo Hospital,ASL-NA3 Sud Castellammare di Stabia-Naples); Pinotti E (San Pietro Hospital); Carlei F (San Salvatore Hospital); Lisi G (Sant'Eugenio Hospital); Bagaglini G (Università degli Studi della Campania 'Luigi Vanvitelli', Napoli); Gallo G (U.O. di Chirurgia Generale, Policlinico Universitario di Catanzaro).

**Latvia:** Nguyen A (Paula Stradiņa Klīniskā universitātes slimnīca); Machatschek MJ (Rīgas Austrumu klīniskā universitātes slimnīca).

**Malta:** Farrugia M (Sptar Mater Dei).

**Netherlands:** Meima - van Praag EM (Academic Medical Centre); Meima - van Praag EM (Flevoziekenhuis); Meima - van Praag EM (Onze Lieve Vrouwe Gasthuis (OLVG) locatie oost).

**Portugal:** Monteiro C (Hospital de Santa Luzia); Pereira M (Hospital Espírito Santo Évora, E.P.E.); Botelho P (Hospital Garcia de Orta).

**Republic of Ireland:** Quigley A (Adelaide and Meath Hospital, Tallaght); O'Neill A (Beaumont Hospital, Dublin); Gaule L (Mayo University Hospital); Crone L (St James's Hospital, Dublin); Arnold A (University Hospital Galway).

**Romania:** Grama F (Coltea Clinical Hospital); Beuca A (County Emergency Hospital Cluj-Napoca).

**Russia:** Tulina I (Clinic of Colorectal and Minimally Invasive Surgery - Sechenov University); Litvin A (Immanuel Kant Baltic Federal University, Kaliningrad Regional Clinical Hospital).

**Serbia:** Juloski J (Zvezdara University Medical Center, Belgrade).

**Slovakia:** Panyko A (Univerzitná nemocnica Bratislava Ružinov).

**Spain:** Ossola ME (Hospital Clínico San Carlos); Trujillo Díaz J (Hospital Clínico Universitario Valladolid); Marín Santos JM (Hospital Universitario 12 de Octubre); Alonso Batanero E (Hospital Universitario Central de Asturias); Gortázar de las Casas S (Hospital Universitario La Paz); Soldevila Verdeguer C (Hospital Universitario Son Espases); Colás-Ruiz E (Manacor Hospital); Talal El-Abur I (Royo Villanova Hospital); García Domínguez M (San Jorge Hospital).

**Sweden:** Delorme M (Skåne University Hospital, SUS, Malmö).

**Switzerland:** Sauvain M (Hôpital neuchâtelois).

**Turkey:** Ozmen BB (Acıbadem Mehmet Ali Aydınlar Üniversitesi Atakent Hastanesi); Aktas MK (Adana Çukurova Üniversitesi Tıp Fakültesi Balcalı Hastanesi); Ozkan BB (Ankara Üniversitesi Tıp Fakültesi); Calikoglu F (Bagcilar research and training hospital, health sciences university); Kural S (Bursa Uludag University Hospital of Faculty of Medicine); Zafer F (Ege University Medical Faculty Hospital); Kaya Y (Erzincan Üniversitesi Mengücek Gazi Eğitim ve Araştırma Hastanesi); Yalcinkaya A (Gazi Universitesi Hastanesi); Kargici K (İstanbul Üniversitesi- Cerrahpaşa Tıp Fakültesi); Tepe MD (Karadeniz Teknik Universitesi Tıp Fakültesi Hastanesi); Tatar OC (Kocaeli University Teaching Hospital); Kabadayi E (Marmara University Research And Education Hospital); Yıldırım A (Mustafa Kemal University Research And Practice Hospital); Hurmuzlu D (Ondokuz Mayıs Üniversitesi Tıp Fakültesi Hastanesi); Korkmaz K (Yeditepe Universitesi Hastanesi).

**United Kingdom:** Sharma P (Aberdeen Royal Infirmary); Troller R (Addenbrooke's Hospital); Hagan N (Altnagelvin Area Hospital); Mooney J (Antrim Area Hospital); Light A (Bedford Hospital); Tansey M (Blackpool Victoria Hospital); Bhojwani D (Bristol Royal Infirmary); McGing RM (Castle Hill Hospital); Mallon A (Causeway Hospital); Fadel M (Chelsea and Westminster Hospital); Spilsbury C (Churchill Hospital); James R (Countess of Chester Hospital); O’Brien S (Craigavon Area Hospital); Isaac A (Daisy Hill Hospital); Balasubramanya S (Derriford Hospital); Sadik H (Ealing Hospital); Gala T (Glan Clwyd Hospital, Rhyl); Chen JY (Glasgow Royal Infirmary); Turner B (Gloucestershire Royal Hospital); Goh E (Great Western Hospital, Swindon); Hassan K (Gwynedd Hospital); Karam M (Huddersfield Royal Infirmary); Mason P (Hull Royal Infirmary ); Tzoumas N (James Cook University Hospital); Noton T (John Radcliffe Hospital); Seehra JK (Kettering General Hospital); Ahmed N (King's Mill Hospital); Motiwale R (Leicester General Hospital); Tanna V (Leicester Royal Infirmary); Argyriou A (Manchester Royal Infirmary); Bylapudi SK (Milton Keynes University Hospital); Grace N (Morriston Hospital); Latif S (Musgrove Park Hospital); Hounat A (Ninewells Hospital); Kiam JS (Norfolk and Norwich University Hospital); Zaidi M (Northwick Park/St. Mark's Hospitals); Elsamani K (Peterborough City Hospital); Hughes C (Prince Charles Hospital, Merthyr); Suresh A (Princess of Wales, Bridgend); Sinan LOH (Queen Elizabeth University Hospital, Glasgow); El-Dalil D (Queens Medical Centre, Nottingham); Khoo EJM (Royal Albert Edward Infirmary (Wigan Infirmary)); Salim EE (Royal Alexandra Hospital, Paisley); Stark D (Royal Cornwall Hospital ); Minhas N (Royal Derby Hospital); Fowler G (Royal Devon and Exeter Hospital); Rees E (Royal Glamorgan Hospital); Giudiceandrea I (Royal Gwent Hospital); Bardon A (Royal Lancaster Hospital); Jayawardena P (Royal Preston Hospital); Dieseru N (Royal Stoke University Hospital); Murphy A (Royal Sussex County Hospital); Yates C (Royal United Hospital); Ziolkowska K (Royal Victoria Infirmary, Newcastle); Rafie A (Salford Royal Hospital); Khoda F (Scunthorpe General Hospital ); Okocha M (Southmead Hospital, Bristol); Ashdown T (St. Mary's Hospital, London); Vitish-Sharma P (Stoke Mandeville Hospital); Gilliland J (The Royal Free Hospital); Toh S (The Royal Liverpool University Hospital); Jones K (Ulster Hospital); Devine A (University Hospital of South Manchester); Berry A (University Hospital of Wales); McDonnell S (Warwick Hospital); Olivier J (Weston General Hospital); Richardson G (Whiston Hospital); Lim HJ (Wrexham Maelor Hospital); Vitish-Sharma P (Wycombe General Hospital); Slim N (Yeovil District Hospital); Elsayeh K (York Hospital).

SUPERVISING CONSULTANT/ATTENDING

**Australia:** Richards T (Fiona Stanley Hospital); Sammour T (Royal Adelaide Hospital).

**Bosnia & Herzegovina:** Salibasic M (Clinical Center University of Sarajevo); Čustović S (General Hospital Abdulah Nakas).

**Bulgaria:** Dimov R (Kaspela University Hospital); Sarpanov A (St. George Hospital); Belev N (University Hospital Eurohospital); Dimitrov D (University Hospital of Medical University - Pleven, Department of Surgical Oncology).

**Cyprus:** Gouvas N (Nicosia General Hospital).

**Czech Republic:** Dušek T (Fakultní Nemocnice Hradec Králové); Kocián P (Motol University Hospital).

**Denmark:** Kjaer MD (Odense Universitetshospital); Mark-Christensen A (Svendborg Sygehus).

**Greece:** Ntomi V (Attikon University Hospital); Sotiropoulos GC (Evgenideio Hospital); Theodorou D (Hippocration General Hospital Of Athens, National And Kapodistrian University Of Athens); Nikiteas N (Laiko General Hospital); Balalis D (Saint Savvas Anticancer Hospital).

**Italy:** Antropoli C (AORN A. Cardarelli); Altomare DF (AOUC Policlinico di Bari); Spolverato G (Azienda Ospedaliera di Padova); Luglio G, De Palma GD (Azienda Ospedaliera Universitaria Federico II, Napoli); Pedrazzani C (Azienda Ospedaliera Universitaria Integrata Verona); Cucinotta E (Azienda Ospedaliera Universitaria Policlinico 'G. Martino', Messina); Simonelli L, Brozzetti S (Azienda Ospedaliera Universitaria Policlinico Umberto I, Roma); Porcu A (Azienda Ospedaliero Universitaria di Sassari); Massani M ("Ca Foncello" hospital); Grazi GL (IRCCS Regina Elena National Cancer Institute); Izzo F, Delrio P (Istituto Nazionale per lo Studio e la Cura dei Tumori Fondazione Giovanni Pascale IRCCS, Napoli); Restini E (Lorenzo Bonomo Hospital); Pirozzolo G (Mestre hospital); Chetta G ("Monsignor Dimiccoli" Hospital); Lantone G (National Institute of Gastroenterology Research Hospital); Ferrari G (Niguarda Hospital, Milan); Lovisetto F (Ospedale Castelli, Verbania); Lucchi A (Ospedale Ceccarini, Riccione); Pipitone Federico NS (Ospedale Civile Edoardo Agnelli, Pinerolo); De Prizio M (Ospedale San Donato, Arezzo); Tamini N (Ospedale San Gerardo, Monza); Sinibaldi G (Ospedale San Giovanni Calibita Fatebenefralli, Roma); Galleano R (Ospedale San Paolo, Savona); Caristo G (Ospedale Santa Corona, Pietra Ligure); Borghi F (Ospedale Santa Croce e Carle, Cuneo); Petrucciani N (Ospedale Sant'Andrea, Roma); de Manzoni Garberini A (Ospedale 'Spirito Santo' di Pescara); Huscher C (Policlinic Hospital Abano Terme - Department of Surgical Oncology, Robotics and New Technologies); Cocorullo G (Policlinico 'P. Giaccone', Palermo); Tonini V (Policlinico Sant'Orsola-Malpighi, Bologna); Medas F, Podda M (Policlinico Universitario di Monserrato 'Duilio Casula'); Sica G (Polyclinic Tor Vergata); Cillara N (PO SS. Trinità, Cagliari); Ricciardiello M (Private hospital Pierangeli); Anastasi A (San Giovanni di Dio Hospital); De Marco G (San Giuseppe, Empoli); Bianco F (San Leonardo Hospital,ASL-NA3 Sud Castellammare di Stabia-Naples); Giuliani A (San Salvatore Hospital); Carlini M (Sant'Eugenio Hospital); Selvaggi F (Università degli Studi della Campania 'Luigi Vanvitelli', Napoli); Sammarco G (U.O. di Chirurgia Generale, Policlinico Universitario di Catanzaro).

**Latvia:** Ozoliņš A (Paula Stradiņa Klīniskā universitātes slimnīca); Malašonoks A (Rīgas Austrumu klīniskā universitātes slimnīca).

**Malta:** Andrejevic P (Sptar Mater Dei).

**Netherlands:** Tanis P (Academic Medical Centre); van de Ven A (Flevoziekenhuis); Gerhards M (Onze Lieve Vrouwe Gasthuis (OLVG) locatie oost).

**Portugal:** Ribeiro da Silva B (Hospital de Santa Luzia); Silva A (Hospital Espírito Santo Évora, E.P.E.); Lima MJ (Hospital Garcia de Orta).

**Republic of Ireland:** Kavanagh D (Adelaide and Meath Hospital, Tallaght); McCawley N (Beaumont Hospital, Dublin); Kavanagh D (St James's Hospital, Dublin).

**Romania:** Grama F (Coltea Clinical Hospital); Bintintan V (County Emergency Hospital Cluj-Napoca).

**Russia:** Tulina I (Clinic of Colorectal and Minimally Invasive Surgery - Sechenov University); Litvin A (Immanuel Kant Baltic Federal University, Kaliningrad Regional Clinical Hospital).

**Serbia:** Karamarkovic A (Zvezdara University Medical Center, Belgrade).

**Slovakia:** Panyko A (Univerzitná nemocnica Bratislava Ružinov).

**Spain:** Sanz Ortega G (Hospital Clínico San Carlos); De Andrés-Asenjo B (Hospital Clínico Universitario Valladolid); Nevado García C (Hospital Universitario 12 de Octubre); García Flórez LJ (Hospital Universitario Central de Asturias); Segura-Sampedro JJ (Hospital Universitario Son Espases); Colás-Ruiz E (Manacor Hospital); Blas Laina JL (Royo Villanova Hospital); Ponchietti L (San Jorge Hospital).

**Sweden:** Buchwald P (Skåne University Hospital, SUS, Malmö).

**Switzerland:** Gialamas E (Hôpital neuchâtelois).

**Turkey:** Ozben V (Acıbadem Mehmet Ali Aydınlar Üniversitesi Atakent Hastanesi); Rencuzogullari A (Adana Çukurova Üniversitesi Tıp Fakültesi Balcalı Hastanesi); Gecim İE (Ankara Üniversitesi Tıp Fakültesi); Altinel Y (Bagcilar research and training hospital, health sciences university); Isik O (Bursa Uludag University Hospital of Faculty of Medicine); Yoldas T (Ege University Medical Faculty Hospital); Isik A (Erzincan Üniversitesi Mengücek Gazi Eğitim ve Araştırma Hastanesi); Leventoğlu S (Gazi Universitesi Hastanesi); Ertürk MS (İstanbul Üniversitesi- Cerrahpaşa Tıp Fakültesi); Guner A (Karadeniz Teknik Universitesi Tıp Fakültesi Hastanesi); Güler SA (Kocaeli University Teaching Hospital); Attaallah W (Marmara University Research And Education Hospital); Ugur M (Mustafa Kemal University Research And Practice Hospital); Özbalcı GS (Ondokuz Mayıs Üniversitesi Tıp Fakültesi Hastanesi).

**United Kingdom:** Marzook H (Burton Queen's Hospital ); Eardley N (Countess of Chester Hospital); Smolarek S (Derriford Hospital); Morgan R (Glan Clwyd Hospital, Rhyl); Roxburgh C (Glasgow Royal Infirmary); Lala AK (Gwynedd Hospital); Salama Y (Kettering General Hospital); Singh B (Leicester General Hospital); Khanna A (Milton Keynes University Hospital); Evans M (Morriston Hospital); Shaikh I (Norfolk and Norwich University Hospital); Maradi Thippeswamy K (Prince Charles Hospital, Merthyr); Appleton B (Princess of Wales, Bridgend); Moug S (Royal Alexandra Hospital, Paisley); Smith I (Royal Cornwall Hospital ); Smart N (Royal Devon and Exeter Hospital); Shah P (Royal Glamorgan Hospital); Williams G (Royal Gwent Hospital); Khera G (Royal Sussex County Hospital); Goede A (Stoke Mandeville Hospital); Varcada M (The Royal Free Hospital); Parmar C (The Whittington Hospital); Duff S (University Hospital of South Manchester); Hargest R (University Hospital of Wales); Marriott P (Warwick Hospital); Speake D (Western General Hospital, Edinburgh); Ben Sassi A (Wrexham Maelor Hospital); Goede A (Wycombe General Hospital).

COLLABORATORS

**Australia:** Furfaro B, Daudu D, Golijanin N, Yek WY (Fiona Stanley Hospital); Capasso G, Mansour LT, Niu N, Seow W (Royal Adelaide Hospital).

**Bosnia & Herzegovina:** Hamidovic A, Kulovic E, Letic E, Salibasic M (Clinical Center University of Sarajevo); Aljić A, Letic E, Helez M (General Hospital Abdulah Nakas).

**Bulgaria:** Banji-Kelan A, Dimitrova N, Kavradjieva P, Ivanov V (Kaspela University Hospital); Jukaku A, Hadzhiev D, Mughal H (St. George Hospital); Slavchev M (University Hospital Eurohospital); Gabarski A, Karamanliev M, Vladova P, Iliev S, Yotsov T (University Hospital of Medical University - Pleven, Department of Surgical Oncology).

**Cyprus:** Ευσταθίου Η, Vetsa K, Gouvas N, Stavrinidou O, Papatheodorou P, Liassides T, Georgiou T (Nicosia General Hospital).

**Czech Republic:** Hegazi A, Al Nassrallah M, Altaf R, Amjad T, Negametzyanov M, Dušek T (Fakultní Nemocnice Hradec Králové); Zagibová D, Foltys F, Štefanová H (Motol University Hospital).

**Denmark:** Kjaer MD (Odense Universitetshospital); Mark-Christensen A (Svendborg Sygehus).

**Greece:** Paspala A, Papakonstantinou D, Bompetsi G, Sidiropoulos T (Attikon University Hospital); Sotiropoulos GC, Machairas N, Stamopoulos P (Evgenideio Hospital); Triantafyllou A, Theodoropoulos C, Kimpizi A, Theodorou D, Triantafyllou T, Palyvou T (Hippocration General Hospital Of Athens, National And Kapodistrian University Of Athens); Charalabopoulos A, Syllaios A, Schizas D, Liatsou E, Baili E, Vagios I, Tomara N, Davakis S (Laiko General Hospital); Balalis D (Saint Savvas Anticancer Hospital).

**Italy:** Palumbo A, Castaldi A, Foroni F (AORN A. Cardarelli); Picciariello A, Altomare DF, Dibra R, Papagni V (AOUC Policlinico di Bari); Urbani A, Rossin E (Arcispedale Sant'Anna, Università degli studi di Ferrara); Spolverato G, Nezi G, Romano P (Azienda Ospedaliera di Padova); Amendola A, Esposito E, Manigrasso M, Anoldo P, Vertaldi S (Azienda Ospedaliera Universitaria Federico II, Napoli); Gecchele G, Turri G, Sabrina ZS (Azienda Ospedaliera Universitaria Integrata Verona); Guerci C, Cammarata F, Lamperti GMB, Zaffaroni G, Benuzzi L, Ferrario L, Cigognini M (Azienda Ospedaliera Universitaria L. Sacco, Milano); Mazzeo C, Badessi G, Pintabona G (Azienda Ospedaliera Universitaria Policlinico 'G. Martino', Messina); Fassari A, Mingoli A, Cirillo B, D’Alterio C, Brachini G, Tancredi M, Zambon M, Aulicino M, Sapienza P, Lapolla P, Liberatore P, Bini S (Azienda Ospedaliera Universitaria Policlinico Umberto I, Roma); Scanu AM, Feo CF, Perra T (Azienda Ospedaliero Universitaria di Sassari); Iacomino A, Massani M, Pelizzo P, Tutino R, Rossi S, Vigna SA, Grossi U, Grillo V ("Ca Foncello" hospital); Agnes A, Schena CA, Belia F, Marincola G (Fondazione Policlinico Universitario A. Gemelli, Roma); Oddi A, Perotti B, Coletta D, Mario V, Perri P, Zazza S (IRCCS Regina Elena National Cancer Institute); Aversano A, Scala D, Di Lauro K, Leongito M, Piccirillo M, Patrone R (Istituto Nazionale per lo Studio e la Cura dei Tumori Fondazione Giovanni Pascale IRCCS, Napoli); Restini E, Cianci P, Capuzzolo S (Lorenzo Bonomo Hospital); Vignotto C, Pirozzolo G, Bao QR (Mestre hospital); Giuseppe C, Angarano E, Di Lena M ("Monsignor Dimiccoli" Hospital); Marino F, Perrone F, Pezzolla F, Gigante G (National Institute of Gastroenterology Research Hospital); Magistro C, Crippa J, Maspero M, Carnevali P (Niguarda Hospital, Milan); Lovisetto F, Trapani R, Zonta S (Ospedale Castelli, Verbania); Agostinelli L, Vittori L, Romeo L (Ospedale Ceccarini, Riccione); Doria E, Farnesi F, Danna R (Ospedale Civile Edoardo Agnelli, Pinerolo); Ferrara F (Ospedale San Carlo Borromeo, Milano); Biancafarina A, Andolfi E, Pellicano’ GA, Angelini M, Scricciolo M (Ospedale San Donato, Arezzo); Zanframundo C, Ciulli C, Ripamonti L, Cigagna L, Oldani M, Tamini N (Ospedale San Gerardo, Monza); Larcinese A, Rossi D, Picone E, Crescentini G (Ospedale San Giovanni Calibita Fatebenefralli, Roma); Tuminello F (Ospedale San Paolo, Savona); Caristo G (Ospedale Santa Corona, Pietra Ligure); Marano A, Sasia D, Migliore M, Giuffrida MC, Palagi S, Testa V (Ospedale Santa Croce e Carle, Cuneo); Borrello A, Lucarini A, Garofalo E, Canali G, Bragaglia L, Orlandi P (Ospedale Sant'Andrea, Roma); de Manzoni Garberini A, Nervegna F (Ospedale 'Spirito Santo' di Pescara); Marchegiani F, Damoli I (Policlinic Hospital Abano Terme - Department of Surgical Oncology, Robotics and New Technologies); Licata A, Trovato C, Cassaro F, Alicata F, Sardo F, Milazzo M (Policlinico 'Gaspare Rodolico', Catania); Randisi B, Dominici DM, Cocorullo G, Venturelli P (Policlinico 'P. Giaccone', Palermo); Gori A, Sartarelli L, Zanni M (Policlinico Sant'Orsola-Malpighi, Bologna); Pisanu A, Soddu C, Delogu D, Erdas E, Campus F, Cappellacci F, Casti F, Esposito G, Marcialis J, Atzeni J, Podda MG (Policlinico Universitario di Monserrato 'Duilio Casula'); Sensi B, Sica G, Franceschilli M, Campanelli M, Bellato V (Polyclinic Tor Vergata); Cannavera A, Putzu G, Cillara N (PO SS. Trinità, Cagliari); di Mola FF, Ricciardiello M (Private hospital Pierangeli); Sagnotta A, Picardi B, Solinas L, Loponte M, Rossi del Monte S, Rossi S (San Filippo Neri Hospital, Rome); Di Martino C, Linari C, Spagni G, Capezzuoli L, Tirloni L, Nelli T (San Giovanni di Dio Hospital); Caridi A, Elter C, Camassa M, D'Amico S, Bargellini T (San Giuseppe, Empoli); Cappiello A, Bianco F, Incollingo P (San Leonardo Hospital, ASL-NA3 Sud Castellammare di Stabia-Naples); Pinotti E, Montuori M (San Pietro Hospital); Maffione F, Romano L, Valiyeva S (San Salvatore Hospital); Spoletini D, Lisi G, Carlini M (Sant'Eugenio Hospital); Menegon Tasselli F, Pellino G, Bagaglini G, Sciaudone G, Selvaggi L, Menna MP (Università degli Studi della Campania 'Luigi Vanvitelli', Napoli); De Paola G, Sammarco G, Fulginiti S (U.O. di Chirurgia Generale, Policlinico Universitario di Catanzaro).

**Latvia:** Truskovs A, Weiß C, Saknītis G, Rauscher JTR, Larnovskis J, Jeyarajan-Davidsson M (Paula Stradiņa Klīniskā universitātes slimnīca); Malašonoks A, Nitisa D, Machatschek MJ, Gille N, Reiser SC (Rīgas Austrumu klīniskā universitātes slimnīca).

**Malta:** Farrugia M, Roshan MHK, Andrejevic P (Sptar Mater Dei).

**Netherlands:** Leseman C, Meima - van Praag EM, Tanis P (Academic Medical Centre); van de Ven A, Meima - van Praag EM, Chen J (Flevoziekenhuis); van Dalen AS, Top C, Gerhards M, Detering R (Onze Lieve Vrouwe Gasthuis (OLVG) locatie oost).

**Portugal:** Matos C, Monteiro C, Silva C, Pinto D, Mendes J, Couto J, Leite M (Hospital de Santa Luzia); Velez C, Damasio Cotovio M, Cinza AM, Pereira M, Pedroso de Lima R (Hospital Espírito Santo Évora, E.P.E.); Botelho P (Hospital Garcia de Orta).

**Republic of Ireland:** Quigley A, Boyle E, Yang HW, Banerjee I, Rahmat S, Afzal Z (Adelaide and Meath Hospital, Tallaght); O'Neill A, Reid C, Dumitrascu F, Croyle JA, Gressmann K, Cullen N (Beaumont Hospital, Dublin); Graham A, Nasehi A, Montano King C, Gaule L (Mayo University Hospital); Martin B, Stokell C, Crone L, Sanderson N, Farnan R, jassim S (St James's Hospital, Dublin); Arnold A, Chan B, Chua Vi Long K, Kaka N, Pandey S, Neo WX (University Hospital Galway).

**Romania:** Chitul A, Bezede C, Grama F (Coltea Clinical Hospital); Beuca A, Cincilei D, David A, Blaga M, Blaga SN, Fagarasan V (County Emergency Hospital Cluj-Napoca).

**Russia:** Tulina I, Khetagurova M, Rodimov S (Clinic of Colorectal and Minimally Invasive Surgery - Sechenov University); Kapustina A, Mekhralyzade A, Zabiyaka M (Immanuel Kant Baltic Federal University, Kaliningrad Regional Clinical Hospital).

**Serbia:** Juloski J, Janković U, Cuk V (Zvezdara University Medical Center, Belgrade).

**Slovakia:** Panyko A, Hájska M, Dubovský M, Hrošová M, Ferancikova N (Univerzitná nemocnica Bratislava Ružinov).

**Spain:** Camarero Rodríguez E, Laguna Alcántara F, Adarraga J (Hospital Clínico San Carlos); Jezieniecki C, Ruiz Soriano M, Gómez Sanz T (Hospital Clínico Universitario Valladolid); Suarez A, Sánchez García C, Marín Santos JM (Hospital Universitario 12 de Octubre); Alonso Batanero E, Cifrian Canales I, Llosa Pérez J, Merayo M (Hospital Universitario Central de Asturias); Urbieta A, Gegúndez Simón A, Tone JF, Gazo Martínez J, Vicario Bravo M, Chavarrias N (Hospital Universitario La Paz); Gil Catalán A, Oseira A, Villalonga B, Soldevila Verdeguer C, Jeri S (Hospital Universitario Son Espases); Colás-Ruiz E, Perez Calvo J (Manacor Hospital); Nogués A, Cros B, Yánez C, Talal El-Abur I, Blas Laina JL (Royo Villanova Hospital); Utrilla Fornals A, Roldón Golet M, García Domínguez M, Colsa P, Gimenez Maurel T (San Jorge Hospital).

**Sweden:** Delorme M, Buchwald P, Axmarker T (Skåne University Hospital, SUS, Malmö).

**Switzerland:** Gialamas E, Chevallay M, Pham TV (Hôpital neuchâtelois).

**Turkey:** Ozmen BB, Sel EK, Ozben V (Acıbadem Mehmet Ali Aydınlar Üniversitesi Atakent Hastanesi); Atar C, Aktas MK, Aba M (Adana Çukurova Üniversitesi Tıp Fakültesi Balcalı Hastanesi); Ozkan BB, Sarkin M, Akkaya YM (Ankara Üniversitesi Tıp Fakültesi); Durmaz AG, Calikoglu F, Gullu HF (Bagcilar research and training hospital, health sciences university); Boğa A, Aktaş A, Bakar B, Demirel MT, Kural S, Hysejni X (Bursa Uludag University Hospital of Faculty of Medicine); Zafer F, Taser M, Guzel OR, Bozbiyik O (Ege University Medical Faculty Hospital); Isik A, Özen D, Ölmez M, Kaya Y (Erzincan Üniversitesi Mengücek Gazi Eğitim ve Araştırma Hastanesi); Uyar B, Gülçek E, Kayacan GS, Atıcı N, Gul OF, Altiner S (Gazi Universitesi Hastanesi); Ibis B, Altunsu S, Banaz T (İstanbul Üniversitesi- Cerrahpaşa Tıp Fakültesi); Diler C, Demirbas I, Usta MA, Erkul O, Orman R, Salih S (Karadeniz Teknik Universitesi Tıp Fakültesi Hastanesi); Utkan NZ, Tatar OC, Güler SA (Kocaeli University Teaching Hospital); Acil C, Ozgur E, Maddahali M (Marmara University Research And Education Hospital); Turhan AB, Eskici AB, Ular B, Doğru M, Öztürk OU, Arslan ER (Mustafa Kemal University Research And Practice Hospital); Panahi Sharif A, Hurmuzlu D, Dikmen E, Ates J, Bircan R, Cavus T (Ondokuz Mayıs Üniversitesi Tıp Fakültesi Hastanesi); Sever AE, Balak B, Duman E, Korkmaz K, Altay L, Emanet O (Yeditepe Universitesi Hastanesi).

**United Kingdom**: Cullen F, Tan JY, Sharma P (Aberdeen Royal Infirmary); Nathan A, Rottenberg A, Williams CY, Mitrofan CG, Xu D, Bawa JH, Morris P, Troller R (Addenbrooke's Hospital); Gordon D, Richmond G, Hui JC, Hagan N, Ighomereho O, Rocks R, McCabe S (Altnagelvin Area Hospital); Fitzpatrick A, Mooney J, Nicoletti J, Hui JC, Auterson L, Darrah N, Soh VWY (Antrim Area Hospital); Light A, Ong CS, Utukuri M (Bedford Hospital); Gallagher C, Stuart LM, Hipolito M, Douglas N, Ghazal R (Belfast City Hospital); Parris G, Catchpole J, Tansey M, Bryden M, Jamal S, Karim Z (Blackpool Victoria Hospital); Lyon-Dean C, Bhojwani D, Rowley G, Lee KS, Whitehurst O (Bristol Royal Infirmary); Mirza A, Sheikh F, Yousaf H, Bilbao J, Sinclair R, Takar S (Burton Queen's Hospital ); Kressel H, McGing RM, Chan V (Castle Hill Hospital); Mallon A, Schack K, Osborne R, Baldemor S, Smyth S, Gilmour S (Causeway Hospital); Ting A, Bozonelou I, Saunders P, Qhaireel Anwar QA, Tirimanna R, Jauhari S (Chelsea and Westminster Hospital); Gardener A, Walker B, Spilsbury C, Wenban C, Reddy H, Conway-Jones R, Loganathan S (Churchill Hospital); Clynch A, James C, Matey E, Cameron F, James R, Roberts W (Countess of Chester Hospital); Gicquel A, Milliken C, Forbes J, Rubinchik P, O’Brien S (Craigavon Area Hospital); Isaac A, Azmi A, Hawkes C, Cornett L, Adarkwah P, McConville R, O'Hara S (Daisy Hill Hospital); Tijare C, Parkes J, Yao L, Ahmad R, Balasubramanya S, Shafiq U (Derriford Hospital); Mhaisalkar A, Gurung A, Sadik H, de Stadler K, Elias S, Thomas T (Ealing Hospital); Madras A, Jani A, Daler HK, Tong KS, Sundaralingam SS, Nowinka Z (Furness general hospital); Szal A, Khan A, O'Sullivan C, Baker E, Joseph-Gubral J, Gala T (Glan Clwyd Hospital, Rhyl); Chen JY (Glasgow Royal Infirmary); Turner B, Hadley E, Trivedi R (Gloucestershire Royal Hospital); Igwelaezoh E, Goh E, Barton H, Allison W, Hurst W (Great Western Hospital, Swindon); Alam F, Parkes I, Hassan K, Jamshaid M, Azizan N, Burgher T (Gwynedd Hospital); Afzal A, Eltilib I, Zahid M, Sadiq O (Huddersfield Royal Infirmary); Lloyd A, Mason P, Ho R (Hull Royal Infirmary ); Brazukas A, Li CH, Kamdar M, Mohamed Nazeer MN, Tzoumas N (James Cook University Hospital); Mighiu A, Kim D, Wilkins L, Kuo L, Conway-Jones R, Rafe T, Noton T (John Radcliffe Hospital); Maduka D, Cheema H, Farag K, Mirza M, Abdellatif M, Nzewi R (Kettering General Hospital); Kruczynska A, Grasselli H, Yousuff M, Ahmed N, Bassi R (King's Mill Hospital); Mann AK, Chopra J, Shaikh M, Sharma P, D Sa S, Tsimplis V (Leicester General Hospital); Ghanchi A, Skene E, Asim K, Zaheer M, Chan S (Leicester Royal Infirmary); Dalton H, Gibbons K, Adderley O (Manchester Royal Infirmary); Chukwujindu I, Jayasuriya I, Sivanu K, Borumand M, Bylapudi SK (Milton Keynes University Hospital); Chick G, Bridges I, Tomlin J, McKenna J, Nandra N, Grace N (Morriston Hospital); Grieco C, Quek FF, Mercer R, Latif S, Brankin-Frisby T (Musgrove Park Hospital); Sattar A, Aslam A, Edelsten E, Shafi S, Kouli T, Ford V (Ninewells Hospital); Gurung F, Kiam JS, Fernandes M, Deader N, Ponniah R, Jamieson S (Norfolk and Norwich University Hospital); Davies A, Taubwurcel J, Aung MT, Desai R, Begum S, Jamadar T (Northampton District General Hospital); Kangatharan A, Rzeszowski B, Ho C, Yap SHK, Prendergast M, Sethi R (Northwick Park/St. Mark's Hospitals); Duku A, Lowe C, Bray J, Elsamani K, Ghobrial M, Nichita V (Peterborough City Hospital); Wagstaff A, Hughes C (Prince Charles Hospital, Merthyr); Rengasamy E, Abu Hassan F, Mahmood H, Savill N, Shah S, Almeida T (Princess of Wales, Bridgend); Sinan LOH (Queen Elizabeth University Hospital, Glasgow); Edwards A, Antypas A, Catchpole B, El-Dalil D, Halford Z (Queens Medical Centre, Nottingham); Carmichael A, Khoo EJM, Alsusa H (Royal Albert Edward Infirmary (Wigan Infirmary)); Salim EE, Boyd M (Royal Alexandra Hospital, Paisley); Reid C, Stark D, Williams J, Feyi-Waboso J, Patel M, Zeidan Z (Royal Cornwall Hospital ); Bailey E, Bapty J, Brazkiewicz M, Minhas N (Royal Derby Hospital); Tremlett A, Fowler G, Pringle H, Mankal S, Kaminskaite V, Chung W (Royal Devon and Exeter Hospital); Rees E, Parry-Jones E, Anderson K (Royal Glamorgan Hospital); Mcforrester A, Stanley A, Hoather A, Wise H, Laid I, Giudiceandrea I, Scriven J (Royal Gwent Hospital); Braniste A, Wilson A, Le Blevec L, Pakunwanich N, Evans N, Chong HL (Royal Lancaster Hospital); White C, Hunter J, Haque M, Vanalia P, Murdoch S, Choudhary T (Royal Preston Hospital); McCann A, Harun A, Shah H, Dieseru N, Hunt S, Shafiq Y (Royal Stoke University Hospital); Murphy A, Bickley-Morris E, Emms L, Dare M, Patel M, Akula Y (Royal Sussex County Hospital); Yates C, Deliyannis E, Mayes F, Ellacott M, Zagorac Z (Royal United Hospital); Farren A, Manning C, Hughed C, Stewart EG, Lim KH, Chohan N (Royal Victoria Hospital); Thaker A, Thompson B, Ziolkowska K (Royal Victoria Infirmary, Newcastle); Ahari D, Burdekin E, Okwu U (Salford Royal Hospital); Akintunde A, Lhaf F, Khoda F, Douthwaite J, Govindan R, Leelamanthep S (Scunthorpe General Hospital ); Gull E, Wright F, Dundas L, Okocha M, Mackdermott N, Burchi-Khairy T (Southmead Hospital, Bristol); Campbell I, Walsh J, Yeo JY, Meehan S (South West Acute Hospital (SWAH)); Banerjee D, Fu M, Kawka M, Ali T, Hussain Z (St. Mary's Hospital, London); Thomas C, Ahmad H, Moroney J, Yick C, Risquet R (Stoke Mandeville Hospital); Ntuiabane D, Shimato M, Khan M, Ilangovan S (The Royal Free Hospital); Vaselli NM, Smithers R, Uhanowita Marage R (The Royal Liverpool University Hospital); Valnarov-Boulter A, Kayran J, Banerjee M, Parekh-Hill N (The Whittington Hospital); Hooper A, Bowen J, Jagdish R (Torbay Hospital); Mcquoid C, Khan N, O Hare R, Jeffery S (Ulster Hospital); Devine A, Zahid A, Elsworth C, Walter L, Dhillon S, Rao S (University Hospital of South Manchester); Anthony A, Ashaye A, Phillips N, Faderani R, Pengelly S, Choi S, Kwak SY, Lau YHL (University Hospital of Wales); Bagheri K, Pancharatnam R, McDonnell S (Warwick Hospital); Ong DYC, Kerr E, Falconer K, Clancy N, Douglas S, Zhang Y (Western General Hospital, Edinburgh); Greenfield F, Mutanga I, McAlinden J, Olivier J, Willis L (Weston General Hospital); Adefolaju A, Agarwal H, Barter R (Whiston Hospital); Harris G, Spencer G, Lim HJ, Lee MW, V Vadiveloo T (Wrexham Maelor Hospital); Herbert G, Moroney J, Yick C, Patel R, Risquet R (Wycombe General Hospital); Shah M, Slim N, El Falaha S (Yeovil District Hospital); Wong C, Soare C, Akram J, Elsayeh K, Bozhkova L, Ma Y (York Hospital).

VALIDATORS

**Australia:** Vo UG (Fiona Stanley Hospital); Tan HWN (Royal Adelaide Hospital).

**Bosnia & Herzegovina:** Leto L (General Hospital Abdulah Nakas).

**Bulgaria:** Kamal MA (Kaspela University Hospital); Hadzhieva E (St. George Hospital); Krastev P (University Hospital Eurohospital); Tonchev P (University Hospital of Medical University - Pleven, Department of Surgical Oncology).

**Cyprus:** Kokkinos G, Pozotou I (Nicosia General Hospital).

**Czech Republic:** Sabbagh D (Fakultní Nemocnice Hradec Králové); Votava J, Kocián P (Motol University Hospital).

**Greece:** St F, Koliakos N (Attikon University Hospital); Tsaparas P (Evgenideio Hospital); Zografos G (Hippocration General Hospital Of Athens, National And Kapodistrian University Of Athens); Mantas D, Tsourouflis G (Laiko General Hospital); Fradelos E (Saint Savvas Anticancer Hospital).

**Italy:** Castaldi A (AORN A. Cardarelli); Trigiante G, Labellarte G (AOUC Policlinico di Bari); Resta G (Arcispedale Sant'Anna, Università degli studi di Ferrara); Capelli G (Azienda Ospedaliera di Padova); D'Amore A, Verlingieri V (Azienda Ospedaliera Universitaria Federico II, Napoli); Campagnaro T (Azienda Ospedaliera Universitaria Integrata Verona); Maffioli A (Azienda Ospedaliera Universitaria L. Sacco, Milano); Viscosi F (Azienda Ospedaliera Universitaria Policlinico 'G. Martino', Messina); De Lucia C, Poillucci G, Meneghini S (Azienda Ospedaliera Universitaria Policlinico Umberto I, Roma); Fancellu A (Azienda Ospedaliero Universitaria di Sassari); Colella M ("Ca Foncello" hospital); Biondi A (Fondazione Policlinico Universitario A. Gemelli, Roma); De Peppo V (IRCCS Regina Elena National Cancer Institute); Pace U, Albino V (Istituto Nazionale per lo Studio e la Cura dei Tumori Fondazione Giovanni Pascale IRCCS, Napoli); Gattulli D (Lorenzo Bonomo Hospital); Piangerelli A (Mestre hospital); Kalivaci D ("Monsignor Dimiccoli" Hospital); Sisto G (National Institute of Gastroenterology Research Hospital); Mazzola M (Niguarda Hospital, Milan); Caneparo A (Ospedale Castelli, Verbania); Grassia M (Ospedale Ceccarini, Riccione); Lunghi EG (Ospedale Civile Edoardo Agnelli, Pinerolo); Andolfi E (Ospedale San Donato, Arezzo); Nespoli LC, Angrisani M (Ospedale San Gerardo, Monza); Sinibaldi G (Ospedale San Giovanni Calibita Fatebenefralli, Roma); Langone A (Ospedale San Paolo, Savona); Galleano R (Ospedale Santa Corona, Pietra Ligure); Gelarda E (Ospedale Santa Croce e Carle, Cuneo); Virgilio E (Ospedale Sant'Andrea, Roma); Angelini E (Ospedale 'Spirito Santo' di Pescara); Fornasier C (Policlinic Hospital Abano Terme - Department of Surgical Oncology, Robotics and New Technologies); Asero S (Policlinico 'Gaspare Rodolico', Catania); Venturelli P (Policlinico 'P. Giaccone', Palermo); Filippone E (Policlinico Sant'Orsola-Malpighi, Bologna); Frongia F, Calò PG (Policlinico Universitario di Monserrato 'Duilio Casula'); Bellato V (Polyclinic Tor Vergata); Panaccio P (Private hospital Pierangeli); Sagnotta A, Loponte M (San Filippo Neri Hospital, Rome); Ipponi P (San Giovanni di Dio Hospital); D'Amico S (San Giuseppe, Empoli); Gili S (San Leonardo Hospital,ASL-NA3 Sud Castellammare di Stabia-Naples); Giuliani A (San Salvatore Hospital); Lisi G (Sant'Eugenio Hospital); Braccio B (Università degli Studi della Campania 'Luigi Vanvitelli', Napoli); Tiesi V (U.O. di Chirurgia Generale, Policlinico Universitario di Catanzaro).

**Latvia:** Stolcers K, Kokaine L (Paula Stradiņa Klīniskā universitātes slimnīca); Novikovs V (Rīgas Austrumu klīniskā universitātes slimnīca).

**Malta:** Farrugia M (Sptar Mater Dei).

**Netherlands:** Capel L (Academic Medical Centre); Bastiaenen V (Flevoziekenhuis); Heijmans H (Onze Lieve Vrouwe Gasthuis (OLVG) locatie oost).

**Portugal:** Ribeiro da Silva B (Hospital de Santa Luzia); Silva A (Hospital Espírito Santo Évora, E.P.E.); Botelho P, Henriques S (Hospital Garcia de Orta).

**Republic of Ireland:** Gan SZ (Adelaide and Meath Hospital, Tallaght); Ramanayake H, Nolan M (Beaumont Hospital, Dublin); Kakodkar P (Mayo University Hospital); Temperley H (St James's Hospital, Dublin); Kakodkar P (University Hospital Galway).

**Romania:** Ciofic E (Coltea Clinical Hospital); Beuca A, Pop BA (County Emergency Hospital Cluj-Napoca).

**Russia:** Kurtenkov M (Immanuel Kant Baltic Federal University, Kaliningrad Regional Clinical Hospital).

**Serbia:** Jovanović M (Zvezdara University Medical Center, Belgrade).

**Slovakia:** Vician M (Univerzitná nemocnica Bratislava Ružinov).

**Spain:** Egea Arias P (Hospital Clínico San Carlos); Beltrán de Heredia J (Hospital Clínico Universitario Valladolid); Labalde Martinez M (Hospital Universitario 12 de Octubre); De Santiago Alvarez I (Hospital Universitario Central de Asturias); Alvarez-Gallego M (Hospital Universitario La Paz); Colás-Ruiz E (Manacor Hospital); Talal El-Abur I (Royo Villanova Hospital); Rodriguez Artigas JM (San Jorge Hospital).

**Sweden:** Buchwald P (Skåne University Hospital, SUS, Malmö).

**Switzerland:** Dwidar O (Hôpital neuchâtelois).

**Turkey:** Korkmaz HK (Acıbadem Mehmet Ali Aydınlar Üniversitesi Atakent Hastanesi); Eray IC (Adana Çukurova Üniversitesi Tıp Fakültesi Balcalı Hastanesi); Meriç S (Bagcilar research and training hospital, health sciences university); Aydin R (Bursa Uludag University Hospital of Faculty of Medicine); Çetin B (Ege University Medical Faculty Hospital); Özen D (Erzincan Üniversitesi Mengücek Gazi Eğitim ve Araştırma Hastanesi); Yalcinkaya A, Karaca BE (Gazi Universitesi Hastanesi); Kuyumcu OF (İstanbul Üniversitesi- Cerrahpaşa Tıp Fakültesi); Baki BE (Karadeniz Teknik Universitesi Tıp Fakültesi Hastanesi); Yüksel E (Kocaeli University Teaching Hospital); Uprak TK (Marmara University Research And Education Hospital); Ugur M (Mustafa Kemal University Research And Practice Hospital); Karabulut K (Ondokuz Mayıs Üniversitesi Tıp Fakültesi Hastanesi); Kavukçu E (Yeditepe Universitesi Hastanesi).

**United Kingdom:** Mansor A (Aberdeen Royal Infirmary); Troller R (Addenbrooke's Hospital); Hackett R (Altnagelvin Area Hospital); Zammit-Maempel M (Antrim Area Hospital); Sabaratnam R (Bedford Hospital); Nicoletti J (Belfast City Hospital); Maan A (Blackpool Victoria Hospital); Ferarrio I, Dixon L (Bristol Royal Infirmary); Halai H (Burton Queen's Hospital ); Sethi S (Castle Hill Hospital); Nelson L (Causeway Hospital); Grassam-rowe A (Churchill Hospital); Krishnan E (Countess of Chester Hospital); Deeny D (Craigavon Area Hospital); McKeever M (Daisy Hill Hospital); George Pandeth A, Dhavala P (Derriford Hospital); Sreenivasan S (Furness general hospital); Sundaram Venkatesan G (Glan Clwyd Hospital, Rhyl); Zhu L (Glasgow Royal Infirmary); Atiyah Z (Gloucestershire Royal Hospital); Gregory J, Morey T (Great Western Hospital, Swindon); Seymour Z (Gwynedd Hospital); Holdsworth L (Hull Royal Infirmary ); Abdelmahmoud S (James Cook University Hospital); Bourhill J (John Radcliffe Hospital); Bisheet G (Kettering General Hospital); Shaw J (King's Mill Hospital); Kulkarni K (Leicester General Hospital); Kumarakulasingam P (Leicester Royal Infirmary); Pillay S (Manchester Royal Infirmary); Al-Habsi R (Milton Keynes University Hospital); Kungwengwe G (Morriston Hospital); Richards J, Davoudi K (Musgrove Park Hospital); Ibrahim B (Ninewells Hospital); Tailor B (Norfolk and Norwich University Hospital); Zayed M (Northampton District General Hospital); Chen F (Peterborough City Hospital); Bailey S (Prince Charles Hospital, Merthyr); Sheefat S (Princess of Wales, Bridgend); Nawaz G (Queen Elizabeth University Hospital, Glasgow); Pawar R (Queens Medical Centre, Nottingham); Marsh S (Royal Albert Edward Infirmary (Wigan Infirmary)); Sam ZH (Royal Alexandra Hospital, Paisley); Roy Bentley S (Royal Cornwall Hospital ); Simpson C (Royal Derby Hospital); Hughes J (Royal Devon and Exeter Hospital); Lim Y (Royal Glamorgan Hospital); Ooi R (Royal Gwent Hospital); Toh WH (Royal Lancaster Hospital); Mannion P (Royal Preston Hospital); Lovett A (Royal Stoke University Hospital); Kinčius A, Hussein S (Royal Sussex County Hospital); Kirby E, Beckett RG (Royal United Hospital); Salmon J (Royal Victoria Hospital); Rafie A (Salford Royal Hospital); Glynn T (Scunthorpe General Hospital ); Choo SY, Lyons S (Southmead Hospital, Bristol); Browne D (South West Acute Hospital (SWAH)); Ravindran W (St. Mary's Hospital, London); Ahmad S (Stoke Mandeville Hospital); Erotocritou M (The Royal Free Hospital); Zhu X (The Royal Liverpool University Hospital); Erotocritou M (The Whittington Hospital); Bradbury M (Torbay Hospital); McNulty J (Ulster Hospital); McCarthy L (University Hospital of South Manchester); Ng J (University Hospital of Wales); Karmally Z (Warwick Hospital); McTeir K (Western General Hospital, Edinburgh); Hanna M (Weston General Hospital); Tan E (Wrexham Maelor Hospital); Namdeo S (Wycombe General Hospital); Schembri R (Yeovil District Hospital); Pusey E (York Hospital).

Table S1: Primary operation performed

| Primary operation performed, n (%) | |  |
| --- | --- | --- |
| Colon resection | |  |
|  | Extended right hemicolectomy | 40 (2.2) |
|  | Ileocolic resection | 58 (3.2) |
|  | Left hemicolectomy | 104 (5.8) |
|  | Right hemicolectomy | 433 (24.0) |
|  | Sigmoid colectomy (including Hartmann’s Procedure) | 204 (11.3) |
|  | Sub-total colectomy | 25 (1.4) |
|  | Total colectomy | 17 (0.9) |
|  | Transverse colectomy | 11 (0.6) |
| Rectum resection | |  |
|  | Abdominoperineal resection | 44 (2.4) |
|  | Anterior resection | 420 (23.3) |
|  | Completion proctectomy | 50 (2.8) |
|  | Pan-proctocolectomy | 23 (1.3) |
| Stoma formation/closure | |  |
|  | Formation of stoma (ileostomy or colostomy) | 106 (5.9) |
|  | Closure/reversal of stoma (ileostomy or colostomy) | 268 (14.8) |

Table S2: Underlying pathology

| Underlying pathology, n (%) | |  |
| --- | --- | --- |
| Benign | |  |
|  | Bowel ischaemia | 10 (0.6) |
|  | Diverticular disease | 124 (6.9) |
|  | Inflammatory bowel disease | 163 (9.0) |
|  | Large bowel obstruction | 19 (1.1) |
|  | Small bowel obstruction | 6 (0.3) |
|  | Other benign | 234 (13.0) |
| Malignancy | | 1248 (69.1) |

Table S3: Balance table for characteristics of patients before and after propensity-score matching on intraperitoneal drain insertion

|  |  | Intraperitoneal drain insertion | | | | | |
| --- | --- | --- | --- | --- | --- | --- | --- |
|  |  | Unmatched characteristics | | | Propensity-score matched | | |
|  |  | No (n=693) | Yes (n=802) | aSMD | No (n=693) | Yes (n=802) | aSMD |
| Age | <70 years | 385 (45.7) | 457 (54.3) | 0.029 | 397 (46.5) | 457 (53.5) | 0.006 |
|  | ≥70 years | 308 (47.2) | 345 (52.8) |  | 296 (46.2) | 345 (53.8) |  |
| Sex | Female | 317 (48.6) | 335 (51.4) | 0.08 | 240 (41.7) | 335 (58.3) | 0.147 |
|  | Male | 376 (44.6) | 467 (55.4) |  | 453 (49.2) | 467 (50.8) |  |
| BMI (kg/m^2^) | Underweight-Normal | 272 (46.5) | 313 (53.5) | 0.084 | 229 (42.3) | 313 (57.7) | 0.143 |
|  | Overweight | 259 (44.3) | 326 (55.7) |  | 328 (50.2) | 326 (49.8) |  |
|  | Obese | 162 (49.8) | 163 (50.2) |  | 136 (45.5) | 163 (54.5) |  |
| Smoking status | Never | 370 (45.6) | 441 (54.4) | 0.042 | 360 (44.9) | 441 (55.1) | 0.117 |
|  | Previous | 218 (47.9) | 237 (52.1) |  | 195 (45.1) | 237 (54.9) |  |
|  | Current* | 105 (45.9) | 124 (54.1) |  | 138 (52.7) | 124 (47.3) |  |
| ASA score | I-II | 493 (48.6) | 522 (51.4) | 0.13 | 500 (48.9) | 522 (51.1) | 0.153 |
|  | III-V | 200 (41.7) | 280 (58.3) |  | 193 (40.8) | 280 (59.2) |  |
| Diabetes Mellitus | No | 588 (46.9) | 666 (53.1) | 0.077 | 581 (46.6) | 666 (53.4) | 0.032 |
|  | non-IDDM | 88 (45.4) | 106 (54.6) |  | 90 (45.9) | 106 (54.1) |  |
|  | IDDM | 17 (36.2) | 30 (63.8) |  | 22 (42.3) | 30 (57.7) |  |
| Previous abdominal surgery | No | 337 (46.0) | 395 (54.0) | 0.012 | 367 (48.2) | 395 (51.8) | 0.074 |
|  | Yes | 356 (46.7) | 407 (53.3) |  | 326 (45.5) | 407 (55.5) |  |
| Anticoagulation therapy | No | 509 (43.7) | 656 (56.3) | 0.201 | 552 (45.7) | 656 (54.3) | 0.054 |
|  | Yes | 184 (55.8) | 146 (44.2) |  | 141 (49.1) | 146 (50.9) |  |
| Immunosuppression status | No | 615 (47.9) | 669 (52.1) | 0.154 | 563 (45.7) | 669 (54.3) | 0.057 |
|  | Yes | 78 (37.0) | 133 (63.0) |  | 130 (49.4) | 133 (50.6) |  |
| Underlying pathology | Benign | 234 (52.1) | 215 (47.9) | 0.152 | 183 (46.0) | 215 (54.0) | 0.009 |
|  | Malignancy | 459 (43.9) | 587 (56.1) |  | 510 (46.5) | 587 (53.5) |  |
| Type of surgery | colon | 389 (52.1) | 358 (47.9) | 0.639 | 306 (46.1) | 358 (53.9) | 0.046 |
|  | colon/rectum | 110 (24.6) | 338 (75.4) |  | 305 (47.4) | 338 (52.6) |  |
|  | stoma | 194 (64.7) | 106 (35.3) |  | 83 (43.9) | 106 (56.1) |  |
| Operative approach | Minimally invasive | 413 (53.2) | 364 (46.8) | 0.287 | 310 (46.0) | 364 (54.0) | 0.013 |
|  | Open | 280 (39.0) | 438 (61.0) |  | 383 (46.7) | 438 (53.3) |  |
| Perforated bowel | No | 681 (46.7) | 776 (53.3) | 0.097 | 675 (46.5) | 776 (53.5) | 0.038 |
|  | Yes | 12 (31.6) | 26 (68.4) |  | 18 (40.9) | 26 (59.1) |  |
| Operative contamination | Clean-Contaminated | 655 (47.4) | 728 (52.6) | 0.144 | 638 (46.7) | 728 (53.3) | 0.046 |
|  | Contaminated / Dirty | 38 (33.9) | 74 (66.1) |  | 55 (42.6) | 74 (57.4) |  |
| Operation duration (minutes) | (SD) | 171 (82.8) | 220 (103) | 0.527 | 220 (89.9) | 220 (103) | 0.001 |
| Intraoperative anastomosis | No | 177 (44.2) | 223 (55.8) | 0.051 | 171 (43.4) | 223 (56.6) | 0.071 |
|  | Yes | 516 (47.1) | 579 (52.9) |  | 522 (43.4) | 579 (52.6) |  |
| Intraoperative vascular or organ injury | No | 670 (47.2) | 750 (52.8) | 0.147 | 662 (46.9) | 750 (53.1) | 0.088 |
|  | Yes | 23 (30.7) | 52 (69.3) |  | 31 (37.3) | 52 (62.7) |  |
| Intraoperative blood transfusion | No | 684 (47.3) | 763 (52.7) | 0.207 | 680 (47.1) | 763 (52.9) | 0.166 |
|  | Yes | 9 (18.7) | 39 (81.3) |  | 13 (25.0) | 39 (75.0) |  |
| * Includes those who stopped smoking within 6 weeks  Data are percentages unless otherwise stated.  Abbreviations: aSMD: absolute Standardized Mean Difference; BMI: Body Mass Index; ASA: American Society of Anaesthesiologists; IDDM: Insulin-dependent Diabetes Mellitus; SD: Standard Deviation. | | | | | | | |

Table S4: Cox-proportional hazards regression model of factors associated with time to discharge (days)

|  |  | Discharge at 30 days | | Time to discharge (days) | |
| --- | --- | --- | --- | --- | --- |
|  |  | No (n=68) | Yes (n=1699) | Univariable HR (95% CI) | Multilevel HR (95% CI) |
| Intraperitoneal drain insertion | No | 27 (3.2) | 823 (96.8) | - | - |
|  | Prophylactic | 23 (3.7) | 596 (96.3) | 0.73 (0.66-0.82, p<0.001) | 0.82 (0.71-0.96, p=0.012) |
|  | Indication | 18 (6.0) | 280 (94.0) | 0.65 (0.57-0.75, p<0.001) | 0.86 (0.72-1.03, p=0.102) |
| Age (years) | Mean (SD) | 67.5 (16.6) | 63.9 (14.2) | 0.99 (0.99-1.00, p<0.001) | 0.99 (0.99-0.99, p<0.001) |
| Sex | Female | 32 (4.1) | 748 (95.9) | - | - |
|  | Male | 36 (3.6) | 953 (96.4) | 1.00 (0.91-1.11, p=0.939) | - |
| Smoking status | Never | 30 (3.5) | 822 (96.5) | - | - |
|  | Previous | 9 (1.9) | 465 (98.1) | 1.09 (0.97-1.22, p=0.145) | 1.17 (1.04-1.31, p=0.010) |
|  | Current* | 16 (6.8) | 219 (93.2) | 0.96 (0.83-1.12, p=0.613) | 0.95 (0.79-1.14, p=0.607) |
| BMI (kg/m^2^) | Underweight-Normal | 32 (4.7) | 643 (95.3) | - | - |
|  | Overweight | 20 (3.0) | 639 (97.0) | 1.17 (1.04-1.30, p=0.006) | 1.27 (1.13-1.43, p<0.001) |
|  | Obese | 14 (3.8) | 355 (96.2) | 1.06 (0.93-1.20, p=0.417) | 1.17 (1.00-1.36, p=0.048) |
| ASA score | I-II | 26 (2.2) | 1176 (97.8) | - | - |
|  | III-V | 42 (7.5) | 520 (92.5) | 0.73 (0.66-0.81, p<0.001) | 0.81 (0.72-0.92, p=0.001) |
| Previous abdominal surgery | No | 25 (2.9) | 835 (97.1) | - | - |
|  | Yes | 43 (4.7) | 865 (95.3) | 0.90 (0.82-0.99, p=0.029) | - |
| Cardiovascular disease | No | 52 (3.5) | 1422 (96.5) | - | - |
|  | Yes | 16 (5.4) | 279 (94.6) | 0.85 (0.74-0.96, p=0.012) | - |
| Diabetes Mellitus | No | 54 (3.6) | 1436 (96.4) | - | - |
|  | non-IDDM | 10 (4.5) | 211 (95.5) | 0.97 (0.84-1.13, p=0.718) | - |
|  | IDDM | 4 (7.8) | 47 (92.2) | 0.89 (0.66-1.19, p=0.419) | - |
| Immunosuppression status | No | 56 (3.7) | 1467 (96.3) | - | - |
|  | Yes | 10 (4.1) | 234 (95.9) | 0.83 (0.72-0.95, p=0.008) | 0.83 (0.70-0.97, p=0.023) |
| Anticoagulation therapy | No | 51 (3.7) | 1331 (96.3) | - | - |
|  | Yes | 17 (4.4) | 369 (95.6) | 0.87 (0.77-0.97, p=0.015) | - |
| Underlying pathology | Benign | 26 (4.8) | 520 (95.2) | - | - |
|  | Malignancy | 42 (3.4) | 1180 (96.6) | 1.00 (0.90-1.11, p=0.992) | - |
| Perforated bowel | No | 62 (3.6) | 1667 (96.4) | - | - |
|  | Yes | 6 (15.0) | 34 (85.0) | 0.56 (0.40-0.79, p=0.001) | 0.55 (0.40-0.77, p<0.001) |
| Operative contamination | Clean-Contaminated | 59 (3.6) | 1587 (96.4) | - | - |
|  | Contaminated / Dirty | 9 (7.5) | 111 (92.5) | 0.81 (0.67-0.99, p=0.036) | - |
| Operative approach | Minimally invasive | 25 (2.7) | 899 (97.3) | - | - |
|  | Open | 43 (5.1) | 801 (94.9) | 0.65 (0.59-0.72, p<0.001) | 0.68 (0.61-0.77, p<0.001) |
| Type of surgery | Colon resection | 36 (4.1) | 836 (95.9) | - | - |
|  | Rectum resection | 21 (4.0) | 505 (96.0) | 0.85 (0.76-0.95, p=0.005) | 0.96 (0.83-1.11, p=0.568) |
|  | Stoma formation/closure | 11 (3.0) | 357 (97.0) | 1.00 (0.88-1.13, p=0.993) | 0.93 (0.80-1.09, p=0.395) |
| Operation duration (minutes) | Mean (SD) | 228.6 (94.8) | 193.6 (97.8) | 1.00 (1.00-1.00, p<0.001) | 1.00 (1.00-1.00, p<0.001) |
| Intraoperative anastomosis | No | 28 (5.5) | 481 (94.5) | - | - |
|  | Yes | 40 (3.2) | 1216 (96.8) | 1.26 (1.13-1.40, p<0.001) | 1.16 (1.01-1.34, p=0.036) |
| Intraoperative vascular or organ injury | No | 58 (3.5) | 1622 (96.5) | - | - |
|  | Yes | 10 (11.4) | 78 (88.6) | 0.62 (0.49-0.78, p<0.001) | 0.74 (0.58-0.94, p=0.012) |
| Intraoperative blood transfusion | No | 60 (3.5) | 1649 (96.5) | - | - |
|  | Yes | 8 (13.8) | 50 (86.2) | 0.52 (0.39-0.70, p<0.001) | 0.73 (0.56-0.96, p=0.022) |
| * Includes those who stopped smoking within 6 weeks  Data are percentages unless otherwise stated.  Abbreviations: HR: Hazard Ratio; CI: Confidence Interval; SD: Standard Deviation; BMI: Body Mass Index; ASA: American Society of Anaesthesiologists; IDDM: Insulin-dependent Diabetes Mellitus.  Model metrics: Number in dataframe = 1808, Number in model = 1462, Missing = 346, Number of events = 1413, Concordance = 0.658 (SE = 0.010), R-squared = 0.147, Likelihood ratio test = 232.445 (df = 17, p = 0.000) | | | | | |

Table S5: Cox-proportional hazards regression model after propensity-score matching of factors associated with time to discharge (days)

|  |  | Discharge at 30 days | | Time to discharge (days) | |
| --- | --- | --- | --- | --- | --- |
|  |  | No (n=49) | Yes (n=1414) | Univariable HR (95% CI) | Propensity-score matched HR (95% CI) |
| Intraperitoneal drain insertion | No | 17 (2.5) | 660 (97.5) | - | - |
|  | Yes | 32 (4.1) | 754 (95.9) | 0.71 (0.64-0.79, p<0.001) | 0.58 (0.52-0.66, p<0.001) |
| Age | <70 years | 19 (2.3) | 804 (97.7) | - | - |
|  | ≥70 years | 30 (4.7) | 610 (95.3) | 0.76 (0.69-0.85, p<0.001) | 0.70 (0.61-0.81, p<0.001) |
| Sex | Female | 20 (3.1) | 616 (96.9) | - | - |
|  | Male | 29 (3.5) | 798 (96.5) | 1.01 (0.91-1.12, p=0.854) | 1.15 (1.01-1.31, p=0.035) |
| BMI (kg/m^2^) | Underweight-Normal | 26 (4.5) | 547 (95.5) | - | - |
|  | Overweight | 13 (2.3) | 560 (97.7) | 1.21 (1.07-1.36, p=0.002) | 1.39 (1.21-1.60, p<0.001) |
|  | Obese | 10 (3.2) | 307 (96.8) | 1.05 (0.91-1.20, p=0.536) | 1.09 (0.91-1.30, p=0.350) |
| Smoking status | Never | 27 (3.4) | 769 (96.6) | - | - |
|  | Previous | 6 (1.3) | 440 (98.7) | 1.13 (1.01-1.27, p=0.038) | 1.14 (1.00-1.31, p=0.059) |
|  | Current* | 16 (7.2) | 205 (92.8) | 0.95 (0.81-1.11, p=0.507) | 1.01 (0.85-1.21, p=0.870) |
| ASA score | I-II | 18 (1.8) | 976 (98.2) | - | - |
|  | III-V | 31 (6.6) | 438 (93.4) | 0.72 (0.65-0.81, p<0.001) | 0.75 (0.65-0.86, p<0.001) |
| Diabetes Mellitus | No | 39 (3.2) | 1186 (96.8) | - | - |
|  | non-IDDM | 6 (3.1) | 185 (96.9) | 0.94 (0.81-1.10, p=0.469) | 0.89 (0.74-1.07, p=0.230) |
|  | IDDM | 4 (8.5) | 43 (91.5) | 0.82 (0.61-1.12, p=0.209) | 0.81 (0.58-1.13, p=0.220) |
| Previous abdominal surgery | No | 19 (2.7) | 690 (97.3) | - | - |
|  | Yes | 30 (4.0) | 724 (96.0) | 0.85 (0.77-0.95, p=0.003) | 0.93 (0.81-1.06, p=0.250) |
| Anticoagulation therapy | No | 37 (3.2) | 1115 (96.8) | - | - |
|  | Yes | 12 (3.9) | 299 (96.1) | 0.87 (0.77-0.99, p=0.036) | 0.80 (0.68-0.95, p=0.009) |
| Immunosuppression status | No | 43 (3.4) | 1214 (96.6) | - | - |
|  | Yes | 6 (2.9) | 200 (97.1) | 0.83 (0.72-0.97, p0.018) | 0.99 (0.84-1.17, p=0.910) |
| Underlying pathology | Benign | 19 (4.3) | 419 (95.7) | - | - |
|  | Malignancy | 30 (2.9) | 995 (97.1) | 1.00 (0.89-1.12, p=0.987) | 1.07 (0.92-1.25, p=0.390) |
| Type of surgery | Colon resection | 26 (3.6) | 703 (96.4) | - | - |
|  | Rectum resection | 16 (3.6) | 424 (96.4) | 0.85 (0.75-0.96, p=0.008) | 1.15 (0.99-1.34, p=0.073) |
|  | Stoma formation/closure | 7 (2.4) | 287 (97.6) | 0.99 (0.86-1.13, p=0.868) | 1.06 (0.87-1.30, p=0.560) |
| Operative approach | Minimally invasive | 19 (2.5) | 740 (97.5) | - | - |
|  | Open | 30 (4.3) | 674 (95.7) | 0.66 (0.60-0.73, p<0.001) | 0.60 (0.53-0.69, p<0.001) |
| Perforated bowel | No | 43 (3.0) | 1383 (97.0) | - | - |
|  | Yes | 6 (16.2) | 31 (83.8) | 0.54 (0.38-0.77, p<0.001) | 0.33 (0.22-0.51, p<0.001) |
| Operative contamination | Clean-Contaminated | 41 (3.0) | 1312 (97.0) | - | - |
|  | Contaminated / Dirty | 8 (7.3) | 102 (92.7) | 0.85 (0.69-1.03, p=0.102) | 0.79 (0.62-1.00, p=0.054) |
| Operation duration (minutes) | Mean (SD) | 236.7 (100.9) | 195.6 (96.3) | 1.00 (1.00-1.00, p<0.001) | 1.00 (1.00-1.00, p<0.001) |
| Intraoperative anastomosis | No | 18 (4.6) | 372 (95.4) | - | - |
|  | Yes | 31 (2.9) | 1042 (97.1) | 1.25 (1.11-1.40, p<0.001) | 1.21 (1.05-1.40, p=0.010) |
| Intraoperative vascular or organ injury | No | 40 (2.9) | 1348 (97.1) | - | - |
|  | Yes | 9 (12.0) | 66 (88.0) | 0.59 (0.46-0.75, p<0.001) | 0.71 (0.54-0.93, p=0.014) |
| Intraoperative blood transfusion | No | 42 (3.0) | 1373 (97.0) | - | - |
|  | Yes | 7 (14.6) | 41 (85.4) | 0.52 (0.37-0.71, p<0.001) | 0.48 (0.34-0.68, p<0.001) |
| * Includes those who stopped smoking within 6 weeks  Data are percentages unless otherwise stated.  Abbreviations: HR: Hazard Ratio; CI: Confidence Interval; SD: Standard Deviation; BMI: Body Mass Index; ASA: American Society of Anaesthesiologists; IDDM: Insulin-dependent Diabetes | | | | | |

Table S6: Logistic regression model of factors associated with major postoperative complications (Clavien-Dindo grade III-V)

|  |  | Major postoperative complication (Clavien-Dindo grade III-V) | | | |
| --- | --- | --- | --- | --- | --- |
|  |  | No (n=1648) | Yes (n=127) | Univariable OR (95% CI) | Multilevel OR (95% CI) |
| Intraperitoneal drain insertion | No | 803 (94.5) | 47 (5.5) | - | - |
|  | Prophylactic | 576 (92.2) | 49 (7.8) | 1.45 (0.96-2.20, p=0.077) | 1.16 (0.72-1.87, p=0.548) |
|  | Indication | 269 (89.7) | 31 (10.3) | 1.97 (1.22-3.15, p=0.005) | 1.08 (0.60-1.93, p=0.807) |
| Age (years) | Mean (SD) | 63.8 (14.3) | 67.1 (13.2) | 1.02 (1.00-1.03, p=0.013) | 1.01 (1.00-1.03, p=0.111) |
| Sex | Female | 728 (93.2) | 53 (6.8) | - | - |
|  | Male | 923 (92.6) | 74 (7.4) | 1.10 (0.77-1.59, p=0.605) | - |
| Smoking status | Never | 801 (93.5) | 56 (6.5) | - | - |
|  | Previous | 441 (93.0) | 33 (7.0) | 1.07 (0.68-1.66, p=0.765) | 0.91 (0.56-1.47, p=0.695) |
|  | Current* | 218 (90.8) | 22 (9.2) | 1.44 (0.85-2.38, p=0.163) | 1.57 (0.91-2.71, p=0.108) |
| BMI (kg/m^2^) | Underweight-Normal | 632 (92.7) | 50 (7.3) | - | - |
|  | Overweight | 622 (93.8) | 41 (6.2) | 0.83 (0.54-1.28, p=0.403) | 0.76 (0.47-1.22, p=0.253) |
|  | Obese | 336 (91.1) | 33 (8.9) | 1.24 (0.78-1.96, p=0.356) | 1.01 (0.59-1.71, p=0.974) |
| ASA score | I-II | 1143 (94.5) | 67 (5.5) | - | - |
|  | III-V | 503 (89.3) | 60 (10.7) | 2.03 (1.41-2.93, p<0.001) | 1.52 (0.98-2.36, p=0.059) |
| Previous abdominal surgery | No | 807 (93.1) | 60 (6.9) | - | - |
|  | Yes | 844 (92.6) | 67 (7.4) | 1.07 (0.74-1.54, p=0.722) | - |
| Cardiovascular disease | No | 1387 (93.6) | 95 (6.4) | - | - |
|  | Yes | 264 (89.2) | 32 (10.8) | 1.77 (1.15-2.67, p=0.008) | - |
| Diabetes Mellitus | No | 1402 (93.6) | 96 (6.4) | - | - |
|  | non-IDDM | 201 (90.1) | 22 (9.9) | 1.60 (0.96-2.55, p=0.059) | 1.21 (0.67-2.16, p=0.531) |
|  | IDDM | 42 (82.4) | 9 (17.6) | 3.13 (1.39-6.34, p=0.003) | 2.89 (1.30-6.43, p=0.009) |
| Immunosuppression status | No | 1428 (93.3) | 103 (6.7) | - | - |
|  | Yes | 222 (90.6) | 23 (9.4) | 1.44 (0.88-2.27, p=0.134) | 1.71 (1.01-2.87, p=0.045) |
| Anticoagulation therapy | No | 1296 (93.2) | 94 (6.8) | - | - |
|  | Yes | 355 (91.7) | 32 (8.3) | 1.24 (0.81-1.87, p=0.308) | - |
| Underlying pathology | Benign | 510 (92.7) | 40 (7.3) | - | - |
|  | Malignancy | 1141 (92.9) | 87 (7.1) | 0.97 (0.66-1.45, p=0.887) | - |
| Perforated bowel | No | 1616 (93.0) | 121 (7.0) | - | - |
|  | Yes | 35 (85.4) | 6 (14.6) | 2.29 (0.85-5.17, p=0.067) | 2.52 (0.99-6.45, p=0.054) |
| Operative contamination | Clean-Contaminated | 1535 (92.9) | 117 (7.1) | - | - |
|  | Contaminated / Dirty | 112 (91.8) | 10 (8.2) | 1.17 (0.56-2.19, p=0.645) | - |
| Operative approach | Minimally invasive | 874 (94.7) | 49 (5.3) | - | - |
|  | Open | 775 (90.9) | 78 (9.1) | 1.80 (1.24-2.61, p=0.002) | 1.41 (0.92-2.17, p=0.112) |
| Type of surgery | Colon resection | 812 (92.7) | 64 (7.3) | - | - |
|  | Rectum resection | 486 (92.2) | 41 (7.8) | 1.07 (0.71-1.60, p=0.744) | - |
|  | Stoma formation/closure | 349 (94.1) | 22 (5.9) | 0.80 (0.48-1.30, p=0.382) | - |
| Operation duration (minutes) | Mean (SD) | 192.7 (97.3) | 222.2 (104.2) | 1.00 (1.00-1.00, p=0.001) | 1.00 (1.00-1.00, p=0.011) |
| Intraoperative anastomosis | No | 474 (92.2) | 40 (7.8) | - | - |
|  | Yes | 1173 (93.1) | 87 (6.9) | 0.88 (0.60-1.31, p=0.516) | - |
| Intraoperative vascular or organ injury | No | 1575 (93.3) | 113 (6.7) | - | - |
|  | Yes | 74 (84.1) | 14 (15.9) | 2.64 (1.39-4.68, p=0.002) | 2.21 (1.10-4.42, p=0.025) |
| Intraoperative blood transfusion | No | 1602 (93.3) | 115 (6.7) | - | - |
|  | Yes | 46 (79.3) | 12 (20.7) | 3.63 (1.80-6.84, p<0.001) | 2.43 (1.13-5.19, p=0.023) |
| * Includes those who stopped smoking within 6 weeks  Data are percentages unless otherwise stated.  Abbreviations: OR: Odds Ratio; CI: Confidence Interval; SD: Standard Deviation; BMI: Body Mass Index; ASA: American Society of Anaesthesiologists; IDDM: Insulin-dependent Diabetes Mellitus.  Model metrics: Number in model = 1479, Number of groups = 179, AIC = 745, C-statistic = 0.697 | | | | | |

Table S7: Logistic regression model of factors associated with postoperative intraperitoneal collections

|  |  | Postoperative intraperitoneal collections | | | |
| --- | --- | --- | --- | --- | --- |
|  |  | No (n=1677) | Yes (n=94) | Univariable OR (CI 95%) | Multilevel OR (95% CI) |
| Intraperitoneal drain insertion | No | 819 (96.5) | 30 (3.5) | - | - |
|  | Prophylactic | 583 (93.7) | 39 (6.3) | 1.83 (1.12-2.99, p=0.015) | 1.64 (0.93-2.91, p=0.088) |
|  | Indication | 275 (91.7) | 25 (8.3) | 2.48 (1.42-4.29, p=0.001) | 1.80 (0.88-3.67, p=0.109) |
| Age (years) | Mean (SD) | 64.1 (14.2) | 63.3 (14.7) | 1.00 (0.98-1.01, p=0.617) | 1.00 (0.98-1.01, p=0.668) |
| Sex | Female | 744 (95.5) | 35 (4.5) | - | - |
|  | Male | 935 (94.1) | 59 (5.9) | 1.34 (0.88-2.08, p=0.180) | - |
| Smoking status | Never | 809 (94.7) | 45 (5.3) | - | - |
|  | Previous | 445 (93.9) | 29 (6.1) | 1.17 (0.72-1.88, p=0.519) | 1.05 (0.62-1.76, p=0.862) |
|  | Current* | 230 (96.2) | 9 (3.8) | 0.70 (0.32-1.39, p=0.345) | 0.62 (0.29-1.34, p=0.227) |
| BMI (kg/m^2^) | Underweight-Normal | 636 (93.4) | 45 (6.6) | - | - |
|  | Overweight | 631 (95.6) | 29 (4.4) | 0.65 (0.40-1.04, p=0.078) | 0.60 (0.35-1.02, p=0.061) |
|  | Obese | 351 (95.4) | 17 (4.6) | 0.68 (0.38-1.19, p=0.195) | 0.52 (0.27-1.00, p=0.052) |
| ASA score | I-II | 1149 (95.2) | 58 (4.8) | - | - |
|  | III-V | 525 (93.6) | 36 (6.4) | 1.36 (0.88-2.07, p=0.161) | 1.78 (1.06-2.97, p=0.029) |
| Previous abdominal surgery | No | 816 (94.6) | 47 (5.4) | - | - |
|  | Yes | 863 (94.8) | 47 (5.2) | 0.95 (0.62-1.43, p=0.792) | - |
| Cardiovascular disease | No | 1401 (94.8) | 77 (5.2) | - | - |
|  | Yes | 278 (94.2) | 17 (5.8) | 1.11 (0.63-1.86, p=0.699) | - |
| Diabetes Mellitus | No | 1417 (94.8) | 77 (5.2) | - | - |
|  | non-IDDM | 211 (95.0) | 11 (5.0) | 0.96 (0.48-1.76, p=0.900) | - |
|  | IDDM | 45 (90.0) | 5 (10.0) | 2.04 (0.69-4.85, p=0.141) | - |
| Immunosuppression status | No | 1447 (94.8) | 80 (5.2) | - | - |
|  | Yes | 230 (94.3) | 14 (5.7) | 1.10 (0.59-1.91, p=0.747) | 0.86 (0.44-1.69, p=0.670) |
| Anticoagulation therapy | No | 1311 (94.7) | 74 (5.3) | - | - |
|  | Yes | 367 (94.8) | 20 (5.2) | 0.97 (0.57-1.57, p=0.892) | - |
| Underlying pathology | Benign | 515 (93.6) | 35 (6.4) | - | - |
|  | Malignancy | 1164 (95.2) | 59 (4.8) | 0.75 (0.49-1.16, p=0.182) | 0.46 (0.26-0.80, p=0.006) |
| Perforated bowel | No | 1643 (94.9) | 89 (5.1) | - | - |
|  | Yes | 36 (87.8) | 5 (12.2) | 2.56 (0.86-6.14, p=0.054) | - |
| Operative contamination | Clean-Contaminated | 1564 (94.9) | 84 (5.1) | - | - |
|  | Contaminated / Dirty | 114 (93.4) | 8 (6.6) | 1.31 (0.57-2.61, p=0.484) | 0.80 (0.32-1.97, p=0.623) |
| Operative approach | Minimally invasive | 873 (94.9) | 47 (5.1) | - | - |
|  | Open | 806 (94.6) | 46 (5.4) | 1.06 (0.70-1.61, p=0.784) | - |
| Type of surgery | Colon resection | 829 (94.7) | 46 (5.3) | - | - |
|  | Rectum resection | 486 (92.6) | 39 (7.4) | 1.45 (0.93-2.25, p=0.101) | 1.32 (0.77-2.27, p=0.308) |
|  | Stoma formation/closure | 361 (97.6) | 9 (2.4) | 0.45 (0.20-0.88, p=0.031) | 0.26 (0.10-0.70, p=0.007) |
| Operation duration (minutes) | Mean (SD) | 193.1 (97.0) | 222.3 (111.7) | 1.00 (1.00-1.00, p=0.006) | 1.00 (1.00-1.00, p=0.278) |
| Intraoperative anastomosis | No | 486 (94.7) | 27 (5.3) | - | - |
|  | Yes | 1190 (94.7) | 67 (5.3) | 1.01 (0.65-1.63, p=0.955) | 1.29 (0.73-2.26, p=0.383) |
| Intraoperative vascular or organ injury | No | 1602 (95.1) | 82 (4.9) | - | - |
|  | Yes | 76 (86.4) | 12 (13.6) | 3.08 (1.54-5.70, p=0.001) | 2.36 (1.09-5.13, p=0.029) |
| Intraoperative blood transfusion | No | 1626 (94.9) | 87 (5.1) | - | - |
|  | Yes | 51 (87.9) | 7 (12.1) | 2.57 (1.04-5.47, p=0.024) | - |
| * Includes those who stopped smoking within 6 weeks  Data are percentages unless otherwise stated.  Abbreviations: OR: Odds Ratio; CI: Confidence Interval; SD: Standard Deviation; BMI: Body Mass Index; ASA: American Society of Anaesthesiologists; IDDM: Insulin-dependent Diabetes Mellitus.  Model metrics: Number in model = 1475, Number of groups = 179, AIC = 607.1, C-statistic = 0.719 | | | | | |

Table S8: Logistic regression model of factors associated with surgical site infections

|  |  | Surgical site infection | | | |
| --- | --- | --- | --- | --- | --- |
|  |  | No (n=1635) | Yes (n=143) | Univariable OR (95% CI) | Multilevel OR (95% CI) |
| Intraperitoneal drain insertion | No | 798 (93.6) | 55 (6.4) | - | - |
|  | Prophylactic | 566 (90.4) | 60 (9.6) | 1.54 (1.05-2.26, p=0.027) | 1.28 (0.82-1.99, p=0.270) |
|  | Indication | 271 (90.6) | 28 (9.4) | 1.50 (0.92-2.39, p=0.095) | 1.17 (0.67-2.05, p=0.581) |
| Age (years) | Mean (SD) | 64.1 (14.4) | 63.8 (12.2) | 1.00 (0.99-1.01, p=0.804) | 1.01 (0.99-1.02, p=0.316) |
| Sex | Female | 725 (92.5) | 59 (7.5) | - | - |
|  | Male | 912 (91.6) | 84 (8.4) | 1.13 (0.80-1.61, p=0.484) | - |
| Smoking status | Never | 784 (91.5) | 73 (8.5) | - | - |
|  | Previous | 437 (91.8) | 39 (8.2) | 0.96 (0.63-1.43, p=0.838) | 0.83 (0.54-1.29, p=0.410) |
|  | Current* | 217 (90.8) | 22 (9.2) | 1.09 (0.65-1.77, p=0.739) | 1.06 (0.61-1.81, p=0.845) |
| BMI (kg/m^2^) | Underweight-Normal | 639 (93.6) | 44 (6.4) | - | - |
|  | Overweight | 607 (92.0) | 53 (8.0) | 1.27 (0.84-1.93, p=0.262) | 1.24 (0.79-1.94, p=0.345) |
|  | Obese | 328 (88.4) | 43 (11.6) | 1.90 (1.22-2.96, p=0.004) | 1.69 (1.03-2.77, p=0.037) |
| ASA score | I-II | 1120 (92.5) | 91 (7.5) | - | - |
|  | III-V | 512 (90.8) | 52 (9.2) | 1.25 (0.87-1.78, p=0.220) | 1.11 (0.73-1.69, p=0.634) |
| Previous abdominal surgery | No | 801 (92.4) | 66 (7.6) | - | - |
|  | Yes | 835 (91.6) | 77 (8.4) | 1.12 (0.79-1.58, p=0.520) | - |
| Cardiovascular disease | No | 1368 (92.1) | 117 (7.9) | - | - |
|  | Yes | 269 (91.2) | 26 (8.8) | 1.13 (0.71-1.74, p=0.590) | - |
| Diabetes Mellitus | No | 1382 (92.2) | 117 (7.8) | - | - |
|  | non-IDDM | 205 (91.9) | 18 (8.1) | 1.04 (0.60-1.70, p=0.890) | 0.84 (0.46-1.53, p=0.569) |
|  | IDDM | 44 (86.3) | 7 (13.7) | 1.88 (0.76-4.01, p=0.131) | 1.81 (0.76-4.31, p=0.181) |
| Immunosuppression status | No | 1420 (92.6) | 113 (7.4) | - | - |
|  | Yes | 216 (88.2) | 29 (11.8) | 1.69 (1.08-2.57, p=0.018) | 1.43 (0.87-2.36, p=0.157) |
| Anticoagulation therapy | No | 1283 (92.2) | 108 (7.8) | - | - |
|  | Yes | 353 (91.0) | 35 (9.0) | 1.18 (0.78-1.74, p=0.421) | - |
| Underlying pathology | Benign | 498 (90.4) | 53 (9.6) | - | - |
|  | Malignancy | 1138 (92.7) | 90 (7.3) | 0.74 (0.52-1.07, p=0.101) | 0.64 (0.42-0.99, p=0.046) |
| Perforated bowel | No | 1605 (92.3) | 134 (7.7) | - | - |
|  | Yes | 32 (78.0) | 9 (22.0) | 3.37 (1.49-6.92, p=0.002) | 2.56 (1.09-6.01, p=0.031) |
| Operative contamination | Clean-Contaminated | 1525 (92.1) | 130 (7.9) | - | - |
|  | Contaminated / Dirty | 109 (89.3) | 13 (10.7) | 1.40 (0.73-2.47, p=0.275) | - |
| Operative approach | Minimally invasive | 872 (94.1) | 55 (5.9) | - | - |
|  | Open | 764 (89.7) | 88 (10.3) | 1.83 (1.29-2.61, p=0.001) | 1.87 (1.26-2.78, p=0.002) |
| Type of surgery | Colon resection | 812 (92.5) | 66 (7.5) | - | - |
|  | Rectum resection | 476 (90.3) | 51 (9.7) | 1.32 (0.90-1.93, p=0.157) | - |
|  | Stoma formation/closure | 346 (93.0) | 26 (7.0) | 0.92 (0.57-1.46, p=0.744) | - |
| Operation duration (minutes) | Mean (SD) | 192.5 (97.0) | 220.4 (105.2) | 1.00 (1.00-1.00, p=0.001) | 1.00 (1.00-1.00, p=0.004) |
| Intraoperative anastomosis | No | 464 (90.6) | 48 (9.4) | - | - |
|  | Yes | 1169 (92.5) | 95 (7.5) | 0.79 (0.55-1.14, p=0.193) | - |
| Intraoperative vascular or organ injury | No | 1562 (92.4) | 129 (7.6) | - | - |
|  | Yes | 74 (84.1) | 14 (15.9) | 2.29 (1.21-4.05, p=0.007) | - |
| Intraoperative blood transfusion | No | 1587 (92.3) | 133 (7.7) | - | - |
|  | Yes | 48 (82.8) | 10 (17.2) | 2.49 (1.16-4.83, p=0.011) | - |
| * Includes those who stopped smoking within 6 weeks  Data are percentages unless otherwise stated.  Abbreviations: OR: Odds Ratio; CI: Confidence Interval; SD: Standard Deviation; BMI: Body Mass Index; ASA: American Society of Anaesthesiologists; IDDM: Insulin-dependent Diabetes Mellitus.  Model metrics: Number in model = 1482, Number of groups = 180, AIC = 856.3, C-statistic = 0.725 | | | | | |

Table S9: Cox-proportional hazards regression model of factors associated with time to postoperative intraperitoneal collection diagnosis (days)

|  |  | Collection diagnosis at 30 days | | Time to postoperative intraperitoneal collection diagnosis (days) | |
| --- | --- | --- | --- | --- | --- |
|  |  | No (n=1677) | Yes (n=94) | Univariable HR (95% CI) | Multilevel HR (95% CI) |
| Intraperitoneal drain insertion | No | 819 (96.5) | 30 (3.5) | - | - |
|  | Prophylactic | 583 (93.7) | 39 (6.3) | 0.61 (0.37-1.00, p=0.049) | 0.87 (0.52-1.47, p=0.606) |
|  | Indication | 275 (91.7) | 25 (8.3) | 0.95 (0.55-1.63, p=0.851) | 1.03 (0.59-1.81, p=0.924) |
| Age (years) | Mean (SD) | 64.1 (14.2) | 63.3 (14.7) | 1.00 (0.99-1.02, p=0.552) | 1.01 (0.98-1.04, p=0.422) |
| Sex | Female | 744 (95.5) | 35 (4.5) | - | - |
|  | Male | 935 (94.1) | 59 (5.9) | 0.93 (0.61-1.42, p=0.730) | - |
| Smoking status | Never | 809 (94.7) | 45 (5.3) | - | - |
|  | Previous | 445 (93.9) | 29 (6.1) | 0.93 (0.58-1.52, p=0.786) | 0.99 (0.59-1.65, p=0.956) |
|  | Current* | 230 (96.2) | 9 (3.8) | 0.94 (0.46-1.93, p=0.871) | 0.69 (0.34-1.42, p=0.317) |
| BMI (kg/m^2^) | Underweight-Normal | 636 (93.4) | 45 (6.6) | - | - |
|  | Overweight | 631 (95.6) | 29 (4.4) | 0.65 (0.40-1.06, p=0.083) | 0.92 (0.53-1.61, p=0.780) |
|  | Obese | 351 (95.4) | 17 (4.6) | 0.84 (0.48-1.46, p=0.531) | 0.89 (0.39-2.02, p=0.782) |
| ASA score | I-II | 1149 (95.2) | 58 (4.8) | - | - |
|  | III-V | 525 (93.6) | 36 (6.4) | 0.88 (0.58-1.34, p=0.549) | 0.44 (0.25-0.77, p=0.004) |
| Previous abdominal surgery | No | 816 (94.6) | 47 (5.4) | - | - |
|  | Yes | 863 (94.8) | 47 (5.2) | 0.81 (0.53-1.22, p=0.305) | - |
| Cardiovascular disease | No | 1401 (94.8) | 77 (5.2) | - | - |
|  | Yes | 278 (94.2) | 17 (5.8) | 0.93 (0.55-1.58, p=0.789) | - |
| Diabetes Mellitus | No | 1417 (94.8) | 77 (5.2) | - | - |
|  | non-IDDM | 211 (95.0) | 11 (5.0) | 1.12 (0.59-2.13, p=0.719) | 0.88 (0.37-2.10, p=0.781) |
|  | IDDM | 45 (90.0) | 5 (10.0) | 1.76 (0.70-4.40, p=0.229) | 3.10 (1.32-7.26, p=0.009) |
| Immunosuppression status | No | 1447 (94.8) | 80 (5.2) | - | - |
|  | Yes | 230 (94.3) | 14 (5.7) | 1.31 (0.72-2.36, p=0.375) | 2.82 (1.46-5.44, p=0.002) |
| Anticoagulation therapy | No | 1311 (94.7) | 74 (5.3) | - | - |
|  | Yes | 367 (94.8) | 20 (5.2) | 0.73 (0.44-1.23, p=0.242) | - |
| Underlying pathology | Benign | 515 (93.6) | 35 (6.4) | - | - |
|  | Malignancy | 1164 (95.2) | 59 (4.8) | 1.56 (1.01-2.41, p=0.047) | 1.21 (0.57-2.59, p=0.616) |
| Perforated bowel | No | 1643 (94.9) | 89 (5.1) | - | - |
|  | Yes | 36 (87.8) | 5 (12.2) | 0.98 (0.40-2.42, p=0.963) | - |
| Operative contamination | Clean-Contaminated | 1564 (94.9) | 84 (5.1) | - | - |
|  | Contaminated / Dirty | 114 (93.4) | 8 (6.6) | 1.79 (0.86-3.71, p=0.119) | 2.71 (0.98-7.50, p=0.056) |
| Operative approach | Minimally invasive | 873 (94.9) | 47 (5.1) | - | - |
|  | Open | 806 (94.6) | 46 (5.4) | 0.88 (0.58-1.33, p=0.541) | - |
| Type of surgery | Colon resection | 829 (94.7) | 46 (5.3) | - | - |
|  | Rectum resection | 486 (92.6) | 39 (7.4) | 0.95 (0.61-1.46, p=0.804) | 0.84 (0.54-1.33, p=0.464) |
|  | Stoma formation/closure | 361 (97.6) | 9 (2.4) | 0.73 (0.34-1.56, p=0.420) | 0.29 (0.06-1.46, p=0.134) |
| Operation duration (minutes) | Mean (SD) | 193.1 (97.0) | 222.3 (111.7) | 1.00 (1.00-1.00, p=0.378) | 1.00 (1.00-1.00, p=0.246) |
| Intraoperative anastomosis | No | 486 (94.7) | 27 (5.3) | - | - |
|  | Yes | 1190 (94.7) | 67 (5.3) | 1.04 (0.65-1.65, p=0.883) | 1.16 (0.72-1.87, p=0.549) |
| Intraoperative vascular or organ injury | No | 1602 (95.1) | 82 (4.9) | - | - |
|  | Yes | 76 (86.4) | 12 (13.6) | 1.39 (0.74-2.62, p=0.310) | 2.98 (1.21-7.32, p=0.017) |
| Intraoperative blood transfusion | No | 1626 (94.9) | 87 (5.1) | - | - |
|  | Yes | 51 (87.9) | 7 (12.1) | 0.74 (0.34-1.62, p=0.454) | - |
| * Includes those who stopped smoking within 6 weeks  Data are percentages unless otherwise stated.  Abbreviations: HR: Hazard Ratio; CI: Confidence Interval; SD: Standard Deviation; BMI: Body Mass Index; ASA: American Society of Anaesthesiologists; IDDM: Insulin-dependent Diabetes Mellitus.  Model metrics: Number in dataframe = 1808, Number in model = 79, Missing = 1729, Number of events = 79, Concordance = 0.674 (SE = 0.044), R-squared = 0.257, Likelihood ratio test = 23.510 (df = 18, p = 0.172) | | | | | |

Table S10: Logistic regression model after propensity-score matching of factors associated with surgical site infections

|  |  | Surgical site infection | | | |
| --- | --- | --- | --- | --- | --- |
|  |  | No (n=1347) | Yes (n=127) | Univariable OR (95% CI) | Propensity-score matched OR (95% CI) |
| Intraperitoneal drain insertion | No | 634 (93.2) | 46 (6.8) | - | - |
|  | Yes | 713 (89.8) | 81 (10.2) | 1.57 (1.08-2.30, p=0.020) | 2.47 (1.50-4.05, p<0.001) |
| Age | <70 years | 746 (89.9) | 84 (10.1) | - | - |
|  | ≥70 years | 601 (93.3) | 43 (6.7) | 0.64 (0.43-0.93, p=0.020) | 0.77 (0.46-1.28, p=0.312) |
| Sex | Female | 589 (92.0) | 51 (8.0) | - | - |
|  | Male | 758 (90.9) | 76 (9.1) | 1.16 (0.80-1.69, p=0.438) | 1.22 (0.77-1.95, p=0.401) |
| BMI (kg/m^2^) | Underweight-Normal | 539 (92.9) | 41 (7.1) | - | - |
|  | Overweight | 526 (91.5) | 49 (8.5) | 1.22 (0.80-1.89, p=0.358) | 1.02 (0.61-1.71, p=0.937) |
|  | Obese | 282 (88.4) | 37 (11.6) | 1.72 (1.08-2.75, p=0.022) | 1.38 (0.76-2.52, p=0.293) |
| Smoking status | Never | 731 (91.3) | 70 (8.7) | - | - |
|  | Previous | 412 (92.0) | 36 (8.0) | 0.91 (0.59-1.38, p=0.669) | 0.60 (0.36-1.02, p=0.061) |
|  | Current* | 204 (90.7) | 21 (9.3) | 1.07 (0.63-1.76, p=0.782) | 0.57 (0.29-1.10, p=0.093) |
| ASA score | I-II | 922 (91.9) | 81 (8.1) | - | - |
|  | III-V | 425 (90.2) | 46 (9.8) | 1.23 (0.84-1.79, p=0.281) | 1.31 (0.79-2.18, p=0.297) |
| Diabetes Mellitus | No | 1129 (91.5) | 105 (8.5) | - | - |
|  | non-IDDM | 178 (92.2) | 15 (7.8) | 0.91 (0.50-1.55, p=0.732) | 0.54 (0.24-1.20, p=0.132) |
|  | IDDM | 40 (85.1) | 7 (14.9) | 1.88 (0.76-4.06, p=0.134) | 1.86 (0.70-4.94, p=0.212) |
| Previous abdominal surgery | No | 657 (91.9) | 58 (8.1) | - | - |
|  | Yes | 690 (90.9) | 69 (9.1) | 1.13 (0.79-1.64, p=0.503) | 0.96 (0.59-1.57, p=0.874) |
| Anticoagulation therapy | No | 1065 (91.7) | 96 (8.3) | - | - |
|  | Yes | 282 (90.1) | 31 (9.9) | 1.22 (0.79-1.85, p=0.361) | 1.53 (0.85-2.76, p=0.156) |
| Immunosuppression status | No | 1164 (91.9) | 103 (8.1) | - | - |
|  | Yes | 183 (88.4) | 24 (11.6) | 1.48 (0.91-2.34, p=0.101) | 1.40 (0.79-2.49, p=0.250) |
| Underlying pathology | Benign | 395 (89.2) | 48 (10.8) | - | - |
|  | Malignancy | 952 (92.3) | 79 (7.7) | 0.68 (0.47-1.00, p=0.048) | 0.55 (0.33-0.93, p=0.025) |
| Type of surgery | Colon resection | 677 (92.1) | 58 (7.9) | - | - |
|  | Rectum resection | 396 (89.8) | 45 (10.2) | 1.33 (0.88-1.99, p=0.175) | 0.77 (0.45-1.32, p=0.335) |
|  | Stoma formation/closure | 274 (91.9) | 24 (8.1) | 1.02 (0.61-1.66, p=0.930) | 0.77 (0.37-1.62, p=0.495) |
| Operative approach | Minimally invasive | 716 (93.8) | 47 (6.2) | - | - |
|  | Open | 631 (88.7) | 80 (11.3) | 1.93 (1.33-2.83, p=0.001) | 1.97 (1.18-3.28, p=0.010) |
| Perforated bowel | No | 1317 (91.7) | 119 (8.3) | - | - |
|  | Yes | 30 (78.9) | 8 (21.1) | 2.95 (1.24-6.29, p=0.008) | 2.94 (1.11-7.75, p=0.030) |
| Operative contamination | Clean-Contaminated | 1247 (91.6) | 115 (8.4) | - | - |
|  | Contaminated / Dirty | 100 (89.3) | 12 (10.7) | 1.30 (0.66-2.35, p=0.412) | 0.70 (0.32-1.54, p=0.380) |
| Operation duration (minutes) | Mean (SD) | 194.3 (95.7) | 223.0 (104.7) | 1.00 (1.00-1.00, p=0.002) | 1.00 (1.00-1.01, p=0.004) |
| Intraoperative anastomosis | No | 352 (89.3) | 42 (10.7) | - | - |
|  | Yes | 995 (92.1) | 85 (7.9) | 0.72 (0.49-1.06, p=0.092) | 0.71 (0.43-1.16, p=0.172) |
| Intraoperative vascular or organ injury | No | 1285 (91.9) | 114 (8.1) | - | - |
|  | Yes | 62 (82.7) | 13 (17.3) | 2.36 (1.21-4.30, p=0.007) | 1.35 (0.61-2.97, p=0.461) |
| Intraoperative blood transfusion | No | 1309 (91.8) | 117 (8.2) | - | - |
|  | Yes | 38 (79.2) | 10 (20.8) | 2.94 (1.36-5.84, p=0.003) | 2.90 (1.21-6.95, p=0.017) |
| * Includes those who stopped smoking within 6 weeks  Data are percentages unless otherwise stated.  Abbreviations: OR: Odds Ratio; CI: Confidence Interval; SD: Standard Deviation; BMI: Body Mass Index; ASA: American Society of Anaesthesiologists; IDDM: Insulin-dependent Diabetes Mellitus.  Model metrics: Number in model = 1474, Number of groups = 433, AIC = 725.6, C-statistic = 0.767 | | | | | |

Table S11: Logistic regression model after propensity-score matching of factors associated with major postoperative complications (Clavien-Dindo grade III-V)

|  |  | Major postoperative complication (Calvien-Dindo grade III-V) | | | |
| --- | --- | --- | --- | --- | --- |
|  |  | No (n=1366) | Yes (n=107) | Univariable OR (95% CI) | Propensity-score matched OR (95% CI) |
| Intraperitoneal drain insertion | No | 642 (94.4) | 38 (5.6) | - | - |
|  | Yes | 724 (91.3) | 69 (8.7) | 1.61 (1.07-2.45, p=0.023) | 1.09 (0.68-1.75, p=0.709) |
| Age | <70 years | 775 (93.6) | 53 (6.4) | - | - |
|  | ≥70 years | 591 (91.6) | 54 (8.4) | 1.34 (0.90-1.98, p=0.149) | 1.62 (0.93-2.79, p=0.086) |
| Sex | Female | 592 (92.6) | 47 (7.4) | - | - |
|  | Male | 774 (92.8) | 60 (7.2) | 0.98 (0.66-1.46, p=0.906) | 1.00 (0.60-1.67, p=0.998) |
| BMI (kg/m^2^) | Underweight-Normal | 534 (92.2) | 45 (7.8) | - | - |
|  | Overweight | 542 (94.1) | 34 (5.9) | 0.74 (0.47-1.18, p=0.210) | 0.72 (0.41-1.27, p=0.259) |
|  | Obese | 290 (91.2) | 28 (8.8) | 1.15 (0.69-1.86, p=0.589) | 1.00 (0.52-1.93, p=0.989) |
| Smoking status | Never | 747 (93.3) | 54 (6.7) | - | - |
|  | Previous | 416 (93.1) | 31 (6.9) | 1.03 (0.65-1.62, p=0.896) | 1.13 (0.65-1.97, p=0.662) |
|  | Current* | 203 (90.2) | 22 (9.8) | 1.50 (0.88-2.49, p=0.127) | 1.54 (0.79-3.00, p=0.205) |
| ASA score | I-II | 945 (94.3) | 57 (5.7) | - | - |
|  | III-V | 421 (89.4) | 50 (10.6) | 1.97 (1.32-2.93, p=0.001) | 1.72 (1.01-2.92, p=0.047) |
| Diabetes Mellitus | No | 1152 (93.4) | 81 (6.6) | - | - |
|  | non-IDDM | 176 (91.2) | 17 (8.8) | 1.37 (0.77-2.32, p=0.255) | 1.41 (0.72-2.78, p=0.321) |
|  | IDDM | 38 (80.9) | 9 (19.1) | 3.37 (1.48-6.92, p=0.002) | 3.88 (1.47-10.20, p=0.006) |
| Previous abdominal surgery | No | 663 (92.6) | 53 (7.4) | - | - |
|  | Yes | 703 (92.9) | 54 (7.1) | 0.96 (0.65-1.43, p=0.843) | 0.70 (0.41-1.17, p=0.174) |
| Anticoagulation therapy | No | 1081 (93.1) | 80 (6.9) | - | - |
|  | Yes | 285 (91.3) | 27 (8.7) | 1.28 (0.80-1.99, p=0.288) | 1.25 (0.67-2.36, p=0.485) |
| Immunosuppression status | No | 1182 (93.3) | 85 (6.7) | - | - |
|  | Yes | 184 (89.3) | 22 (10.7) | 1.66 (0.99-2.68, p=0.044) | 1.81 (0.96-3.42, p=0.067) |
| Underlying pathology | Benign | 411 (93.0) | 31 (7.0) | - | - |
|  | Malignancy | 955 (92.6) | 76 (7.4) | 1.06 (0.69-1.65, p=0.808) | 0.72 (0.39-1.35, p=0.305) |
| Type of surgery | Colon resection | 682 (92.8) | 53 (7.2) | - | - |
|  | Rectum resection | 404 (91.6) | 37 (8.4) | 1.18 (0.76-1.82, p=0.462) | 0.74 (0.40-1.37, p=0.337) |
|  | Stoma formation/closure | 280 (94.3) | 17 (5.7) | 0.78 (0.43-1.34, p=0.391) | 0.75 (0.31-1.83, 0.529) |
| Operative approach | Minimally invasive | 718 (94.2) | 44 (5.8) | - | - |
|  | Open | 648 (91.1) | 63 (8.9) | 1.59 (1.07-2.38, p=0.024) | 2.07 (1.17-3.68, p=0.013) |
| Perforated bowel | No | 1334 (93.0) | 101 (7.0) | - | - |
|  | Yes | 32 (84.2) | 6 (15.8) | 2.48 (0.92-5.66, p=0.047) | 5.09 (1.81-14.34, p=0.002) |
| Operative contamination | Clean-Contaminated | 1262 (92.7) | 99 (7.3) | - | - |
|  | Contaminated / Dirty | 104 (92.9) | 8 (7.1) | 0.98 (0.43-1.95, p=0.959) | 1.57 (0.72-3.43, p=0.255) |
| Operation duration (minutes) | Mean (SD) | 194.3 (95.6) | 228.0 (107.1) | 1.00 (1.00-1.01, p=0.001) | 1.00 (1.00-1.01, p=0.001) |
| Intraoperative anastomosis | No | 362 (91.9) | 32 (8.1) | - | - |
|  | Yes | 1004 (93.0) | 75 (7.0) | 0.85 (0.55-1.32, p=0.444) | 0.89 (0.52-1.53, p=0.681) |
| Intraoperative vascular or organ injury | No | 1305 (93.3) | 93 (6.7) | - | - |
|  | Yes | 61 (81.3) | 14 (18.7) | 3.22 (1.68-5.82, p<0.001) | 3.44 (1.59-7.45, p=0.002) |
| Intraoperative blood transfusion | No | 1330 (93.3) | 95 (6.7) | - | - |
|  | Yes | 36 (75.0) | 12 (25.0) | 4.67 (2.26-9.02, p<0.001) | 3.66 (1.43-9.37, p=0.007) |
| * Includes those who stopped smoking within 6 weeks  Data are percentages unless otherwise stated.  Abbreviations: OR: Odds Ratio; CI: Confidence Interval; SD: Standard Deviation; BMI: Body Mass Index; ASA: American Society of Anaesthesiologists; IDDM: Insulin-dependent Diabetes Mellitus.  Model metrics: Number in model = 1473, Number of groups = 433, AIC = 731.5, C-statistic = 0.855 | | | | | |

Table S12: Logistic regression model after propensity-score matching of factors associated with postoperative intraperitoneal collections

|  |  | Postoperative intraperitoneal collections | | | |
| --- | --- | --- | --- | --- | --- |
|  |  | No (n=1391) | Yes (n=79) | Univariable OR (95% CI) | Propensity-score matched OR (95% CI) |
| Intraperitoneal drain insertion | No | 656 (96.6) | 23 (3.4) | - | - |
|  | Yes | 735 (92.9) | 56 (7.1) | 2.17 (1.34-3.64, p=0.002) | 1.33 (0.79-2.23, p=0.287) |
| Age | <70 years | 787 (95.2) | 40 (4.8) | - | - |
|  | ≥70 years | 604 (93.9) | 39 (6.1) | 1.27 (0.81-2.00, p=0.301) | 1.45 (0.81-2.60, p=0.213) |
| Sex | Female | 608 (95.4) | 29 (4.6) | - | - |
|  | Male | 783 (94.0) | 50 (6.0) | 1.34 (0.84-2.16, p=0.223) | 1.54 (0.87-2.73, p=0.139) |
| BMI (kg/m^2^) | Underweight-Normal | 539 (93.1) | 40 (6.9) | - | - |
|  | Overweight | 549 (95.6) | 25 (4.4) | 0.61 (0.36-1.02, p=0.062) | 0.67 (0.36-1.23, p=0.193) |
|  | Obese | 303 (95.6) | 14 (4.4) | 0.62 (0.32-1.14, p=0.137) | 0.83 (0.41-1.67, p=0.592) |
| Smoking status | Never | 754 (94.5) | 44 (5.5) | - | - |
|  | Previous | 421 (94.2) | 26 (5.8) | 1.06 (0.63-1.73, p=0.821) | 1.12 (0.61-2.04, p=0.713) |
|  | Current* | 216 (96.0) | 9 (4.0) | 0.71 (0.32-1.42, p=0.368) | 1.09 (0.52-2.29, p=0.826) |
| ASA score | I-II | 955 (95.4) | 46 (4.6) | - | - |
|  | III-V | 436 (93.0) | 33 (7.0) | 1.57 (0.98-2.48, p=0.055) | 2.03 (1.14-3.61, p=0.016) |
| Diabetes Mellitus | No | 1167 (94.8) | 64 (5.2) | - | - |
|  | non-IDDM | 183 (94.8) | 10 (5.2) | 1.00 (0.47-1.89, p=0.992) | 1.07 (0.50-2.27, p=0.861) |
|  | IDDM | 41 (89.1) | 5 (10.9) | 2.22 (0.75-5.34, p=0.103) | 2.40 (0.81-7.07, p=0.113) |
| Previous abdominal surgery | No | 671 (94.0) | 43 (6.0) | - | - |
|  | Yes | 720 (95.2) | 36 (4.8) | 0.78 (0.49-1.23, p=0.285) | 0.66 (0.37-1.15, p=0.143) |
| Anticoagulation therapy | No | 1096 (94.6) | 62 (5.4) | - | - |
|  | Yes | 295 (94.6) | 17 (5.4) | 1.02 (0.57-1.73, p=0.948) | 0.97 (0.48-1.96, p=0.924) |
| Immunosuppression status | No | 1197 (94.7) | 67 (5.3) | - | - |
|  | Yes | 194 (94.2) | 12 (5.8) | 1.11 (0.56-2.01, p=0.757) | 0.81 (0.39-1.70, p=0.580) |
| Underlying pathology | Benign | 413 (93.4) | 29 (6.6) | - | - |
|  | Malignancy | 978 (95.1) | 50 (4.9) | 0.73 (0.46-1.18, p=0.187) | 0.30 (0.16-0.58, p<0.001) |
| Type of surgery | Colon resection | 693 (94.5) | 40 (5.5) | - | - |
|  | Rectum resection | 407 (92.3) | 34 (7.7) | 1.45 (0.90-2.32, p=0.126) | 1.16 (0.61-2.21, p=0.641) |
|  | Stoma formation/closure | 291 (98.3) | 5 (1.7) | 0.30 (0.10-0.69, p=0.011) | 0.24 (0.07-0.83, p=0.024) |
| Operative approach | Minimally invasive | 719 (94.7) | 40 (5.3) | - | - |
|  | Open | 672 (94.5) | 39 (5.5) | 1.04 (0.66-1.64, p=0.855) | 1.55 (0.85-2.81, p=0150) |
| Perforated bowel | No | 1358 (94.8) | 74 (5.2) | - | - |
|  | Yes | 33 (86.8) | 5 (13.2) | 2.78 (0.93-6.74, p=0.039) | 3.57 (1.17-10.87, p=0.025) |
| Operative contamination | Clean-Contaminated | 1285 (94.6) | 73 (5.4) | - | - |
|  | Contaminated / Dirty | 106 (94.6) | 6 (5.4) | 1.00 (0.38-2.17, p=0.993) | 2.06 (0.94-4.53, p=0.072) |
| Operation duration (minutes) | Mean (SD) | 195.2 (96.0) | 223.7 (107.0) | 1.00 (1.00-1.00, p=0.011) | 1.00 (1.00-1.01, p=0.061) |
| Intraoperative anastomosis | No | 373 (94.7) | 21 (5.3) | - | - |
|  | Yes | 1018 (94.6) | 58 (5.4) | 1.01 (0.62-1.73, p=0.964) | 1.15 (0.63-2.08, p=0.656) |
| Intraoperative vascular or organ injury | No | 1326 (95.1) | 69 (4.9) | - | - |
|  | Yes | 65 (86.7) | 10 (13.3) | 2.96 (1.38-5.77, p=0.003) | 4.26 (1.92-9.45, p<0.001) |
| Intraoperative blood transfusion | No | 1349 (94.9) | 73 (5.1) | - | - |
|  | Yes | 42 (87.5) | 6 (12.5) | 2.64 (0.98-5.97, p=0.032) | 1.64 (0.57-4.72, p=0.361) |
| * Includes those who stopped smoking within 6 weeks  Data are percentages unless otherwise stated.  Abbreviations: OR: Odds Ratio; CI: Confidence Interval; SD: Standard Deviation; BMI: Body Mass Index; ASA: American Society of Anaesthesiologists; IDDM: Insulin-dependent Diabetes Mellitus.  Model metrics: Number in model = 1470, Number of groups = 433, AIC = 623.5, C-statistic = 0.849 | | | | | |

Table S13: Cox-proportional hazards regression model after propensity-score matching of factors associated with time to postoperative intraperitoneal collection diagnosis (days)

|  |  | Collection diagnosis at 30 days | | Time to postoperative intraperitoneal collection diagnosis (days) | |
| --- | --- | --- | --- | --- | --- |
|  |  | No (n=1392) | Yes (n=78) | Univariable HR (95% CI) | Propensity-score matched HR (95% CI) |
| Intraperitoneal drain insertion | No | 656 (96.6) | 23 (3.4) | - | - |
|  | Yes | 736 (93.0) | 55 (7.0) | 0.78 (0.48-1.28, p=0.324) | 0.87 (0.33-2.31, p=0.780) |
| Age | <70 years | 787 (95.2) | 40 (4.8) | - | - |
|  | ≥70 years | 605 (94.1) | 38 (5.9) | 1.26 (0.80-1.99, p=0.319) | 1.58 (0.63-3.97, p=0.330) |
| Sex | Female | 608 (95.4) | 29 (4.6) | - | - |
|  | Male | 784 (94.1) | 49 (5.9) | 0.81 (0.51-1.28, p=0.363) | 0.94 (0.33-2.69, p=0.900) |
| BMI (kg/m^2^) | Underweight-Normal | 539 (93.1) | 40 (6.9) | - | - |
|  | Overweight | 550 (95.8) | 24 (4.2) | 0.59 (0.35-1.00, p=0.048) | 0.58 (0.19-1.75, p=0.340) |
|  | Obese | 303 (95.6) | 14 (4.4) | 0.71 (0.39-1.32, p=0.279) | 0.77 (0.20-2.93, p=0.700) |
| Smoking status | Never | 754 (94.5) | 44 (5.5) | - | - |
|  | Previous | 422 (94.4) | 25 (5.6) | 0.86 (0.52-1.42, p=0.557) | 1.00 (0.35-2.84, p=1.000) |
|  | Current* | 216 (96.0) | 9 (4.0) | 0.94 (0.46-1.93, p=0.861) | 1.35 (0.31-5.94, p=0.690) |
| ASA score | I-II | 955 (95.4) | 46 (4.6) | - | - |
|  | III-V | 437 (93.2) | 32 (6.8) | 0.82 (0.52-1.28, p=0.379) | 0.51 (0.19-1.36, p=0.180) |
| Diabetes Mellitus | No | 1168 (94.9) | 63 (5.1) | - | - |
|  | non-IDDM | 183 (94.8) | 10 (5.2) | 1.20 (0.61-2.36, p=0.594) | 0.76 (0.16-3.51, p=0.720) |
|  | IDDM | 41 (89.1) | 5 (10.9) | 1.85 (0.73-4.67, p=0.195) | 8.44 (1.58-45.26, p=0.013) |
| Previous abdominal surgery | No | 671 (94.0) | 43 (6.0) | - | - |
|  | Yes | 721 (95.4) | 35 (4.6) | 0.69 (0.44-1.09, p=0.113) | 0.75 (0.30-1.88, p=0.530) |
| Anticoagulation therapy | No | 1097 (94.7) | 61 (5.3) | - | - |
|  | Yes | 295 (94.6) | 17 (5.4) | 0.75 (0.43-1.29, p=0.293) | 0.40 (0.11-1.39, p=0.150) |
| Immunosuppression status | No | 1198 (94.8) | 66 (5.2) | - | - |
|  | Yes | 194 (94.2) | 12 (5.8) | 1.39 (0.75-2.58, p=0.302) | 4.15 (1.45-11.89, p=0.008) |
| Underlying pathology | Benign | 413 (93.4) | 29 (6.6) | - | - |
|  | Malignancy | 979 (95.2) | 49 (4.8) | 1.56 (0.97-2.51, p=0.067) | 0.97 (0.30-3.13, p=0.960) |
| Type of surgery | Colon resection | 694 (94.7) | 39 (5.3) | - | - |
|  | Rectum resection | 407 (92.3) | 34 (7.7) | 0.97 (0.61-1.54, p=0.895) | 0.62 (0.20-1.87, p=0.390) |
|  | Stoma formation/closure | 291 (98.3) | 5 (1.7) | 0.42 (0.15-1.18, p=0.100) | 0.14 (0.01-1.52, p=0.110) |
| Operative approach | Minimally invasive | 719 (94.7) | 40 (5.3) | - | - |
|  | Open | 673 (94.7) | 38 (5.3) | 0.79 (0.50-1.23, p=0.294) | 0.76 (0.30-1.94, p=0.570) |
| Perforated bowel | No | 1359 (94.9) | 73 (5.1) | - | - |
|  | Yes | 33 (86.8) | 5 (13.2) | 1.01 (0.41-2.52, p=0.976) | 0.63 (0.11-3.76, p=0.610) |
| Operative contamination | Clean-Contaminated | 1286 (94.7) | 72 (5.3) | - | - |
|  | Contaminated / Dirty | 106 (94.6) | 6 (5.4) | 1.45 (0.62-3.34, p=0.389) | 2.07 (0.39-10.88, p=0.390) |
| Operation duration (minutes) | Mean (SD) | 195.1 (96.0) | 225.1 (107.0) | 1.00 (1.00-1.00, p=0.904) | 1.00 (0.99-1.00, p=0.660) |
| Intraoperative anastomosis | No | 373 (94.7) | 21 (5.3) | - | - |
|  | Yes | 1019 (94.7) | 57 (5.3) | 1.11 (0.67-1.85, p=0.687) | 0.89 (0.32-2.47, p=0.820) |
| Intraoperative vascular or organ injury | No | 1327 (95.1) | 68 (4.9) | - | - |
|  | Yes | 65 (86.7) | 10 (13.3) | 1.65 (0.84-3.23, p=0.143) | 8.70 (1.81-41.93, p=0.007) |
| Intraoperative blood transfusion | No | 1350 (94.9) | 72 (5.1) | - | - |
|  | Yes | 42 (87.5) | 6 (12.5) | 0.94 (0.41-2.18, p=0.885) | 1.18 (0.18-7.69, p=0.860) |
| * Includes those who stopped smoking within 6 weeks  Data are percentages unless otherwise stated.  Abbreviations: HR: Hazard Ratio; CI: Confidence Interval; SD: Standard Deviation; BMI: Body Mass Index; ASA: American Society of Anaesthesiologists; IDDM: Insulin-dependent Diabetes | | | | | |


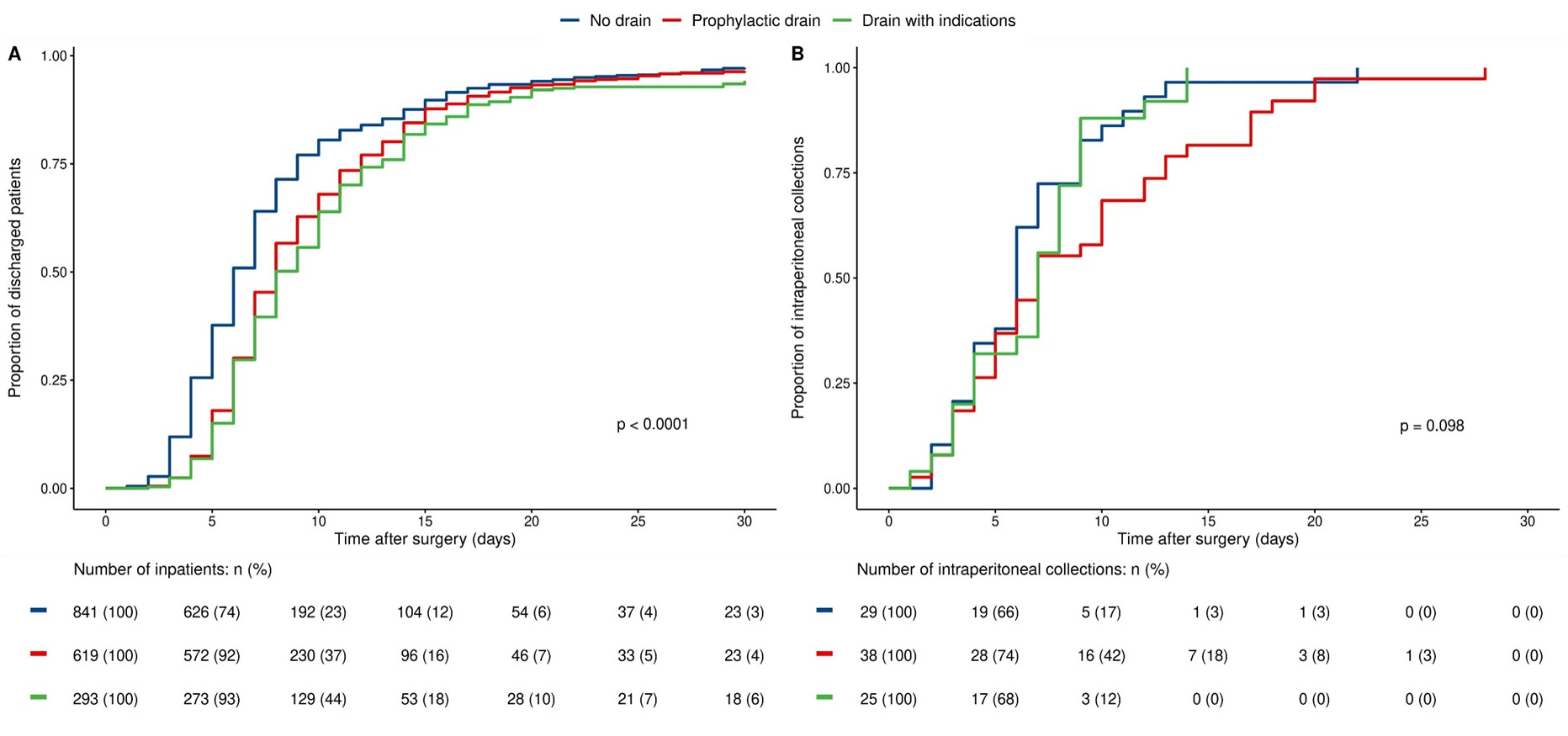
Figure S1: Kaplan-Meier curves stratified by intraperitoneal drain placement: A) time-to-discharge and B) time-to-diagnosis of intraperitoneal collection.
